# Supplementary material for: SHP2 as a primordial epigenetic enzyme expunges histone H3 pTyr-54 to amend androgen receptor homeostasis
Source: Nat Commun. 2024 Jul 4;15:5629. doi: 10.1038/s41467-024-49978-4 (PMC11224269; doi:10.1038/s41467-024-49978-4)
Supplement: Supplementary file 1 — Supplementary Information [file 41467_2024_49978_MOESM1_ESM.pdf]

Supplementary Figure Legends

SHP2 as a Primordial Epigenetic Enzyme Expunges Histone H3 pTyr-54 to Amend Androgen Receptor Homeostasis

Surbhi Chouhan[1,2], Dhivya Sridaran[1,2], Cody Weimholt[3], Jingqin Luo[4,5], Tiandao Li[6], Myles C. Hodgson[7], Luana N. Santos[7], Samantha Le Sommer[7], Bin Fang[8], John M. Koomen[8], Markus Seeliger[9], Cheng-Kui Qu[10], Armelle Yart[11], Maria I. Kontaridis[7,12,13], Kiran Mahajan[1,2], Nupam P. Mahajan[1,2,5]*

Supplementary Figure 1: Validation of pY54-H3 antibody

Supplementary Figure 2: Loss in ACK1 or SHP2 enzymatic activity causes elevation in pY54-H3 levels

Supplementary Figure 3: Detection of ACK1 mediated phosphorylation of SHP2

Supplementary Figure 4: Loss of SHP2 activity regulates deposition of pY54-H3

Supplementary Figure 5: Deposition of pY54-H4 epigenetic marks at the AR gene.

Supplementary Figure 6: *De novo* motif enrichment of pY54-H3 in LNCaP cells and Ack1 KO mice

Supplementary Figure 7: EnrichR analysis of biological processes regulated by pY54-H3 in LNCaP cells. Peaks were identified upon treatment with (*R*)-**9b**.

Supplementary Figure 8: EnrichR analysis of biological processes regulated by pY54-H3 in prostates of Ack1 KO mice.

Supplementary Figure 9: SHP2 inversely regulates pY54-H3 epigenetic marks deposition at the AR gene locus

Supplementary Figure 10: Enrichment of pY54-H4 epigenetic marks at the AR locus upon loss of SHP2 or ACK1 activity

Supplementary Figure 11: Loss of ACK1 or SHP2 activity suppresses AR and PSA transcription.

Supplementary Figure 12: pY580-SHP2 levels directly correlate with progression of disease

Supplementary Figure 13: Increased pY54-H3 and corresponding decrease in pY-SHP2 expression in mice lacking ACK1 or SHP2 activity

Supplementary Figure 14: DHT treatment causes significant increase in AR, and target gene expression in iPSCs derived from a NSML patient

Supplementary Figure 15: pY54-H3 regulatory properties of PTP superfamily of enzymes

Supplementary Figure 16: Other target genes with pY-54 deposition

__Supplementary Tables__

Supplementary Table 1: pY54-H3 peak analysis of prostates of mice treated with (*R*)-**9b**, or WT and KO mice

Supplementary Table 2: List of primers

Supplementary Figure 1a

a

```
                54
42 RPGTVALREIRRYQKSTELLIR Homo sapiens
42 RPGTVALREIRRYQKSTELLIR Ailuropoda melanoleuca
42 RPGTVALREIRRYQKSTELLIR Gallus gallus
42 RPGTVALREIRRYQKSTELLIR Mus musculus
42 RPGTVALREIRRYQKSTELLIR Xenopus laevis
42 RPGTVALREIRRYQKSTELLIR Drosophila hydei
42 YDRTVALREIRRYQKSTELLIR Caenorhabditis elegans
42 TPSELALYEIRKYQRSTDLLIS Saccharomyces cerevisiae
```

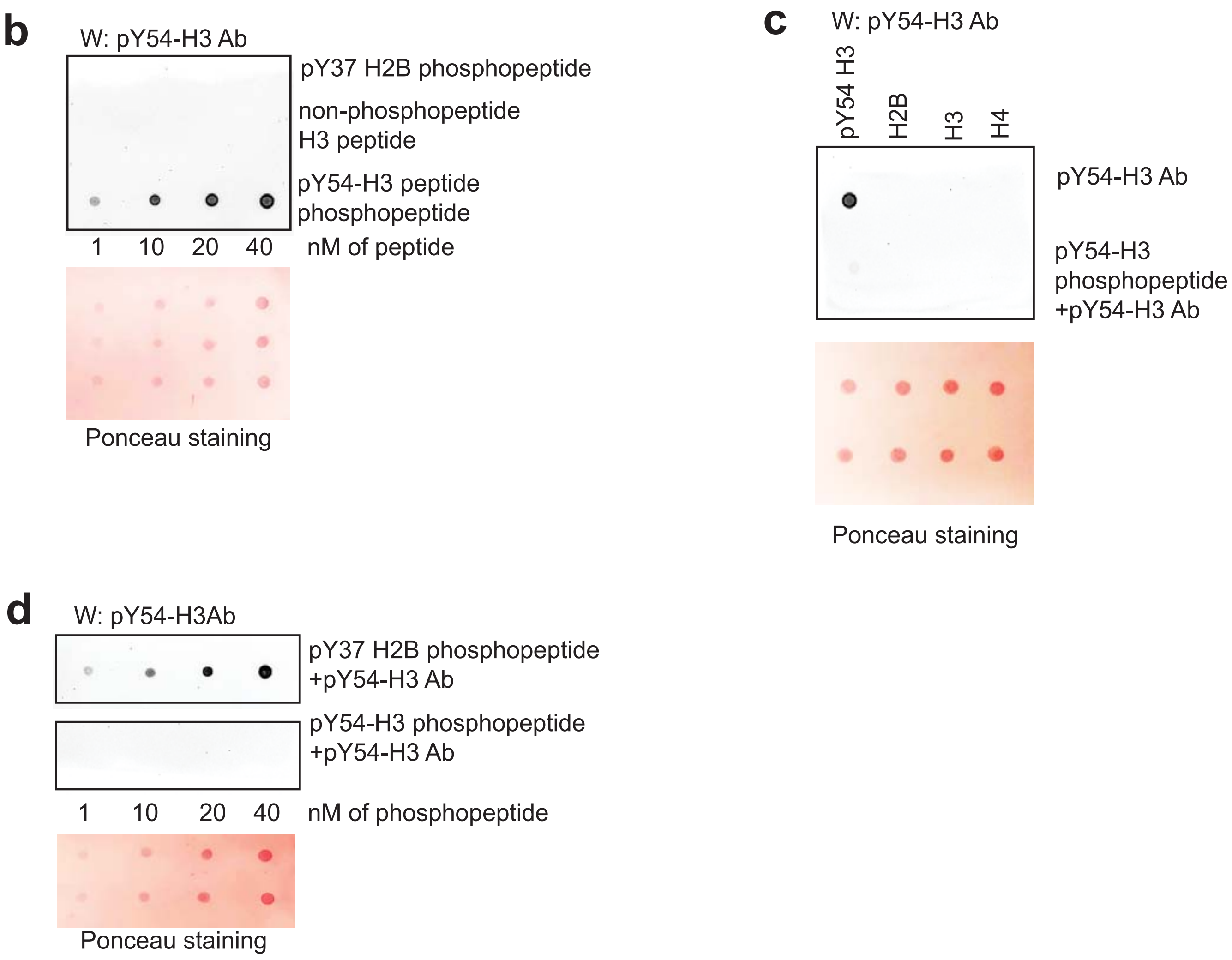

Supplementary Figure 1b

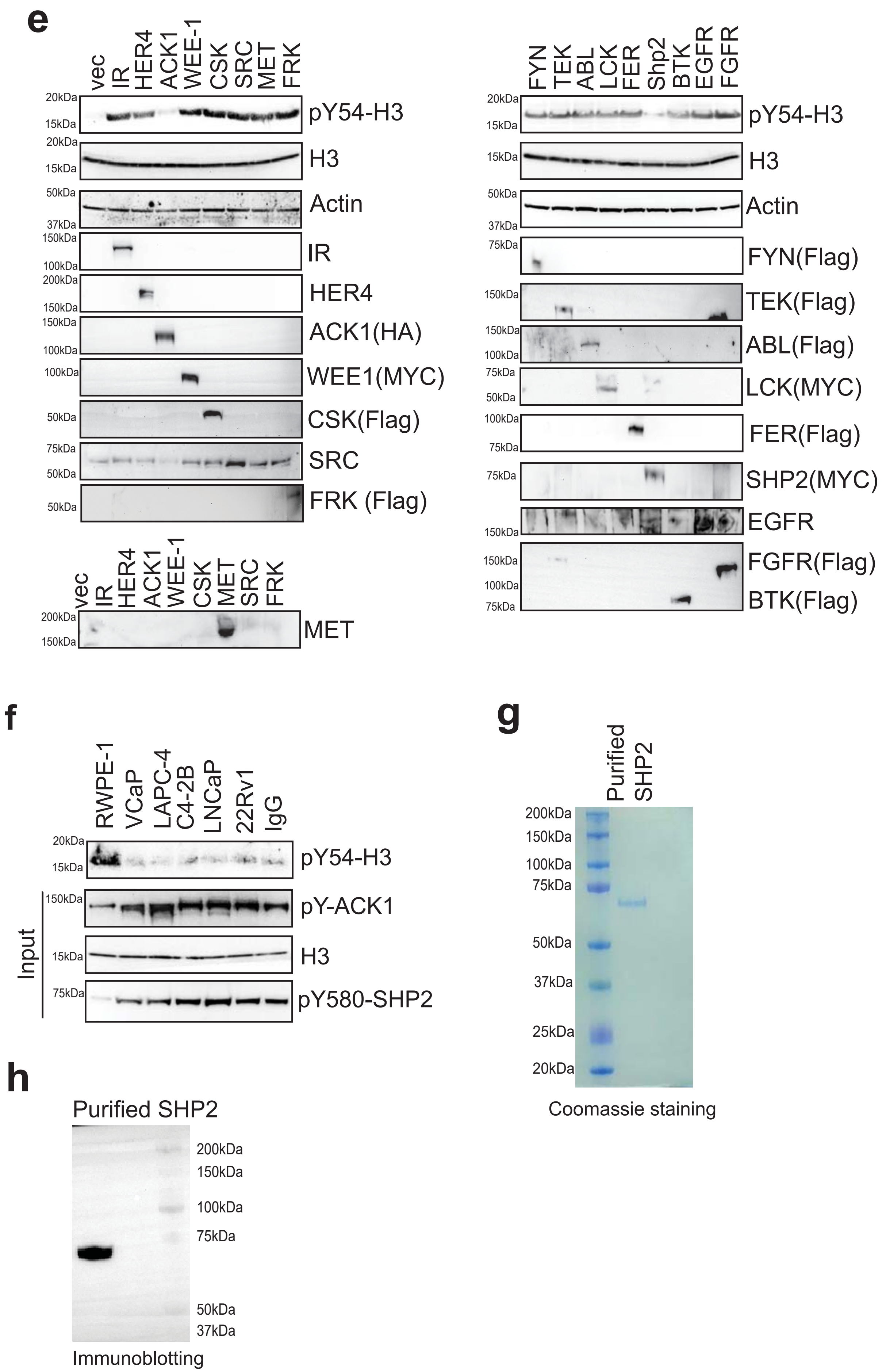

Supplementary Figure 1c

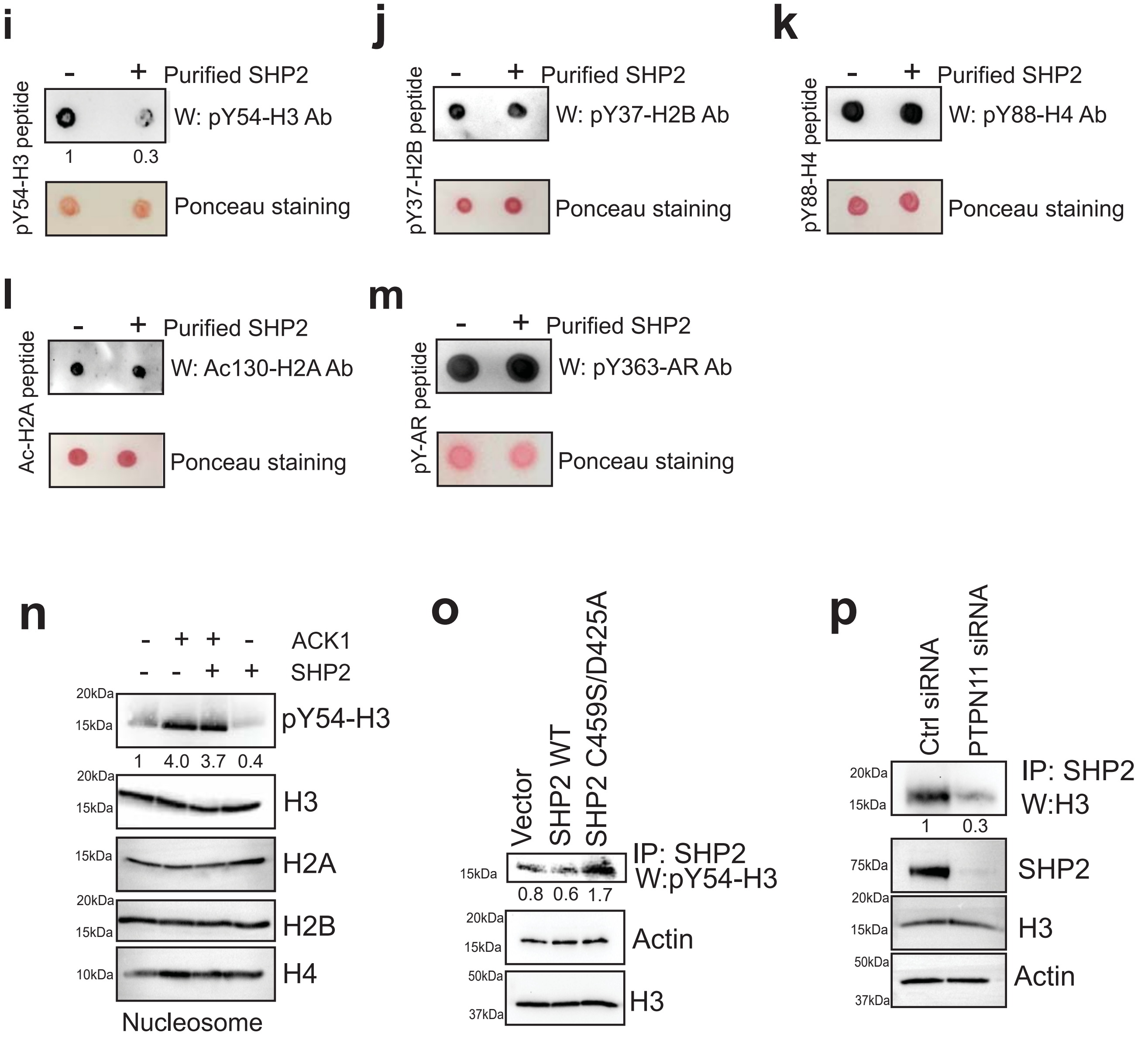

Supplementary Figure 1: Validation of pY54-H3 antibody

a Alignment of histone H3 protein sequence indicates that the Tyr residue at 54 site is invariant from human to yeast. **b** Validation of pY54-H3 antibody. Peptide spanning pY54-H3 EIRRpYQKSTELLIR and identical but unmodified peptide were spotted onto a nitrocellulose membrane in increasing concentrations, followed by immunoblotting with pY54-H3 antibodies. A phospho-peptide derived from histone H2B (pY37-H2B) was used as a negative control (top). The Ponceau staining of the blot is shown (bottom panel). **c** Peptides were spotted onto a nitrocellulose membrane. Prior to probing, the pY54-H3 antibody was pre-incubated with the phosphopeptide. The Ponceau staining of the blot is shown (bottom panel). **d** Peptides were spotted onto a nitrocellulose membrane in increasing concentration, followed by immunoblotting with pY54-H3 antibody. Prior to probing, the pY54-H3 antibody was pre-incubated with the pY54-H3 phosphopeptide (2nd panel) or histone H2B phosphopeptide (the top panel). The Ponceau staining of the blot is shown (bottom panel). **e** HEK293T cells were transfected with Vector or various kinases- and phosphatase-expressing constructs, including IR, HER-4, ACK1, WEE1, CSK, SRC, MET, FRK, FYN, TEK, ABL, LCK, FER, SHP2, BTK, EGFR, FGFR. The lysates were immunoprecipitated (IP) with pY54-H3 antibodies, followed by immunoblotting with H3 antibodies. Lower panels are immunoblots with the indicated antibodies. **f** Lysates from RWPE-1, VCaP, LAPC-4, C4-2B, LNCaP, and 22Rv1 cells were IP with pY54-H3 antibodies, followed by immunoblotting with H3 antibodies (top panel). Lower panels are immunoblots with the indicated antibodies. **g** SHP2 was purified using FLAG beads from HEK293T cells transfected with FLAG-tagged SHP2 construct, separated on SDS-PAGE and stained with Coomassie blue. **h** Immunoblotting of purified SHP2. **i-m** Purified SHP2 was incubated with phosphopeptides or acetylK130-peptide, followed by immunoblotting with indicated antibodies (the top panel). The Ponceau staining of the peptides are shown (bottom panel). **n** Nucleosomes were incubated with purified ACK1, followed by incubation with purified SHP2. Immunoblotting was performed as indicated. **o** VCaP cells were retrovirally infected with SHP2 WT, and SHP2 C459S/D425A (Substrate trapping mutant) constructs and lysates were IP with SHP2 antibody, followed by immunoblotting with pY54-H3 antibody (top panel). Lower panels are immunoblots with the indicated antibodies. **p** VCaP cells were transfected with *PTPN11* siRNAs and lysates were IP with SHP2 antibody, followed by immunoblotting with H3 antibody (top panel). Lower panels are immunoblots with the indicated antibodies. Representative images are shown from $n = 3$ biologically independent experiments. Source data are provided as a Source Data file.

Supplementary Figure 2

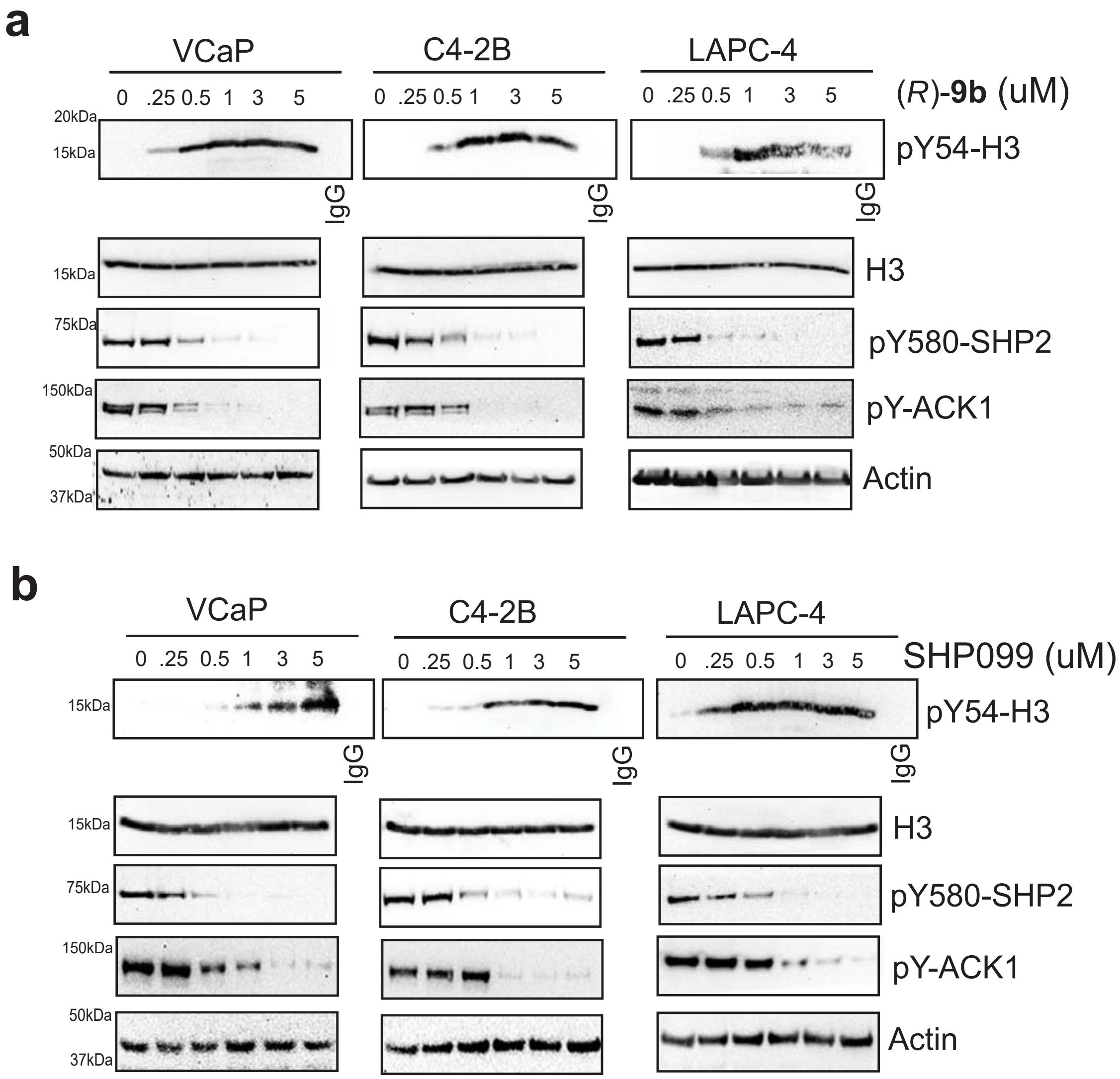

Supplementary Figure 2: Loss in ACK1 or SHP2 enzymatic activity causes elevation in pY54-H3 levels

a VCaP, C4-2B and LAPC-4 cells were treated with the indicated concentrations of (*R*)-**9b** for 18 h and lysates were IP with pY54-H3 antibodies, followed by immunoblotting with H3 antibodies (top panel). Lower panels are immunoblots with the indicated antibodies. **b** VCaP, C4-2B and LAPC-4 cells were treated with the indicated concentrations of SHP099 for 18 h and lysates were IP with pY54-H3 antibodies, followed by immunoblotting with H3 antibodies (top panel). Lower panels are immunoblots with the indicated antibodies. For **a** and **b**, representative images are shown from n = 3 biologically independent experiments. Source data are provided as a Source Data file.

Supplementary Figure 3

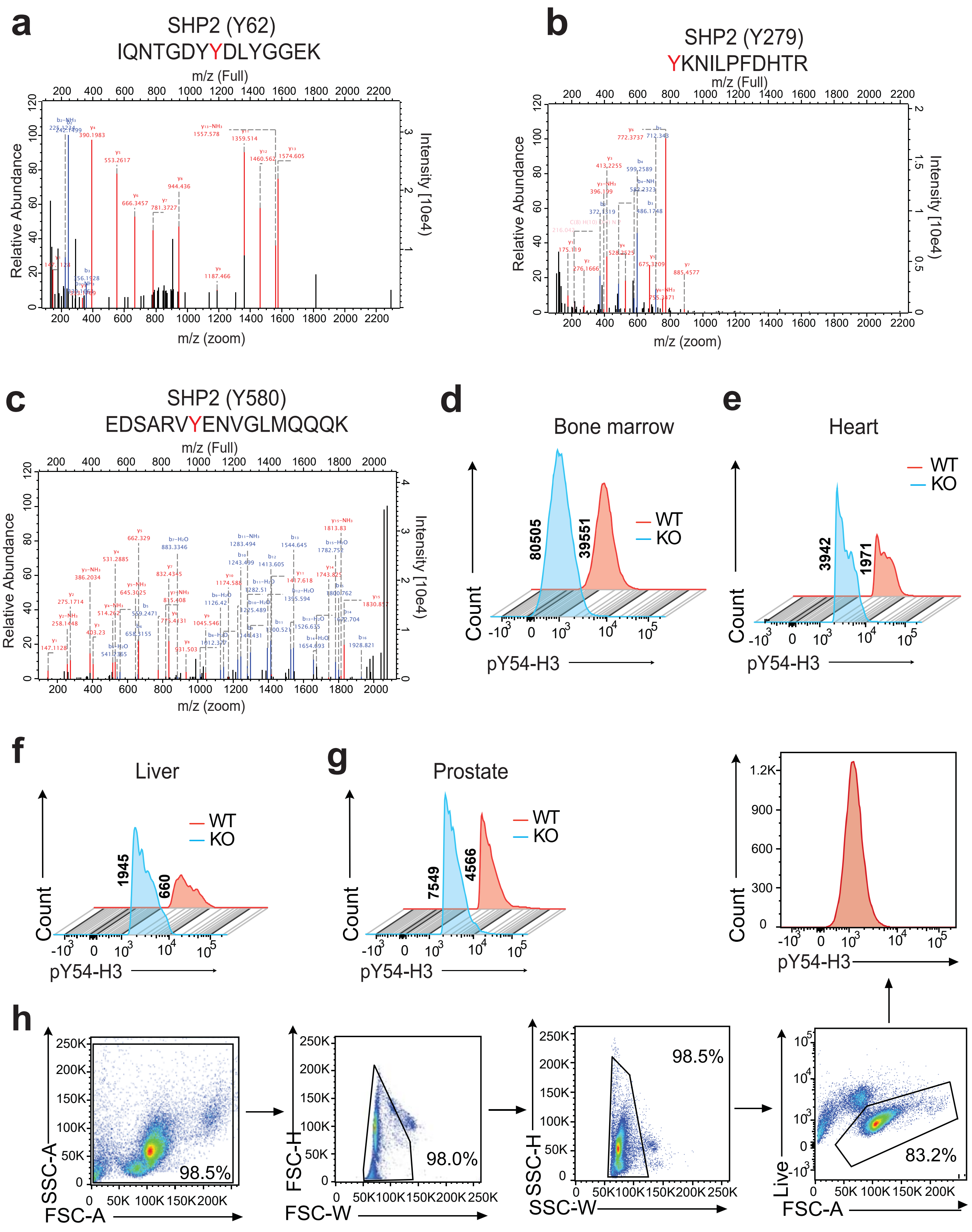

Supplementary Figure 3: Detection of ACK1 mediated phosphorylation of SHP2

a-c C4-2B cells were transfected with ACK1 and lysates were immunoprecipitated with pTyr beads and processed for LC-MS/MS analysis. Peptides IQNTGDpYYDLYGGEK (pY62), pYKNILPFDHTR (pY279), EDSARVpYENVGLMQQQK (pY580) from SHP2 protein were detected. MS/MS spectrum confirmed the peptide sequence. Identification was made with MaxQuant software. **d-g** Cells derived from bone marrow, heart, liver and prostate of WT and *Ack1* KO mice were stained with pY54-H3 antibodies, followed by flow cytometry (*n*=3 mice in each group). Representative histograms are shown. **h** Representative gating strategy of pY54-H3 expression gated on the live cell population isolated from the organs of the WT and *Ack1* KO mice.

Supplementary Figure 4

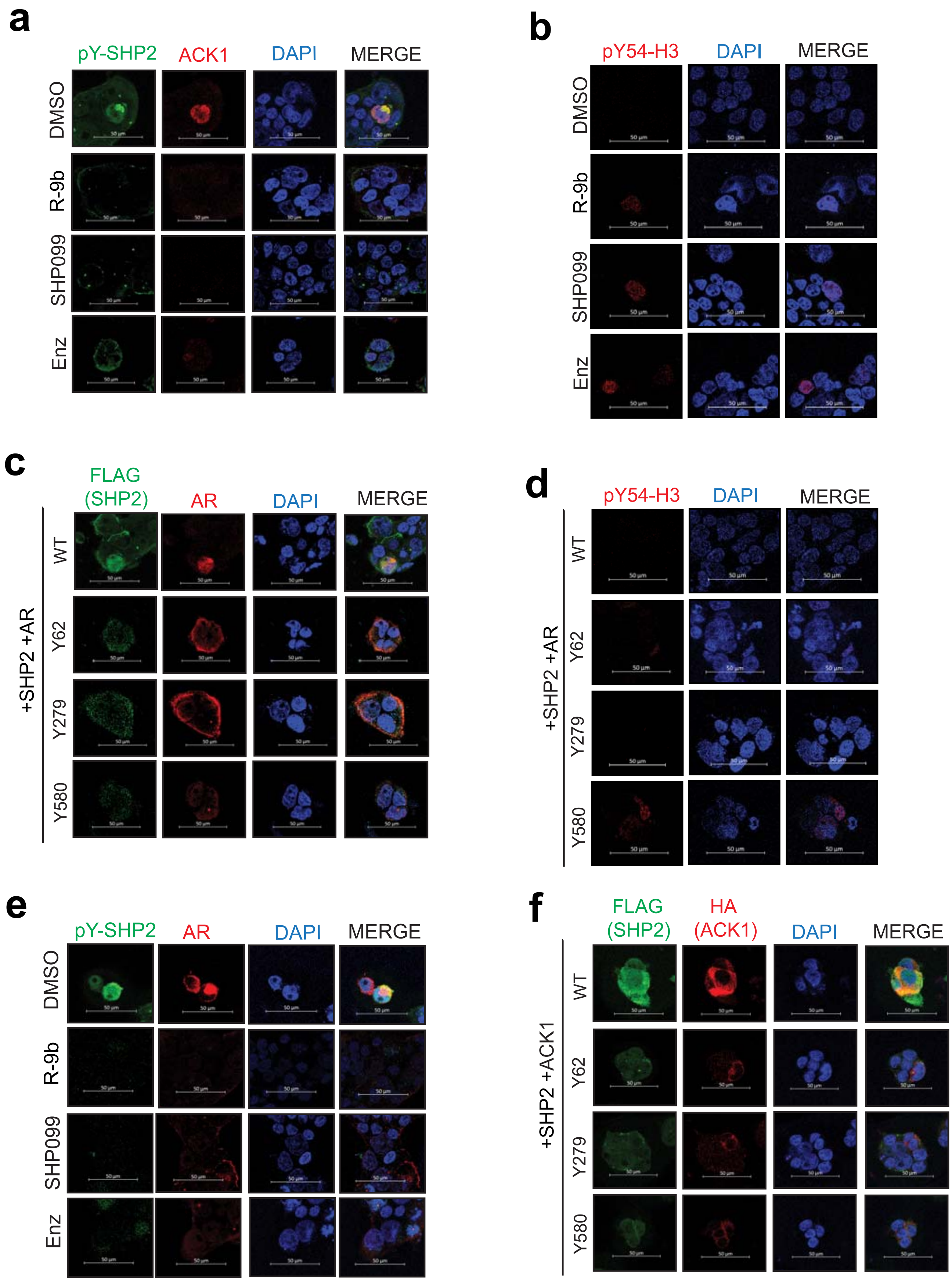

Supplementary Figure 4: Loss of SHP2 activity regulates deposition of pY54-H3

a, b VCaP cells were treated with 1 μM of (R)-9b, SHP099, or enzalutamide (Enz), for 18 h. Cells were then co-stained with pY-SHP2, pY54-H3 and ACK1 antibody, and were processed for immunofluorescence-based detection. Bars represent 50 μm. **c, d** HEK293T cells were co-transfected with HA-tagged ACK1 and FLAG-tagged SHP2 or SHP2-Y62F, -Y279F or -Y580F. Cells were then co-stained with FLAG (SHP2), AR and pY54-H3 antibodies and were processed for immunofluorescence-based detection. Bars represent 50 μm. **e** VCaP cells were treated with 1 μM of (R)-9b, SHP099, or enzalutamide (Enz), for 18 h. Cells were then co-stained with pY-SHP2 or AR antibody and were processed for immunofluorescence based detection. Bars represent 50 μm. **f** HEK293T cells were co-transfected with HA-tagged ACK1 and FLAG-tagged SHP2 or SHP2-Y62F, -Y279F or -Y580F. Cells were then co-stained with FLAG (SHP2) and HA (ACK1) antibodies and were processed for immunofluorescence-based detection. Bars represent 50 μm. For **a**-**f**, representative images are shown from *n* = 3 biologically independent experiments. Source data are provided as a Source Data file.

Supplementary Figure 5

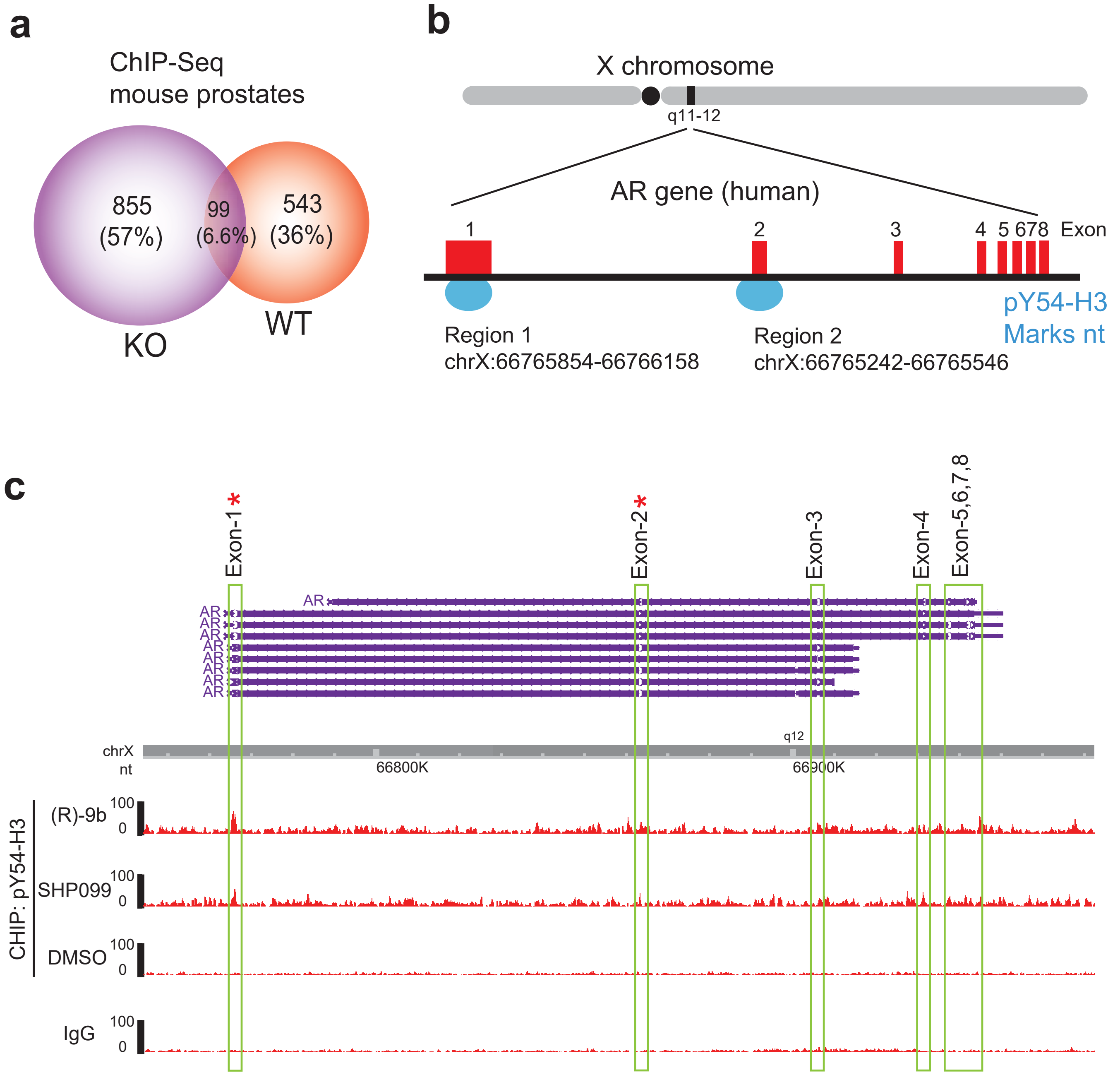

Supplementary Figure 5: Deposition of pY54-H4 epigenetic marks at the *AR* gene.

a Lysates prepared from the prostates of WT and *Ack1* KO mice were subjected to ChIP using pY54-H3 antibody (or IgG), followed by sequencing. Overlapping sequencing peaks from these samples are represented in the Venn diagram. **b** ChIP-seq was performed using pY54-H3 antibody or IgG. The peaks at exon1 and 2 of the human *AR* locus are shown in graphical format. Numbers indicate nucleotide positions at the midpoint of the peaks. **c** Peak regions for all the *AR* exons are highlighted. Asterisks in red represent peaks that were amplified using PCR primers.

Supplementary Figure 6

A. Homer Known Motif Enrichment Results (LNCaP_9b_vs_CTL_motif)

Total Target Sequences = 64, Total Background Sequences = 35083

Rank	Motif	Name	P-value	log P-pvalue	q-value (Benjamini)	# Target Sequences with Motif	% of Targets Sequences with Motif	# Background Sequences with Motif	% of Background Sequences with Motif	Motif File	SVG
1	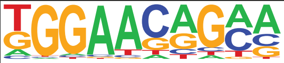	ZNF189(Zf)/HEK293-ZNF189.GFP-ChIP-Seq(GSE58341)/Homer	1e-7	-1.790e+01	0.0000	20.0	31.25%	2575.1	7.34%	motif file (matrix)	svg
2	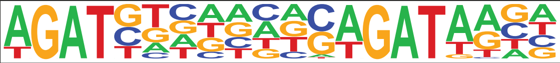	GATA3(Zf),DR8/iTreg-Gata3-ChIP-Seq(GSE20898)/Homer	1e-5	-1.318e+01	0.0004	8.0	12.50%	456.9	1.30%	motif file (matrix)	svg
3	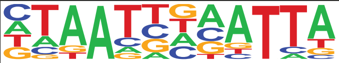	Prop1(Homeobox)/GHFT1-PROP1.biotin-ChIP-Seq(GSE77302)/Homer	1e-5	-1.211e+01	0.0008	21.0	32.81%	4040.7	11.52%	motif file (matrix)	svg
4	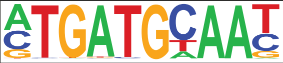	Atf4(bZIP)/MEF-Atf4-ChIP-Seq(GSE35681)/Homer	1e-3	-8.412e+00	0.0238	10.0	15.62%	1406.5	4.01%	motif file (matrix)	svg
5	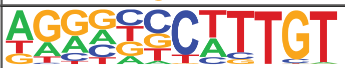	Sox9(HMG)/Limb-SOX9-ChIP-Seq(GSE73225)/Homer	1e-2	-6.650e+00	0.1107	12.0	18.75%	2419.6	6.90%	motif file (matrix)	svg
6	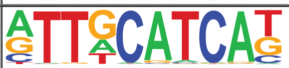	Chop(bZIP)/MEF-Chop-ChIP-Seq(GSE35681)/Homer	1e-2	-6.501e+00	0.1107	8.0	12.50%	1200.7	3.42%	motif file (matrix)	svg
7	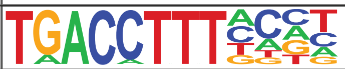	Nur77(NR)/K562-NR4A1-ChIP-Seq(GSE31363)/Homer	1e-2	-5.953e+00	0.1588	5.0	7.81%	518.4	1.48%	motif file (matrix)	svg
8	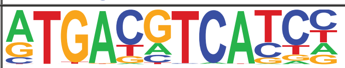	c-Jun-CRE(bZIP)/K562-cJun-ChIP-Seq(GSE31477)/Homer	1e-2	-5.897e+00	0.1588	9.0	14.06%	1631.4	4.65%	motif file (matrix)	svg
9	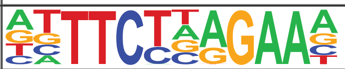	STAT5(Stat)/mCD4+-Stat5-ChIP-Seq(GSE12346)/Homer	1e-2	-5.877e+00	0.1588	8.0	12.50%	1327.3	3.78%	motif file (matrix)	svg
10	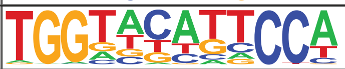	PRDM10(Zf)/HEK293-PRDM10.eGFP-ChIP-Seq(Encode)/Homer	1e-2	-5.756e+00	0.1588	9.0	14.06%	1666.9	4.75%	motif file (matrix)	svg
11	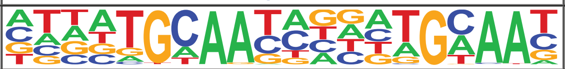	CEBP:CEBP(bZIP)/MEF-Chop-ChIP-Seq(GSE35681)/Homer	1e-2	-5.223e+00	0.2098	5.0	7.81%	616.1	1.76%	motif file (matrix)	svg
12	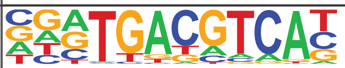	Atf7(bZIP)/3T3L1-Atf7-ChIP-Seq(GSE56872)/Homer	1e-2	-5.062e+00	0.2258	9.0	14.06%	1854.2	5.28%	motif file (matrix)	svg
13	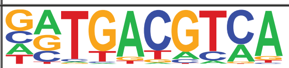	Atf1(bZIP)/K562-ATF1-ChIP-Seq(GSE31477)/Homer	1e-2	-5.054e+00	0.2258	11.0	17.19%	2566.7	7.32%	motif file (matrix)	svg

B. Homer Known Motif Enrichment Results (Prostate ACK1 KO_vs_Input_motif)

Total Target Sequences = 967, Total Background Sequences = 47017

Rank	Motif	Name	P-value	log P-pvalue	q-value (Benjamini)	# Target Sequences with Motif	% of Targets Sequences with Motif	# Background Sequences with Motif	% of Background Sequences with Motif	Motif File	SVG
1	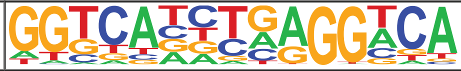	THRa(NR)/C17.2-THRa-ChIP-Seq(GSE38347)/Homer	1e-2	-5.976e+00	1.0000	44.0	4.55%	1354.1	2.88%	motif file (matrix)	svg
2	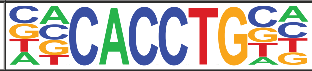	E2A(bHLH),near_PU.1/Bcell-PU.1-ChIP-Seq(GSE21512)/Homer	1e-2	-5.526e+00	1.0000	149.0	15.39%	5848.8	12.45%	motif file (matrix)	svg
3	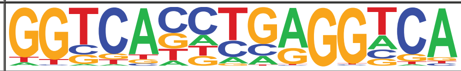	THRb(NR)/HepG2-THRb.Flag-ChIP-Seq(Encode)/Homer	1e-2	-5.374e+00	1.0000	54.0	5.58%	1801.6	3.84%	motif file (matrix)	svg
4	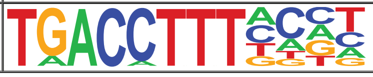	Nur77(NR)/K562-NR4A1-ChIP-Seq(GSE31363)/Homer	1e-2	-5.047e+00	1.0000	26.0	2.69%	734.9	1.56%	motif file (matrix)	svg

C. Homer Known Motif Enrichment Results (Prostate Mice 9b_vs_input_motif)

Total Target Sequences = 39, Total Background Sequences = 46841

Rank	Motif	Name	P-value	log P-pvalue	q-value (Benjamini)	# Target Sequences with Motif	% of Targets Sequences with Motif	# Background Sequences with Motif	% of Background Sequences with Motif	Motif File	SVG
1	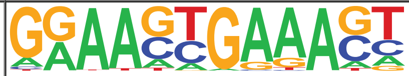	IRF8(IRF)/BMDM-IRF8-ChIP-Seq(GSE77884)/Homer	1e-3	-7.562e+00	0.2226	7.0	17.95%	1737.8	3.71%	motif file (matrix)	svg
2	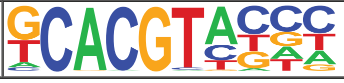	HIF2a(bHLH)/785_O-HIF2a-ChIP-Seq(GSE34871)/Homer	1e-2	-6.094e+00	0.4828	5.0	12.82%	1118.0	2.39%	motif file (matrix)	svg

Supplementary Figure 6: *De novo* motif enrichment of pY54-H3 in LNCaP cells and *Ack1* KO mice

a-**c** pY54-H3 ChIP-sequencing data from LNCaP cell treated with (*R*)-**9b** (**a**), *Ack1* KO mice (**b**) and mice treated with (*R*)-**9b** (**c**), was examined for *de novo* transcription-factor binding motifs (analysis was performed using HOMER). Significantly enriched motifs and associated P values are shown. Motif enrichment is calculated using either the cumulative hypergeometric or cumulative binomial distributions.

Supplementary Figure 7.1

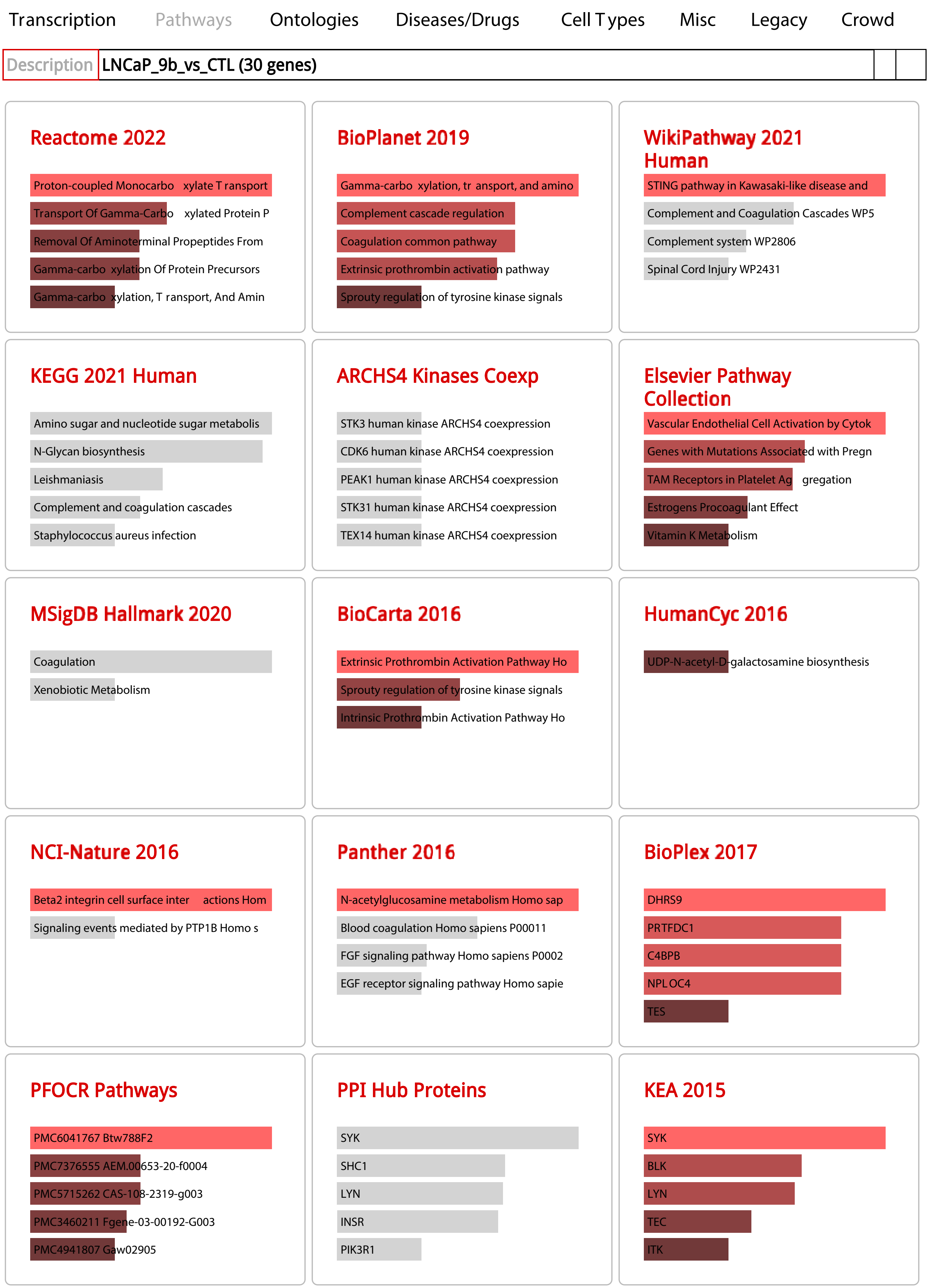

Supplementary Figure 7.2

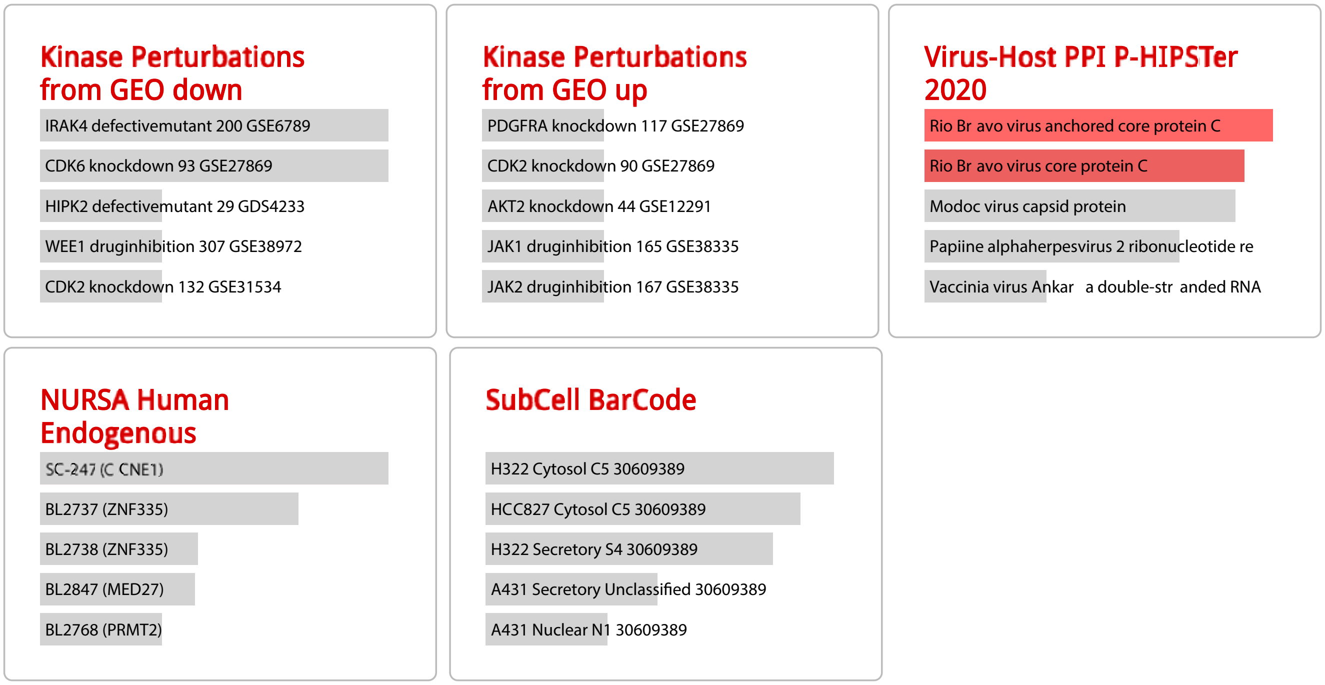

Supplementary Figure 7: EnrichR analysis of biological processes regulated by pY54-H3 in LNCaP cells. Peaks were identified upon treatment with (*R*)-**9b**.

Supplementary Figure 8.1

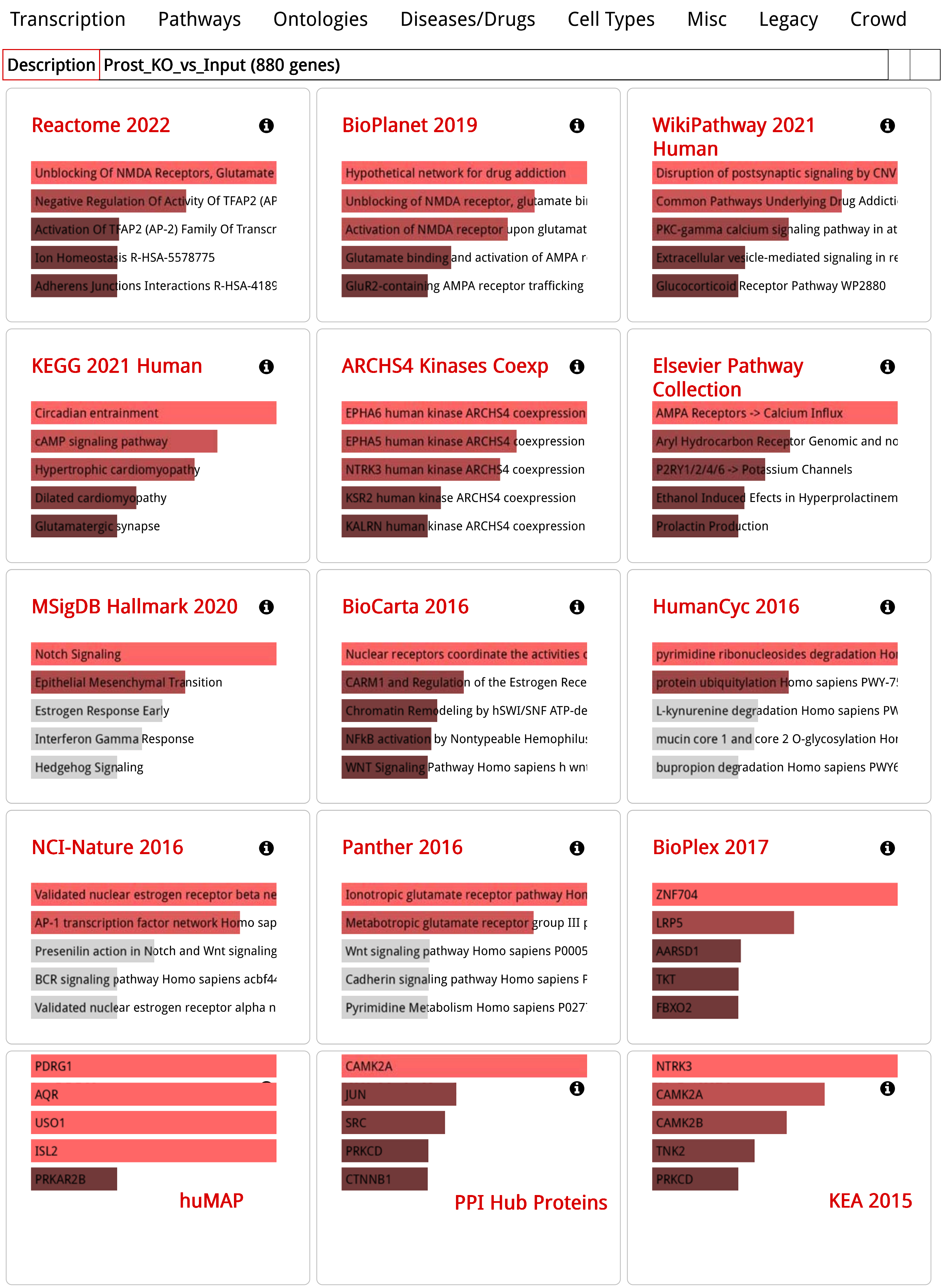

Supplementary Figure 8.2

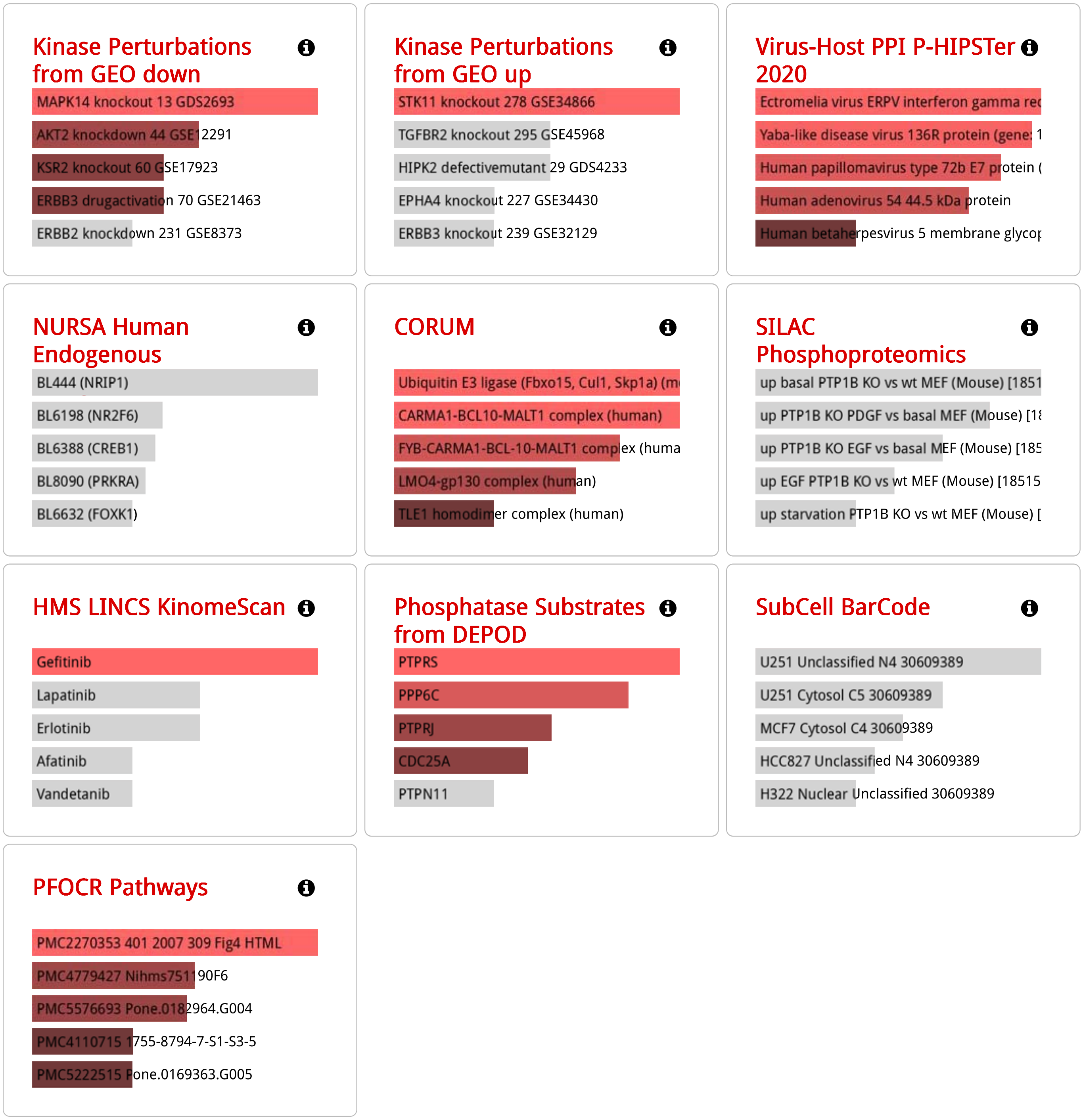

Supplementary Figure 8: EnrichR analysis of biological processes regulated by pY54-H3 in prostates of *Ack1* KO mice.

Supplementary Figure 9

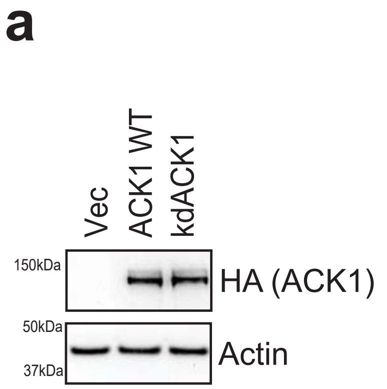

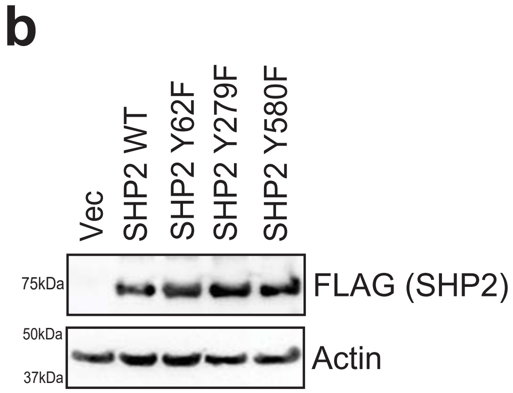

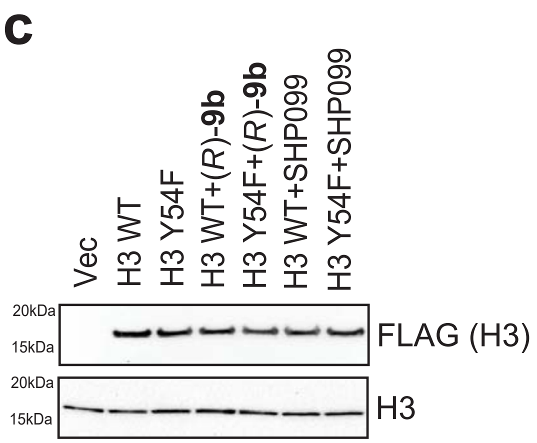

Supplementary Figure 9: SHP2 inversely regulates pY54-H3 epigenetic marks deposition at the *AR* gene locus

a VCaP cells were co-transfected with HA-tagged ACK1 or kdACK1 expressing constructs and lysates were subjected to immunoblotting to assess equal expression of ACK1 or kdACK1. **b** VCaP cells were co-transfected with FLAG-tagged SHP2 or SHP2-Y62F, -Y279F or -Y580F mutants. The lysates were subjected immunoblotting to assess equal expression of SHP2 and mutants. **c** LAPC-4 cells were transfected with FLAG-tagged H3 or H3-Y54F mutant and treated with 1 μM of (*R*)-**9b** or SHP099 for 8 h and lysates were subjected to immunoblotting to assess equal expression of H3 or H3-Y54F mutant. For **a**-**c**, representative images are shown from n = 3 biologically independent experiments. Source data are provided as a Source Data file.

Supplementary Figure 10

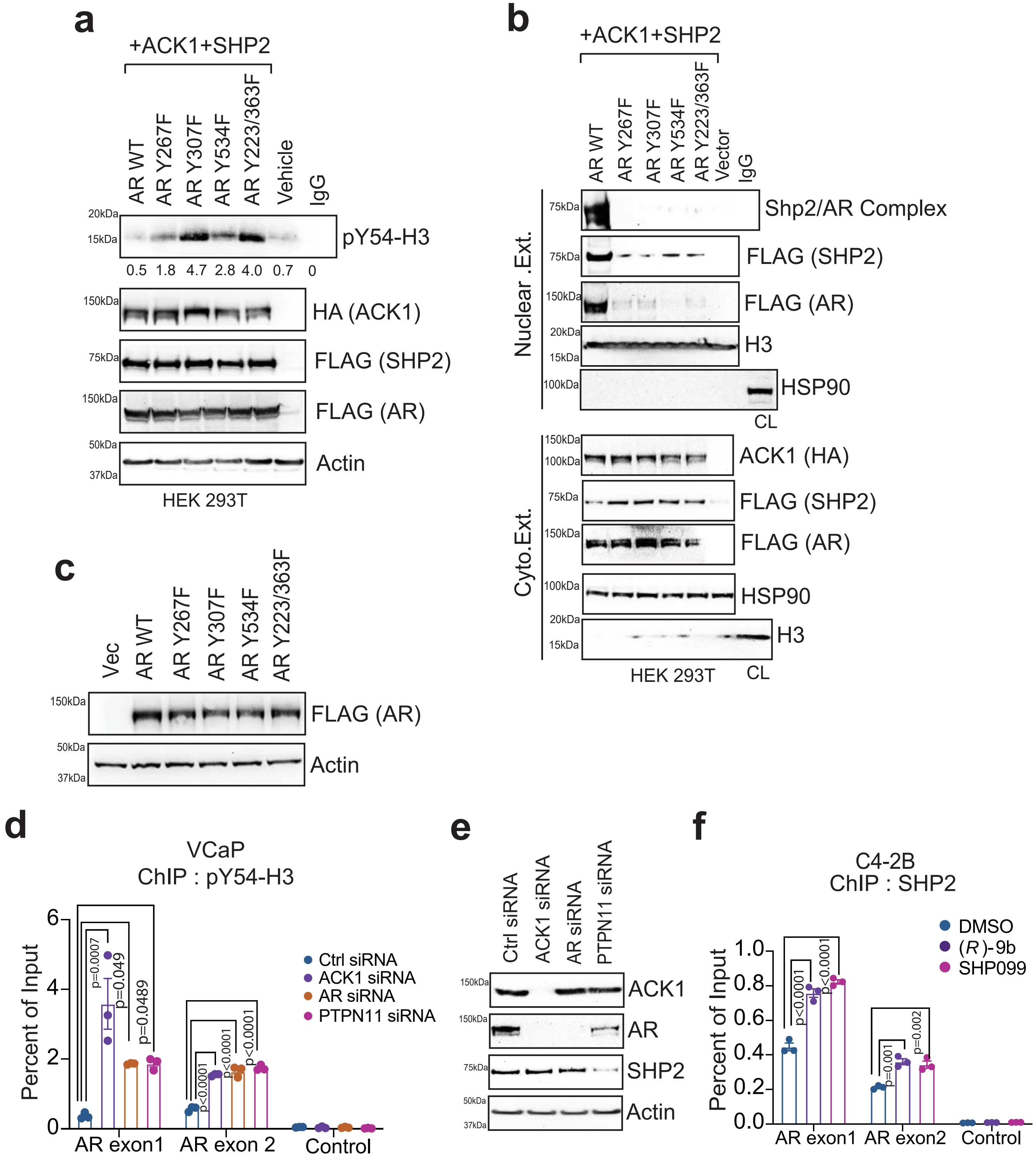

Supplementary Figure 10: Enrichment of pY54-H4 epigenetic marks at the *AR* locus upon loss of SHP2 or ACK1 activity

a HEK293T cells were co-transfected with FLAG-tagged SHP2, HA-tagged ACK1 and FLAG-tagged AR or AR-Y267F, -Y307F, -Y534F, -Y223/363F mutants expressing constructs. Lysates were IP with pY54-H3 antibodies, followed by immunoblotting with H3 antibodies. Lower panels are immunoblots with the indicated antibodies. **b** HEK293T cells were co-transfected with FLAG-tagged SHP2, HA-tagged ACK1 and FLAG-tagged AR or AR-Y267F, -Y307F, -Y534F, -Y223/363F mutants expressing constructs. Nuclear and cytosolic fractionation was performed; nuclear lysates were IP with SHP2 antibodies, followed by immunoblotting with AR antibodies (top panel). Lower panels are immunoblots with the indicated antibodies. **c** LAPC4 cells were co-transfected with HA-tagged ACK1 and FLAG-tagged AR or AR-Y267F, -Y307F, -Y534F, -Y223/363F expressing constructs. Immunoblotting was performed using FLAG antibody to determine equal expression of AR and its mutants. **d** VCaP cells were transfected *TNK2*, *AR* and *PTPN11* siRNA and ChIP was performed using pY54-H3 antibody, followed by qPCR using primers corresponding to *AR* exon 1, 2 or control region. **e** C4-2B cells were transfected *TNK2*, *AR* and *PTPN11* siRNA and immunoblotting was performed with indicated antibodies. **f** C4-2B cells were treated with 1 μM of (R)-9b or SHP099 for 8 h and lysates were subjected to ChIP with SHP2 antibody, followed by qPCR using primers corresponding to AR exon 1, 2, or control (IGX, gene desert on chromosome 12) region. For **a**-**c**, **e**, representative images are shown from *n* = 3 biologically independent experiments. For **d** and **f**, data are represented as mean ± SEM (n = 3 biologically independent samples, three replicates). p values were determined by one-way ANOVA. p values are shown on the graph. Source data are provided as a Source Data file.

Supplementary Figure 11

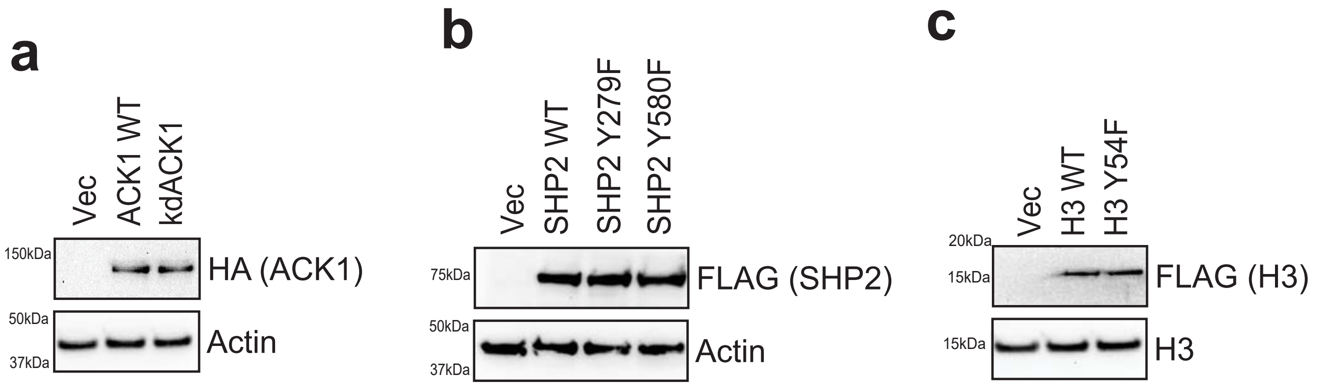

Supplementary Figure 11: Loss of ACK1 or SHP2 activity suppresses *AR* and *PSA* transcription.

a VCaP cells were transfected with HA-tagged ACK1 or kdACK1 and subjected to immunoblotting. **b** VCaP cells were transfected with SHP2 or SHP2 mutant constructs and subjected to immunoblotting. **c** VCaP cells were transfected with H3 or Y54-H3 mutant expressing constructs and subjected to immunoblotting. For **a-c**, representative images are shown from $n = 3$ biologically independent experiments.

Supplementary Figure 12

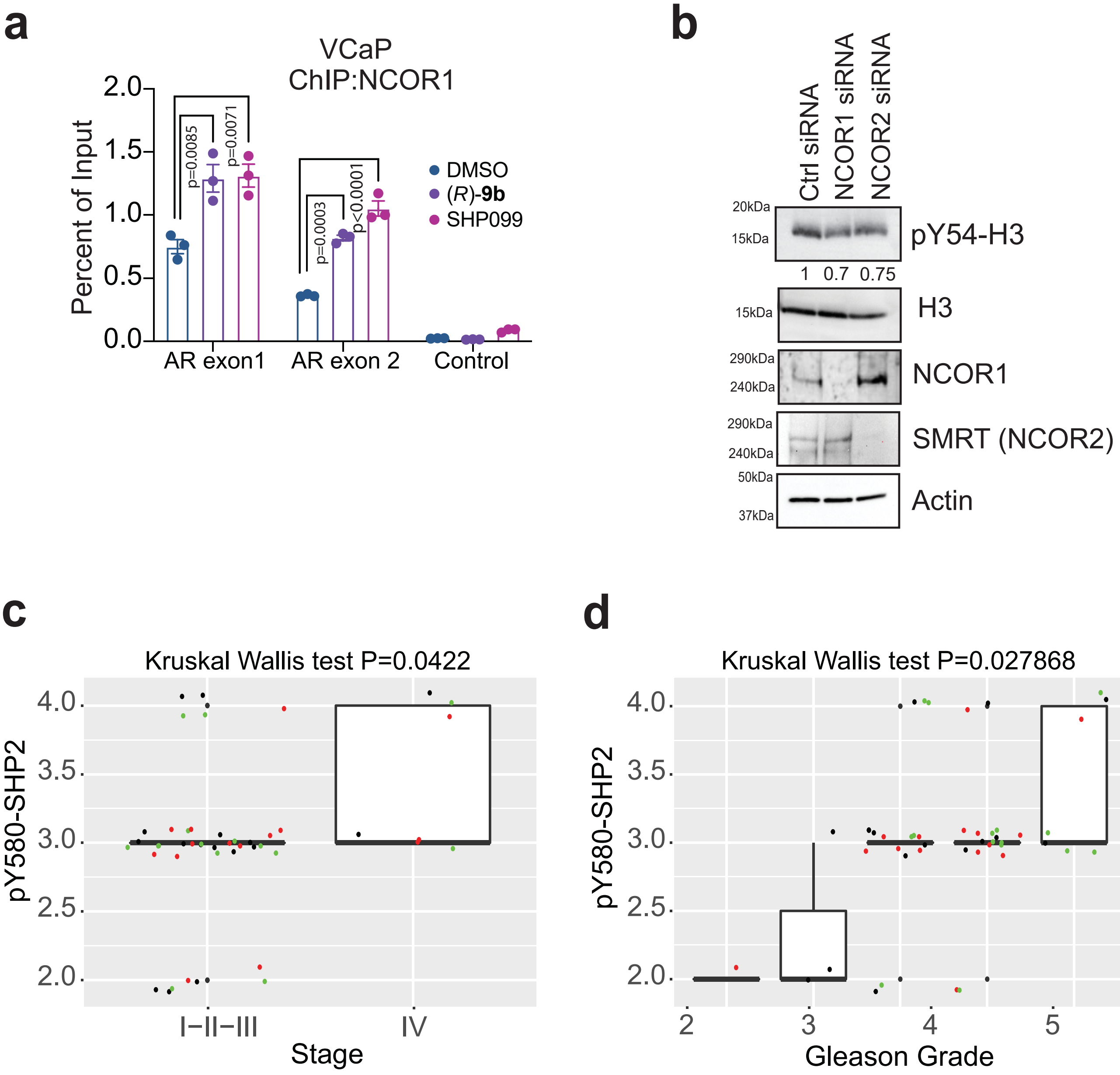

Supplementary Figure 12: pY580-SHP2 levels directly correlate with progression of disease

a VCaP cells were treated with 1 μM of (*R*)-**9b** or SHP099 for 8 h and ChIP was performed using NCoR1 & NCoR2 (SMRT) antibodies, followed by qPCR using primers corresponding to *AR* exon 1, 2 or control region. **b** VCaP cells were transfected with NCoR1 or NCoR2 siRNAs and lysates were IP with SHP2 antibody, followed by immunoblotting with H3 antibody (top panel). Lower panels are immunoblots with the indicated antibodies. **c**, **d** Box plots summarizing the distributions of staining intensity for pY580-SHP2 antibodies in prostate TMA sections (n = 80 cores per slide). The box indicates 50% of the data from the 25% quartile to the 75% quartile with the bold black horizontal lines representing the median. Individual data points were jittered and colored for better visualization. For **b**, data are represented as mean ± SEM (n = 3 biologically independent experiments, three replicates). p values were determined by one-way ANOVA. For **c**, **d**, p values were determined by two-sided Kruskal–Wallis test. p values are shown on the graph. Source data are provided as a Source Data file.

Supplementary Figure 13

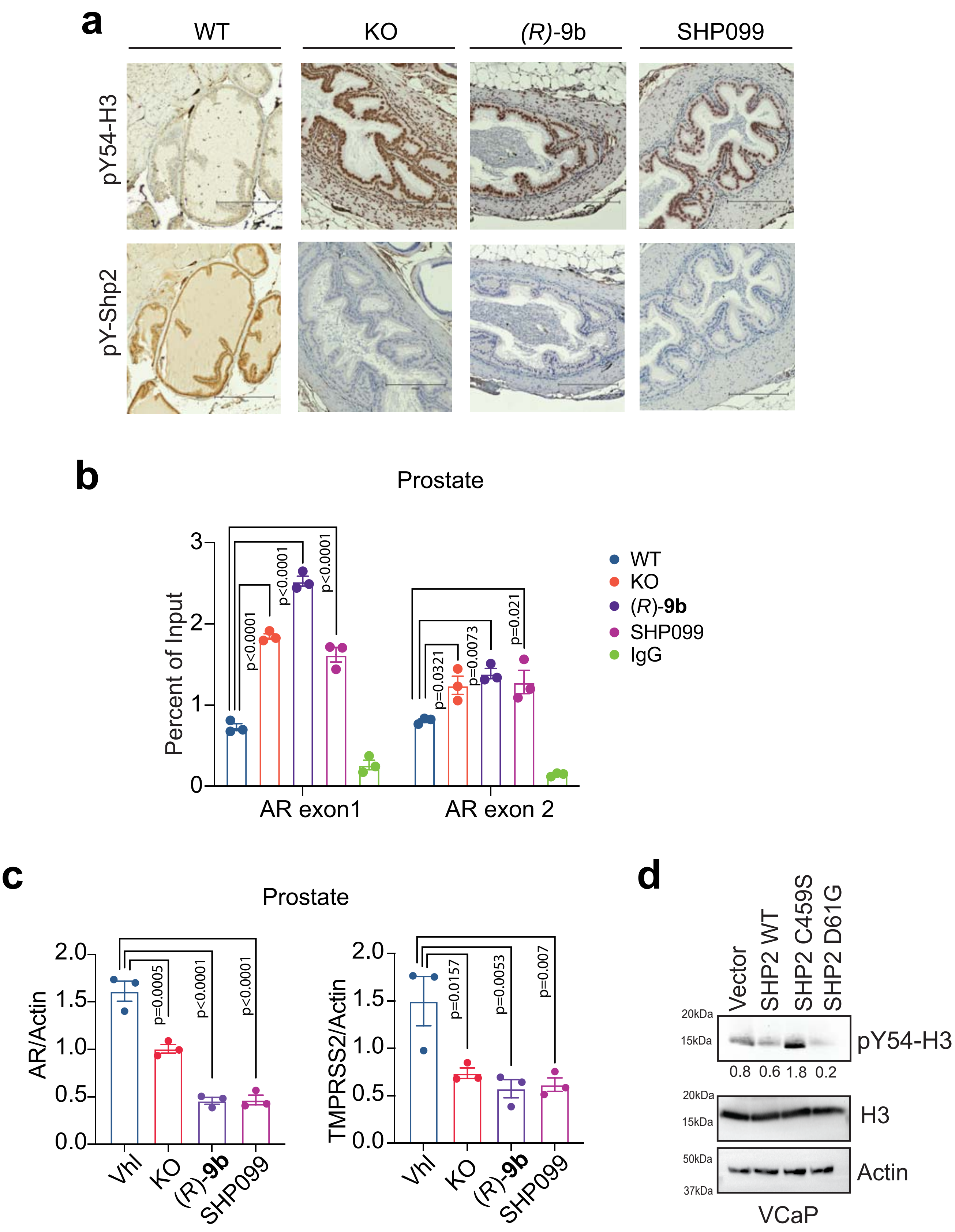

Supplementary Figure 13: Increased pY54-H3 and corresponding decrease in pY-SHP2 expression in mice lacking ACK1 or SHP2 activity

a pY54-H3 and pY580-SHP2 antibody staining of prostates from *Ack1* KO and WT mice, and B6 mice treated with (*R*)-**9b** or SHP099. **b** Lysates from prostates of the mice were subjected to ChIP using pY54-H3 (or IgG as control) antibody, followed by qPCR using primers corresponding to AR exons 1 and 2. **c** Total RNA was isolated from prostates of mice and were subjected to qRT-PCR with AR, TMPRSS2 and actin primers. **d** VCaP cells were retrovirally infected with SHP2 WT, SHP2 C459S (phosphatase dead mutant), SHP2 D61G (Noonan Syndrome mutant) expressing constructs and lysates were IP with SHP2 antibody, followed by immunoblotting with H3 antibody (top panel). Lower panels are immunoblots with the indicated antibodies. Representative images are shown from n = 3 biologically independent experiments. For **b**, **c** data are represented as mean ± SEM (n = 3 biologically independent experiments, three replicates). p values were determined by one-way ANOVA. p values are shown on the graph. Source data are provided as a Source Data file.

Supplementary Figure 14

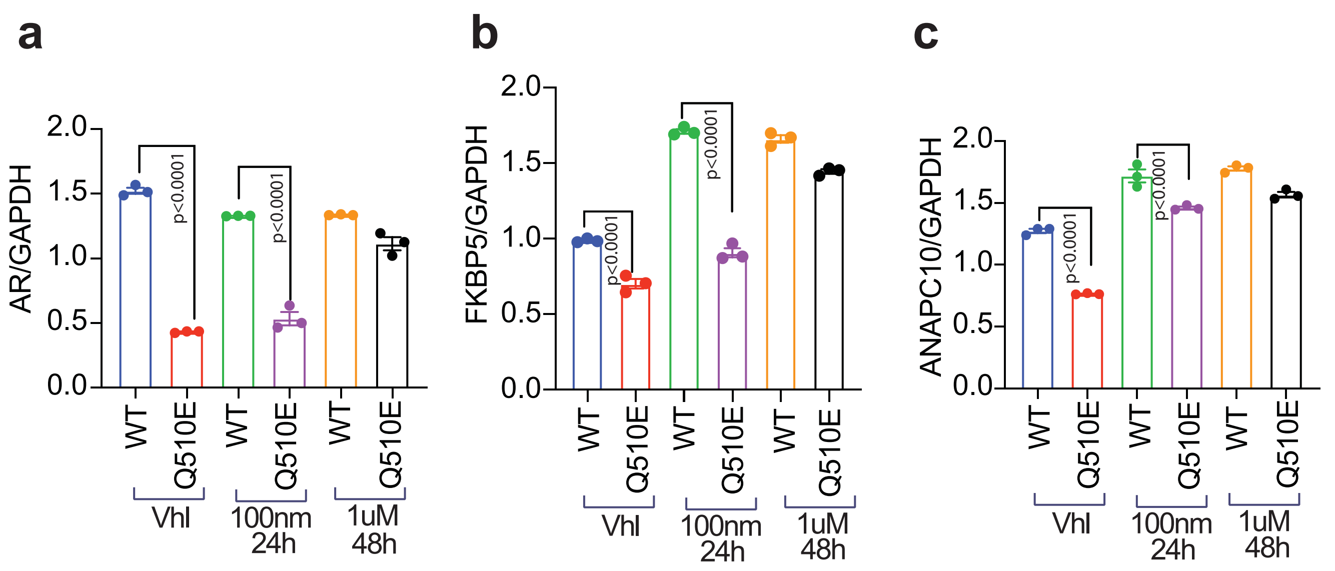

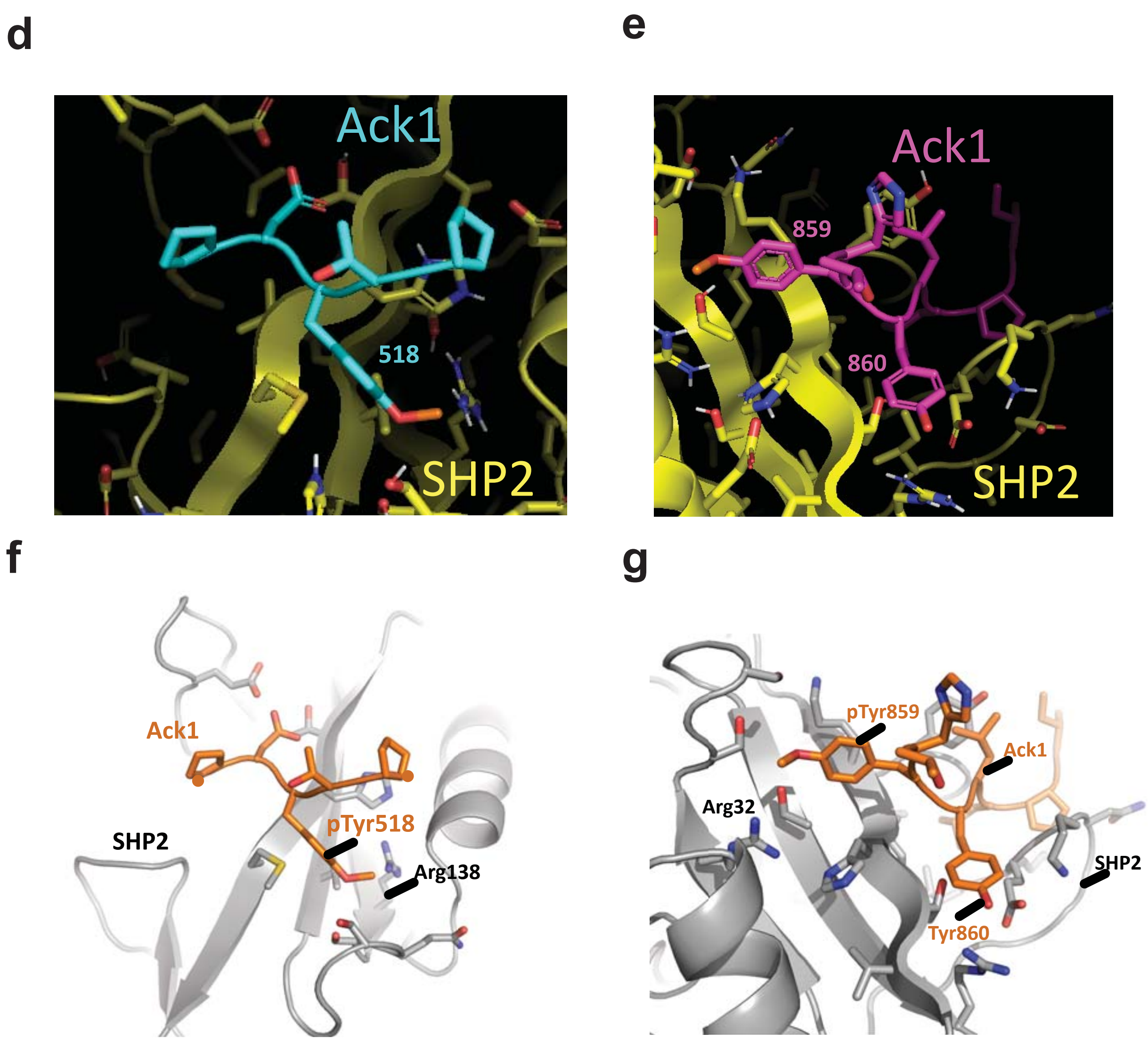

Supplementary Figure 14: DHT treatment causes significant increase in AR, and target gene expression in iPSCs derived from a NSML patient

a-c Total RNA was isolated from iPSCs that were treated with vehicle or DHT (24 and 48 h), followed by qRT-PCR with *AR*, *FKBP5*, *ANAPC10* and GAPDH primers. For **a-c**, p values were determined by unpaired two-tailed Student's *t*-test. *n* = 3 biologically independent experiments. p values are shown on the graph. Source data are provided as a Source Data file.

d-g Structural model of ACK1 phosphopeptides (orange) binding to the SH2 domains of the tyrosine phosphatase SHP2 (gray) (PDB-entry 2SHP). The sequence of the ACK1 phosphopeptides was manually built into the structure of SHP2 based on the phosphotyrosine peptide binding modes observed for the SH2 domains in Src kinase (PDB-entry 1KC2). ACK1 peptide containing pTyr859 binding to the N-terminal SH2 domain of SHP2. pTyr859 could form a salt bridge with Arg32 of ACK1. ACK1 peptide containing pTyr518 binding to the second SH2 domain of SHP2 via a salt bridge with Arg138.

Supplementary Figure 15

a

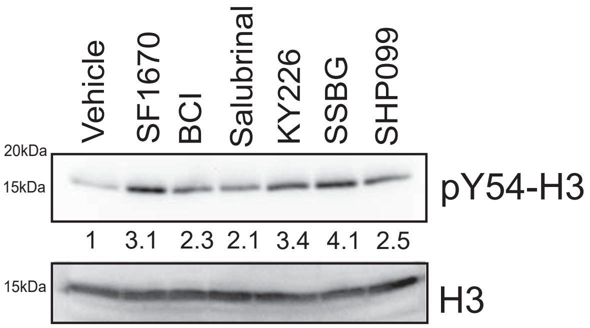

b

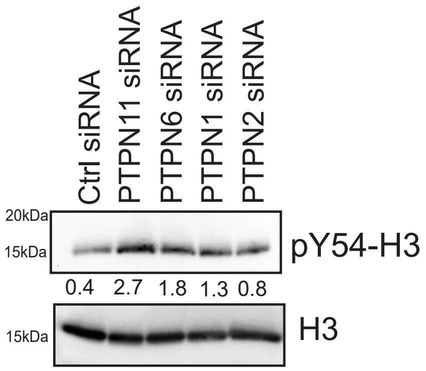

c

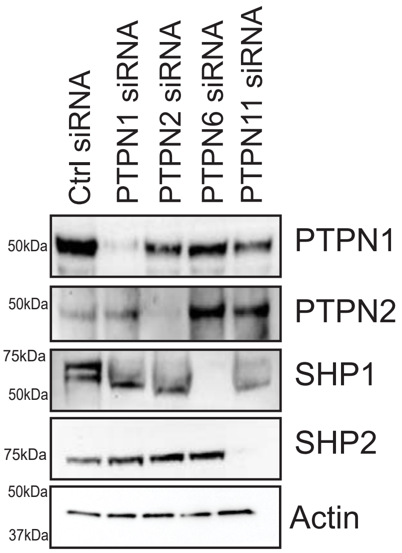

Supplementary Figure 15: pY54-H3 regulatory properties of PTP superfamily of enzymes

a C4-2B cells were treated with various phosphatase inhibitors for 8 hours. Lysates were subjected to IP with pY54-H3 antibody, followed by immunoblotting with H3 antibodies. **b** and **c** VCaP cells were transfected with *PTPN1*, *PTPN2*, *PTPN6* or *PTPN11* siRNAs and lysates were IP with SHP2 antibody, followed by immunoblotting with H3 antibody (top panel). Lower panels are immunoblots with the indicated antibodies. Representative images are shown from $n = 3$ biologically independent experiments. Source data are provided as a Source Data file.

Supplementary Figure 16

a

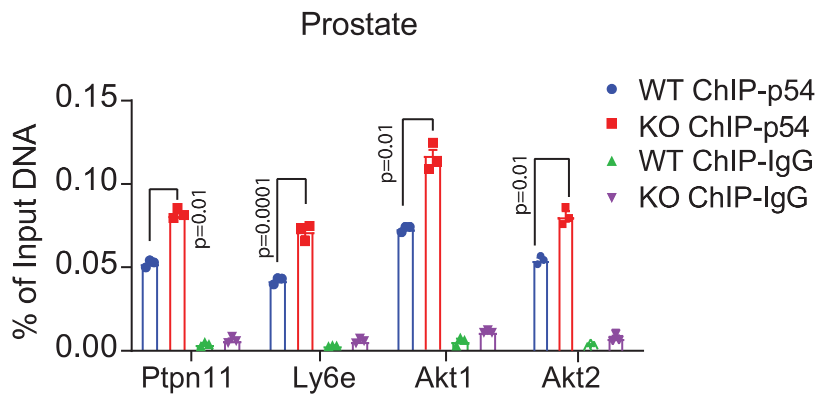

b

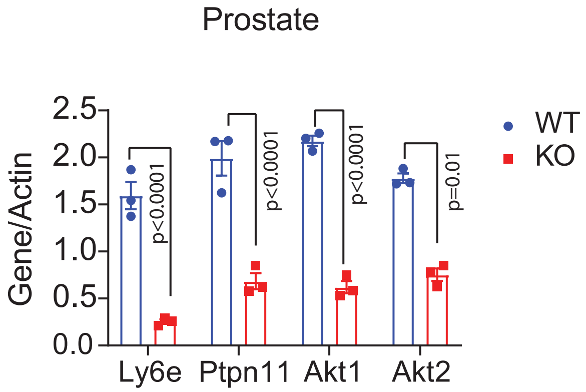

Supplementary Figure 16: Other target genes with pY-54 deposition

a Prostate lysate from WT and *Ack1* KO mice were subjected to ChIP with pY54-H3 antibodies, followed by qPCR using primers corresponding to Ptpn11, Ly6e, Akt1 and Akt2 primers. **b** Total RNA was isolated from Prostates of WT and *Ack1* KO mice, followed by qRT-PCR with Ly6e, Ptpn11, Akt1, Akt2 and Actin primers. For **a**, **b** data are represented as mean ± SEM (n = 3 biologically independent experiments, three replicates). p values were determined by two-tailed Student's *t*-test. p values are shown on the graph. Source data are provided as a Source Data file.

Supplementary Table 1. pY54-H3 peak analysis of prostates of mice treated with (R)-9b, or WT and KO mice

Prostate ChIP Samples	Peak Counts
KO_vs_Input	968
WT_vs_Input	687
(R)-9b_vs_Input	39

Prostate ChIP Samples	**KO**	**WT**	**(R)-9b**
ncRNA	212	126	9
Protein-coding	728	548	29
Pseudo	22	9	0
rRNA	2	2	0
snoRNA	3	2	1

Prostate ChIP Samples	**KO**	**WT**	**(R)-9b**
3'UTR	5	2	1
5'UTR	0	1	0
Exon	13	0	1
Intergenic	571	390	21
Intron	353	277	15
Non-coding	4	4	0
Promoter-TSS	15	8	1
TTS	7	5	0

**Supplementary Table 2**		
**ChIP PCR primers**	**Source**	**Identifier**
Human-IGX1A FP ACGCTGAGAAGAGCTGAAAC	This paper	N/A
Human-IGX1A RP AGCTGCCAAGCACATGAA	This paper	N/A
Human-AR exon 1 FP ACACCAAAGGGCTAGAAGGC	This paper	N/A
Human-AR exon 1 RP GACAGGGTAGACGGCAGTTC	This paper	N/A
Human-AR exon 2 FP GAGGATGGTTCTCCCCAAGC	This paper	N/A
A Human-R exon 2 RP CAGCTGAGTCATCCTCGTCC	This paper	N/A
Mouse -AR region 1 (AR1) FP TCCCTCTGTCCTTTCTCTAACT	This paper	N/A
Mouse -AR region 1 (AR1) RP CCTGAGGATCCATCCCATTTAC	This paper	N/A
Mouse -AR region 2 (AR2) FP GAGGATGTGGAGAAAGAGGAAC	This paper	N/A
Mouse -AR region 2 (AR2) RP GATATATGCCCAGGAGAGGTATTG	This paper	N/A
**qRT-PCR Primers**		
Human-AR FP ATGGTGAGCAGAGTGCCCTATC	Origene	Cat# HP200031
Human-AR RP ATGGTCCCTGGCAGTCTCCAAA	Origene	Cat# HP200031
Human-PSA FP CGCAAGTTCACCCTCAGAAGGT	Origene	Cat# HP227909
Human-PSA RP GACGTGATACCTTGAAGCACACC	Origene	Cat# HP227909
Human-ACTIN FP CACCATTGGCAATGAGCGGTTC	Origene	Cat#HP204660
Human-ACTIN RP AGGTCTTTGCGGATGTCCACGT	Origene	Cat#HP204660
Human-FKBP5 FP GCGAAGGAGAAGACCACGACAT	Origene	Cat#HP207479
Human-FKBP5 RP TAGGCTTCCCTGCCTCTCCAAA	Origene	Cat# HP207479
Human-ANAPC10 FP CGGGAAATTGGGTCACAAGCTG	Origene	Cat# HP211227
Human-ANAPC10 RP GGATGTTCACTAAATGAGGCTGG	Origene	Cat# HP211227
Human-PRKCD FP GCTGACACTTGCCGCAGAGAAT	Origene	Cat# HP209230
Human-PRKCD RP GCCTTTGTCCTGGATGTGGTAC	Origene	Cat# HP209230
Mouse-AR FP CCTTGGATGGAGAACTACTCCG	Origene	Cat# MP200657
Mouse -AR RP TCCGTAGTGACAGCCAGAAGCT	Origene	Cat# MP200657
Mouse-TMPRSS2 FP AAGTCCTCAGGAGCACTGTGCA	Origene	Cat#MP217322
Mouse-TMPRSS2 RP CAGAACCTCCAAAGCAAGACAGC	Origene	Cat#MP217322
Mouse-Actin FP CATTGCTGACAGGATGCAGAAGG	Origene	Cat#MP200232
Mouse-Actin RP TGCTGGAAGGTGGACAGTGAGG	Origene	Cat#MP200232
