# Supplementary material for: SHP2 as a primordial epigenetic enzyme expunges histone H3 pTyr-54 to amend androgen receptor homeostasis
Source: Nat Commun. 2024 Jul 4;15:5629. doi: 10.1038/s41467-024-49978-4 (PMC11224269; doi:10.1038/s41467-024-49978-4)
Supplement: Supplementary file 3 — Description of Additional Supplementary Files [file 41467_2024_49978_MOESM3_ESM.pdf]

Description of Additional Supplementary Files

File Name: Supplementary Data 1
Description: Prostates of (*R*)-**9b** treated mice *Ack1* KO mice and wildtype mice were excised and subjected to ChIP-sequencing with pY54-H3 antibodies

File Name: Supplementary Data 2
Description: pY54-H3 peptide binding proteins isolated from LNCaP cells treated with (*R*)-**9b**

File Name: Supplementary Data 3
Description: Co-occurrence of mutations in ACK1 and SHP2 in multiple malignancies (cBioPortal)
