# Supplementary material for: SHP2 as a primordial epigenetic enzyme expunges histone H3 pTyr-54 to amend androgen receptor homeostasis
Source: Nat Commun. 2024 Jul 4;15:5629. doi: 10.1038/s41467-024-49978-4 (PMC11224269; doi:10.1038/s41467-024-49978-4)
Supplement: Supplementary file 7 — Reporting Summary [file 41467_2024_49978_MOESM7_ESM.pdf]

nature portfolio

Corresponding author(s): Nupam Mahajan

Last updated by author(s): 06/24/2024

Reporting Summary

Nature Portfolio wishes to improve the reproducibility of the work that we publish. This form provides structure for consistency and transparency in reporting. For further information on Nature Portfolio policies, see our Editorial Policies and the Editorial Policy Checklist.

Statistics

For all statistical analyses, confirm that the following items are present in the figure legend, table legend, main text, or Methods section.

n/a	Confirmed	
☐	☒	The exact sample size (*n*) for each experimental group/condition, given as a discrete number and unit of measurement
☐	☒	A statement on whether measurements were taken from distinct samples or whether the same sample was measured repeatedly
☐	☒	The statistical test(s) used AND whether they are one- or two-sided<br>*Only common tests should be described solely by name; describe more complex techniques in the Methods section.*
☐	☒	A description of all covariates tested
☐	☒	A description of any assumptions or corrections, such as tests of normality and adjustment for multiple comparisons
☐	☒	A full description of the statistical parameters including central tendency (e.g. means) or other basic estimates (e.g. regression coefficient) AND variation (e.g. standard deviation) or associated estimates of uncertainty (e.g. confidence intervals)
☐	☒	For null hypothesis testing, the test statistic (e.g. *F*, *t*, *r*) with confidence intervals, effect sizes, degrees of freedom and *P* value noted<br>*Give P values as exact values whenever suitable.*
☒	☐	For Bayesian analysis, information on the choice of priors and Markov chain Monte Carlo settings
☒	☐	For hierarchical and complex designs, identification of the appropriate level for tests and full reporting of outcomes
☐	☒	Estimates of effect sizes (e.g. Cohen's *d*, Pearson's *r*), indicating how they were calculated

Our web collection on statistics for biologists contains articles on many of the points above.

Software and code

Policy information about availability of computer code

Data collection	Prism8: GraphPad https://www.graphpad.com/scientific-software/prism/<br>iBright Analysis Software Thermo Scientific: https://www.thermofisher.com/us/en/home/life-science/protein-biology/protein-assays-analysis/western-blotting/detect-proteins-western-blot/western-blot-imaging-analysis/ibright-western-blot-imaging-systems/ibright-analysissoftware-connectivity.html<br>StepOne and StepOnePlus Software v2.3 Applied Biosystems: https://www.thermofisher.com/us/en/home/technical-resources/softwaredownloads/<br>StepOne-and-StepOnePlus-Real-Time-PCR-System.html<br>EVOS M5000 Imaging System Software Invitrogen: https://www.thermofisher.com/us/en/home/technical-resources/software-downloads/evos-m5000-imaging-system-software-download.html<br>Confocal Microscopy: Zen blue software https://www.zeiss.com/microscopy/en/products/software/zeiss-zen.html<br>Capillary liquid chromatography interfaced to a mass spectrometer (nano-LC-MS/MS).
Data analysis	ImageJ National Institutes of Health: https://imagej.nih.gov/ij/<br>Adobe Photoshop Version 24.x Adobe: https://www.adobe.com/products/photoshop.html<br>Adobe Illustrator Version 26.4 Adobe: https://www.adobe.com/products/illustrator.html<br>Chip-seq Analysis Software R/Bioconductor package and MACS peak-finding software<br>WashU Epigenome Browser, GSEA analysis<br><br>Mass Spectrometry<br>Cells were lysed in denaturing buffer containing 8 M urea, 20 mM HEPES (pH 8), 1 mM sodium orthovanadate, 2.5 mM sodium pyrophosphate and 1 mM β-glycerophosphate. Bradford assays determined the protein concentration for each sample. Protein disulfides were reduced with

4.5 mM DTT at 60 °C for 30 minutes and then cysteines were alkylated with 10 mM iodoacetamide for 20 minutes in the dark at room temperature. Trypsin digestion was carried out at room temperature overnight with enzyme to substrate ratio of 1:20, and tryptic peptides were acidified with aqueous 1% trifluoroacetic acid (TFA) and desalted with C18 Sep-Pak cartridges according to the manufacturer's procedure. Following lyophilization, peptide pellets were re-dissolved in immunoaffinity purification (IAP) buffer containing 50 mM MOPS pH 7.2, 10 mM sodium phosphate and 50 mM NaCl. Phosphotyrosine-containing peptides (pY) were immunoprecipitated with p-Tyr-1000 beads (Cell Signaling Technology #8803S).

A nanoflow ultra high-performance liquid chromatograph and nanoelectrospray orbitrap mass spectrometer (RSLCnano and Q Exactive plus, Thermo) were used for LC-MS/MS. The sample was loaded onto a pre-column (C18 PepMap100, 2 cm length x 100 µm ID packed with C18 reversed-phase resin, 5 µm particle size, 100 Å pore size) and washed for 8 minutes with aqueous 2% acetonitrile and 0.1% formic acid. Trapped peptides were eluted onto the analytical column, (C18 PepMap100, 25 cm length x 75 µm ID, 2 µm particle size, 100 Å pore size, Thermo). A 120-minute gradient was programmed as: 95% solvent A (aqueous 2% acetonitrile + 0.1% formic acid) for 8 minutes, solvent B (aqueous 90% acetonitrile + 0.1% formic acid) from 5% to 38.5% in 90 minutes, then solvent B from 50% to 90% B in 7 minutes and held at 90% for 5 minutes, followed by solvent B from 90% to 5% in 1 minute and re-equilibration for 10 minutes using a flow rate of 300 nl/min. Spray voltage was 1900 V. Capillary temperature was 275 °C. S lens RF level was set at 40. Top 16 tandem mass spectra were collected in a data-dependent manner. The resolution for MS and MS/MS were set at 70,000 and 17,500 respectively. Dynamic exclusion was 15 seconds for previously sampled peaks.
Data Analysis: MaxQuant (version 1.2.2.5) was used to identify peptides using the UniProt human database and quantify the MS1 precursor intensities. Up to 2 missed trypsin cleavages were allowed. The mass tolerance was 20 ppm first search and 4.5 ppm main search. Carbamidomethyl cysteine was set as fixed modification. Phosphorylation on Serine/Threonine/Tyrosine and Methionine oxidation were set as variable modifications. Both peptide spectral match (PSM) and protein false discovery rate (FDR) were set at 0.05. Match between runs feature was activated to carry identifications across samples. For data upload to PRIDE/ProteomeXchange, similar database searches were performed with Mascot (www.matrixscience.com) in Proteome Discoverer (Thermo).

Quantitative RT-PCR
Cells under various experimental conditions were for RNA isolation and cDNA preparation as described earlier48. All RT reactions were done at the same time so that the same reactions could be used for all gene studies. For the construction of standard curves, serial dilutions of pooled sample RNA were used (50, 10, 2, 0.4, 0.08, and 0.016 ng) per reverse transcriptase reaction. One "no RNA" control and one "no Reverse Transcriptase" control were included for the standard curve. Three reactions were performed for each sample: 10 ng, 0.8 ng, and a NoRT (10 ng) control. Real-time quantitative PCR analyses were performed using the ABI PRISM 7900HT Sequence Detection System (Applied Biosystems). All standards, the no template control (H_2O), the No RNA control, the no Reverse Transcriptase control, and the no amplification control (Bluescript plasmid) were tested in six wells per gene (2 wells/plate x 3 plates/gene). All samples were tested in triplicate wells each for the 10 ng and 0.8 ng concentrations. PCR was carried out with SYBR® Premix Ex TaqTM II TB green premix (TaKaRa, RR82LR) using 2 µl of cDNA and the primers in a 20 µl final reaction mixture. After 2 min incubation at 50°C, reaction was activated by 10 min incubation at 95°C, followed by 40 PCR cycles consisting of 15 s of denaturation at 95°C and hybridization of primers for 1 min at 55°C. Dissociation curves were generated for each plate to verify the integrity of the primers. Data were analyzed using StepOne and StepOnePlus software version 2.3 and exported into an Excel spreadsheet. The actin or GAPDH data were used for normalizing the gene values; i.e., ng gene/ng Actin or GAPDH per well.

Computational analysis of ChIP-Seq data: Sequence analysis
The 75-nt paired end sequence reads were mapped to the genome using the BWA-MEM algorithm. Alignment information for each read was stored in the output file *.bam. Only reads that mapped uniquely with proper pairing were used in the subsequent analysis.

Determination of fragment density
Because the 5′ ends of the sequence reads represent the end of the ChIP or immunoprecipitation fragments, the reads were extended in silico (using MAC2) at their 3′ ends to a length of 173-244 bp, based on the fragment length calculated from the read pairs.

Peak finding
Peak regions were called using the MACS2 software with the following options -f BAMPE -SPMR -q 0.01 -broad. The "BAMPE" option was used for calculating fragment lengths from the paired end reads, "SPMR" for normalizing read depths to number of fragments per million reads, "broad" for compositing broad regions from nearby peak regions, and the qvalue (FDR) cutoff was set to 0.01.

Motif analysis
Using the HOMER (v4.7, 8-25-2014) program (http://homer.salk.edu/homer/) we found 13 de novo motifs in LNCaP cells treated with (R)-9b, pY54-H3 ChIP data, which are shown in Figure S6a. We also identified 3 known motifs, in Ack1 KO prostates, shown in Figure S6b.

Tissue Microarray (TMA) Analysis
The prostate TMAs were obtained from US Biomax (PR807c: 80 cases) for our study for which we are exempt from IRB approval, as no personal information about patients is sought. The tissue array slides (including positive and negative controls) were stained for the antibodies. The slides were dewaxed by heating at 65° Celsius for 60 min, washed two times, 15 min each, with xylene. Tissues were rehydrated by two series of 10 min washes in 100%, 95%, and 70% ethanol and distilled water. Antigen retrieval was performed by heating the samples at 950C for 25 min in 10 mmol/L sodium citrate (pH 6.0). The slides were cooled in PBS for 30 min, with 10 min changes of PBS and permeabilized using 0.2% Triton-X100 in PBS for 10 min. Slides were washed with PBS+0.2% Tween-20 for 10 min. After blocking with universal blocking serum (DAKO Diagnostic, Mississauga, Ontario, Canada) for 30 min, the samples were incubated with rabbit monoclonal pY580-SHP2 (1:300 dilution) and rabbit polyclonal pY54-H3 antibody (1:300 dilution) at 4[illegible]C overnight. The sections were incubated with biotin-labeled secondary and streptavidin-peroxidase for 30 min each (DAKO Diagnostic). The samples were developed with 3,39-diaminobenzidine substrate (Vector Laboratories, Burlington, Ontario, Canada) and counterstained with hematoxylin. Following standard procedures, the slides were dehydrated and sealed with cover slips. The pY-SHP2 and pY54-H3 staining were examined in a blinded fashion by pathologist (C.W.). The positive reactions were scored into four grades according to the intensity of staining: 0, 1+, 2+ and 3+.

For manuscripts utilizing custom algorithms or software that are central to the research but not yet described in published literature, software must be made available to editors and reviewers. We strongly encourage code deposition in a community repository (e.g. GitHub). See the Nature Portfolio guidelines for submitting code & software for further information.

Data

Policy information about availability of data

All manuscripts must include a data availability statement. This statement should provide the following information, where applicable:

- Accession codes, unique identifiers, or web links for publicly available datasets
- A description of any restrictions on data availability
- For clinical datasets or third party data, please ensure that the statement adheres to our policy

The mass spec data is submitted in ProteomeXchange (PRIDE) with the accession number PXD037546.
ChIP-sequencing datasets generated in this study have been deposited in GEO database with the GEO accession number: GSE214870.
The remaining data are available within the Article, Supplementary Information or Source Data file. Source data are provided with this paper.
Any additional datasets generated during and/or analyzed during the current study are available from the corresponding author on reasonable request.

Research involving human participants, their data, or biological material

Policy information about studies with human participants or human data. See also policy information about sex, gender (identity/presentation), and sexual orientation and race, ethnicity and racism.

Reporting on sex and gender	Pediatric males were included for the study
Reporting on race, ethnicity, or other socially relevant groupings	N/A
Population characteristics	N/A
Recruitment	N/A
Ethics oversight	The stem cells were derived from fibroblasts of the NSML patient and the healthy individual with informed consent approved by the local ethics committee of the medical faculty at the Justus-Liebig-University Giessen, Germany.

Note that full information on the approval of the study protocol must also be provided in the manuscript.

Field-specific reporting

Please select the one below that is the best fit for your research. If you are not sure, read the appropriate sections before making your selection.

☒ Life sciences ☐ Behavioural & social sciences ☐ Ecological, evolutionary & environmental sciences

For a reference copy of the document with all sections, see nature.com/documents/nr-reporting-summary-flat.pdf

Life sciences study design

All studies must disclose on these points even when the disclosure is negative.

Sample size	The number of samples for each assay was indicated in each figure legend. For in vitro assays, the sample sizes (at least three biological replicates) were chosen with these assays yielding statistically significant difference between experimental positive and negative controls and on similar sample sizes. Results are representatives of at least three biological replicates and at least two independent experiments. For in vivo assays, at least 3 mice were chosen for each condition, this sample size was determined by using power calculation for a t-test difference between two or three independent means based on a normally distributed population with equal variance.
Data exclusions	No data was excluded from the analysis.
Replication	Each experiment was repeated atleast twice, thrice in most cases for reproducibility. All primers were tested by gel electrophoresis and melt curves evaluated before using them in the quantitative assays for reproducibility.<br>Western blots were repeated atleast twice to ensure reproducibility and captured digitally using iBright imager. All data is available for review. Replicated experiments were successful and support conclusions drawn in this report.
Randomization	Animals were randomly assigned to two or more groups prior to the injection of cells or drug. The rest of the experiments were not randomized, but independent replicates were often performed in different formats, as mitigation measures to cancel out experimental bias.
Blinding	Investigators were not blinded for any of the experiments, including to the animal assignments for tumor formation studies and molecular analysis as treatment conditions were evident from the data. All tissue sections were independently evaluated by pathologist for presence of antibody validation studies and for tumor versus normal determination.

Reporting for specific materials, systems and methods

We require information from authors about some types of materials, experimental systems and methods used in many studies. Here, indicate whether each material, system or method listed is relevant to your study. If you are not sure if a list item applies to your research, read the appropriate section before selecting a response.

Materials & experimental systems

n/a	Involved in the study	
☐	☒	Antibodies
☐	☒	Eukaryotic cell lines
☒	☐	Palaeontology and archaeology
☐	☒	Animals and other organisms
☒	☐	Clinical data
☒	☐	Dual use research of concern
☒	☐	Plants

Methods

n/a	Involved in the study	
☐	☒	ChIP-seq
☐	☒	Flow cytometry
☒	☐	MRI-based neuroimaging

Antibodies

Antibodies used

Anti-FLAG Cell Signaling Tech Cat#14793S Clone D6W5B Lot#5
Anti-HA Cell Signaling Technology, Cat# 2367S Clone 6E2 Lot#5
Anti-MYC Cell Signaling Tech Cat#2276S Clone 9B11 Lot#24
Anti-pTyr Santa Cruz Bio Cat#sc-508 Clone PY20 Lot#D0319
Anti-Actin Sigma Cat#A2228 Clone AC-74
Anti-pY-SHP2 Cell Signaling Technology, Cat#5431S, Clone D66F10
Anti-pY54-H3 21st Century, this paper, 1:1000
Anti-H3 Cell Signaling Technology, Cat# 14269, Clone 1B1B2
Anti-SHP2 Cell Signaling Technology, Cat#3397S, Clone D50F2
Anti-ACK1 Santa Cruz Biotechnology, Cat# sc-28336, Clone A11
Anti-p-Y-284-TNK2/ACK1 Millipore Sigma, Cat#09-142
Anti-His Cell Signaling Technology, Cat# 2366, Clone 27E8
Anti-NCoR1 Cell Signaling Technology, Cat# 5948S
Anti-SMRT (NCoR2) Cell Signaling Technology, Cat# 62370
Anti-IR Cell Signaling Technology, Cat# 3025, Clone 4B8
Anti-HER4 Cell Signaling Technology, Cat# 4795, Clone 111B2
Anti-SRC Cell Signaling Technology, Cat# 2108S
Anti-HSP90 Cell Signaling Technology, Cat# 4874, 1:1000
Anti-PTPN1 Santa Cruz Biotechnology, Cat# sc-133259, Clone D-4
Anti-PTPN2 Santa Cruz Biotechnology, Cat# sc-376864
anti-SHP1 Santa Cruz Biotechnology, Cat# sc-7289, Clone D11
LIVE/DEAD™ Fixable Aqua Dead Cell Stain Kit Invitrogen Cat#L34957
Goat anti-Rabbit IgG (H+L) Highly Cross-Adsorbed Secondary Antibody, Alexa Fluor™ 488

Validation

The effectiveness of the antibodies was confirmed by performing immunoblotting experiments using the manufacturer's data associated with antibody, and their authentication data.
Anti-FLAG (https://www.cellsignal.com/products/primary-antibodies/dykddddk-tag-d6w5b-rabbit-mab-binds-to-same-epitope-assigma-
s-anti-flag-m2-antibody/14793)
Anti-HA (https://www.cellsignal.com/products/primary-antibodies/ha-tag-6e2-mouse-mab/2367)
Anti-MYC (https://www.cellsignal.com/products/primary-antibodies/myc-tag-9b11-mouse-mab/2276)
Anti-pTyr (https://www.scbt.com/p/p-tyr-antibody-py20)
Anti-Actin (https://www.sigmaaldrich.com/US/en/product/sigma/a2228)
Fixable Aqua Dead Cell stain (https://www.thermofisher.com/order/catalog/product/L34957)
Anti-pY-SHP2 (https://www.cellsignal.com/products/primary-antibodies/phospho-shp-2-tyr580-antibody/3703)
Anti-H3 (https://www.cellsignal.com/products/primary-antibodies/histone-h3-1b1b2-mouse-mab/14269?_requestid=3649052)
Anti-SHP2 (https://www.cellsignal.com/products/primary-antibodies/shp-2-d50f2-rabbit-mab/3397)
Anti-ACK1 (https://www.scbt.com/p/ack-antibody-a-11)
Anti-pY284-TNK2/ACK1 (https://www.emdmillipore.com/US/en/product/Anti-phospho-ACK1-Tyr284-Antibody,MM_NF-09-142)
Anti-His (https://www.cellsignal.com/products/primary-antibodies/his-tag-27e8-mouse-mab/2366)
Anti-NCoR1 (https://www.cellsignal.com/products/primary-antibodies/ncor1-antibody/5948)
Anti-SMRT (NCoR2) (https://www.cellsignal.com/products/primary-antibodies/smrt-d8d2l-rabbit-mab/62370)
Anti-IR (https://www.cellsignal.com/products/primary-antibodies/insulin-receptor-b-4b8-rabbit-mab/3025)
Anti-HER4 (https://www.cellsignal.com/products/primary-antibodies/her4-erbb4-111b2-rabbit-mab/4795)
Anti-SRC (https://www.cellsignal.com/products/primary-antibodies/src-antibody/2108)
Anti-HSP90 (https://www.cellsignal.com/products/primary-antibodies/hsp90-antibody/4874)
Anti-PTPN1 (https://www.scbt.com/p/ptp1b-antibody-d-4)
Anti-PTPN2 (https://www.scbt.com/p/tc-ptp-antibody-e-11)
anti-SHP1 (https://www.scbt.com/p/sh-ptp1-antibody-d-11)
Fixable Aqua Dead Cell Stain (https://www.thermofisher.com/order/catalog/product/L34957)

Goat anti-Rabbit IgG (H+L) Highly Cross-Adsorbed Secondary Antibody, Alexa Fluor™ 488 (https://www.thermofisher.com/antibody/product/Goat-anti-Rabbit-IgG-H-L-Highly-Cross-Adsorbed-Secondary-Antibody-Polyclonal/A-11034)

Eukaryotic cell lines

Policy information about cell lines and Sex and Gender in Research

Cell line source(s)	RWPE-1, HEK293T, LNCaP, VCaP, PC3, LNCaP-C4-2B and DU145 cells were obtained from ATCC.
Authentication	Identities of all cell lines were confirmed by Short Tandem Repeat (STR) Profiling.
Mycoplasma contamination	All cultures were tested for mycoplasma contamination every 2 months using the PCR Mycoplasma Test Kit I/C (PromoKine). Mycoplasma testing was negative.
Commonly misidentified lines (See ICLAC register)	None

Animals and other research organisms

Policy information about studies involving animals; ARRIVE guidelines recommended for reporting animal research, and Sex and Gender in Research

Laboratory animals	5-6 weeks old male SCID mice (CB17/Icr-Prkdcscid/IcrIcoCrl Strain code: 236) were purchased from Charles River Laboratories, USA. Number mice used per experiments are described in manuscript.
Wild animals	No wild animals were used in the study.
Reporting on sex	Male mice were used in this study.
Field-collected samples	No wild animals were used in the study.
Ethics oversight	All animal studies were performed under approved Institutional Animal Care and Use Committee protocols at Washington University in St. Louis; Approved IACUC protocol # 20180259

Note that full information on the approval of the study protocol must also be provided in the manuscript.

Plants

Seed stocks	N/A
Novel plant genotypes	N/A
Authentication	N/A

ChIP-seq

Data deposition

☒ Confirm that both raw and final processed data have been deposited in a public database such as GEO.

☒ Confirm that you have deposited or provided access to graph files (e.g. BED files) for the called peaks.

Data access links *May remain private before publication.*	ChIP-Sequencing data is submitted to the GEO, accession GSE214870:<br>Go to https://www.ncbi.nlm.nih.gov/geo/query/acc.cgi?acc=GSE214870
Files in database submission	LNCaP Ctrl<br>LNCaP 9b<br>LNCaP SHP2i<br><br>Mice Prostate Input<br>Mice Prostate R-9b treated<br>Mice prostate WT<br>Mice Prostate SHP2 inhibitor treated<br>Mice Prostate KO
Genome browser session (e.g. UCSC)	No longer applicable

Methodology

Replicates	Each sample was run in duplicate

Sequencing depth	Ten nanograms of immunoprecipitated DNA from LNCaP cell line was used to generate sequencing libraries using the Kapa Hyper Prep Kit (Roche Sequencing Solutions Inc., Pleasanton, CA). The size and quality of the library was evaluated using the Agilent BioAnalyzer (Agilent Technologies, Inc., Santa Clara, CA), and the library was quantitated with the Kapa Library Quantification Kit. Each enriched DNA library was then sequenced on an Illumina NextSeq 500 sequencer to generate 40-50 million 75-base paired-end reads (Illumina, Inc., San Diego, CA).
Antibodies	pY54-H3 antibodies were custom synthesized by 21st Century Biochem, MA.
Peak calling parameters	Peak regions were called using the MACS2 software with the following options-f BAMPE-SPMR-q 0.01-broad. he "BAMPE" option was used for calculating fragment lengths from the paired end reads, "SPMR" for normalizing read depths to number of fragments per million reads, "broad" for compositing broad regions from nearby peak regions. The vehicle treated sample was used as the control.
Data quality	The q value (FDR) cutoff was set to 0.01. The peaks were primarily used for further validation using qPCR.
Software	The raw sequence data were aligned using BowTie 2 and binding sites were identified using the MACS peak-finding software

Flow Cytometry

Plots

Confirm that:

☒ The axis labels state the marker and fluorochrome used (e.g. CD4-FITC).

☒ The axis scales are clearly visible. Include numbers along axes only for bottom left plot of group (a 'group' is an analysis of identical markers).

☒ All plots are contour plots with outliers or pseudocolor plots.

☒ A numerical value for number of cells or percentage (with statistics) is provided.

Methodology

Sample preparation	Increase in pY54-H3 upon ACK1 knock down was assessed by flow cytometry. Under sterile conditions, femur, heart, liver, and prostate were harvested from naïve WT and Ack1 KO mice. Single cells were made, and RBCs were lysed using RBC lysis buffer. 1x106 cells were incubated with Live/Dead Aqua (1:800) and fixed. Cells were then permeabilized and incubated with pY54-H3 (1:300). Flow cytometry was performed after intracellular staining with anti-rabbit Alexa Fluor® 488 antibody (Invitrogen, Cat# A11034, 1:700).
Instrument	Samples were analyzed using BD FACSCanto II (BD Biosciences)
Software	Post-acquisition analysis was done using FlowJo software (Tree Star Inc)
Cell population abundance	The isolated single cells from the organs were 80-90% live to check the intensity of pY54-H3 expression levels in their nucleus.
Gating strategy	The first gate of forward and side scatter (FSC and SSC) was created. Following this, the live cells were gated and intensity of pY54-H3 staining in these cells was plotted as histogram.

☒ Tick this box to confirm that a figure exemplifying the gating strategy is provided in the Supplementary Information.
