# Supplementary material for: SHP2 as a primordial epigenetic enzyme expunges histone H3 pTyr-54 to amend androgen receptor homeostasis
Source: Nat Commun. 2024 Jul 4;15:5629. doi: 10.1038/s41467-024-49978-4 (PMC11224269; doi:10.1038/s41467-024-49978-4)
Supplement: Supplementary file 8 — Source Data [file 41467_2024_49978_MOESM8_ESM.zip › Source data/422664_3_data_set_9024582_sd2t7l.pptx]

## Slide 1
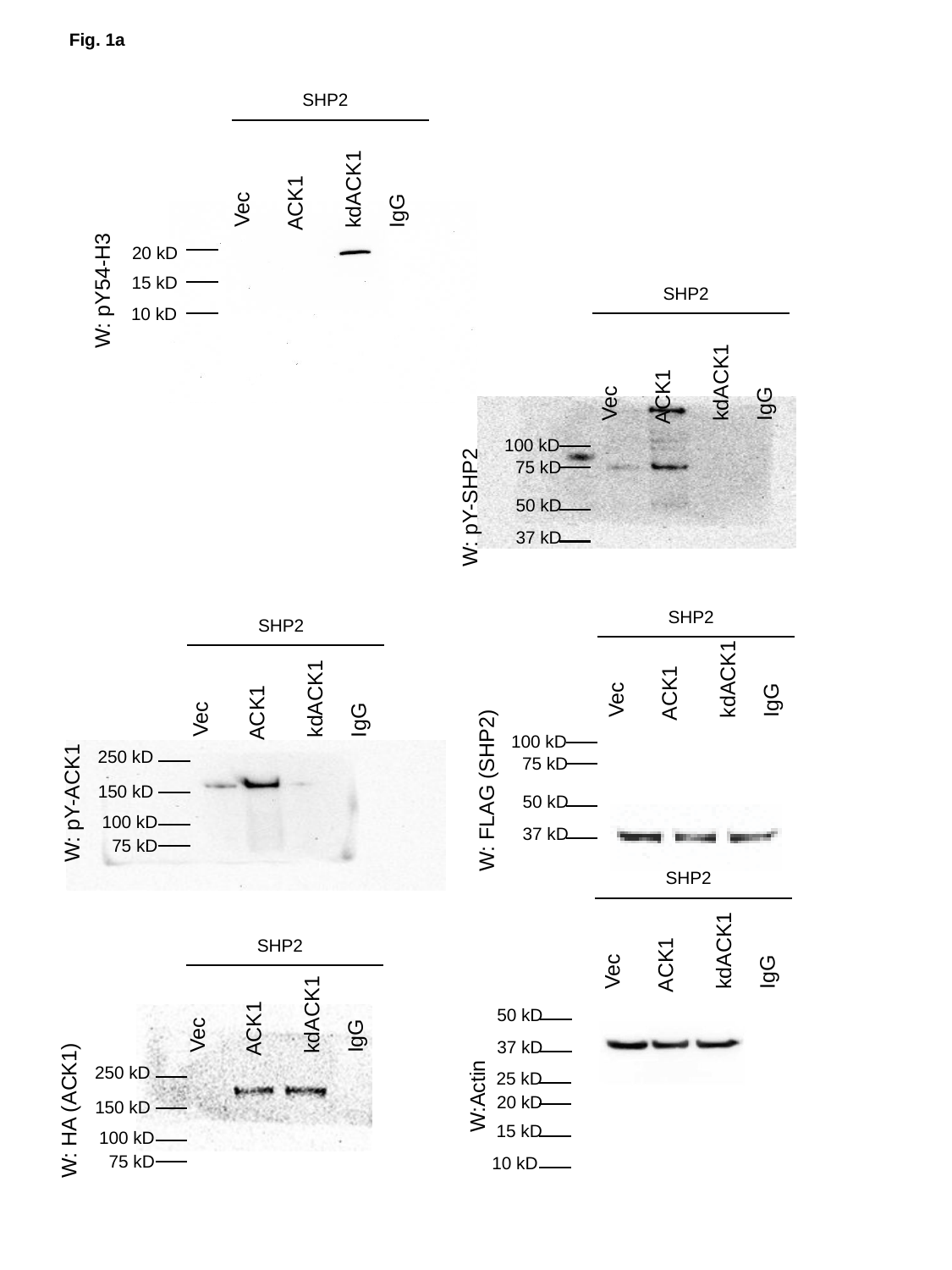

Fig. 1a
SHP2
kdACK1
ACK1
IgG
Vec
 20 kD
W: pY54-H3
 15 kD
SHP2
 10 kD
kdACK1
ACK1
IgG
Vec
 100 kD
 75 kD
W: pY-SHP2
 50 kD
 37 kD
SHP2
SHP2
kdACK1
ACK1
IgG
kdACK1
Vec
ACK1
IgG
Vec
 100 kD
250 kD
 75 kD
W: pY-ACK1
W: FLAG (SHP2)
150 kD
 50 kD
 100 kD
 37 kD
 75 kD
SHP2
SHP2
kdACK1
ACK1
IgG
Vec
kdACK1
 50 kD
ACK1
IgG
Vec
 37 kD
W:Actin
250 kD
 25 kD
W: HA (ACK1)
 20 kD
150 kD
 15 kD
 100 kD
 75 kD
 10 kD

## Slide 2
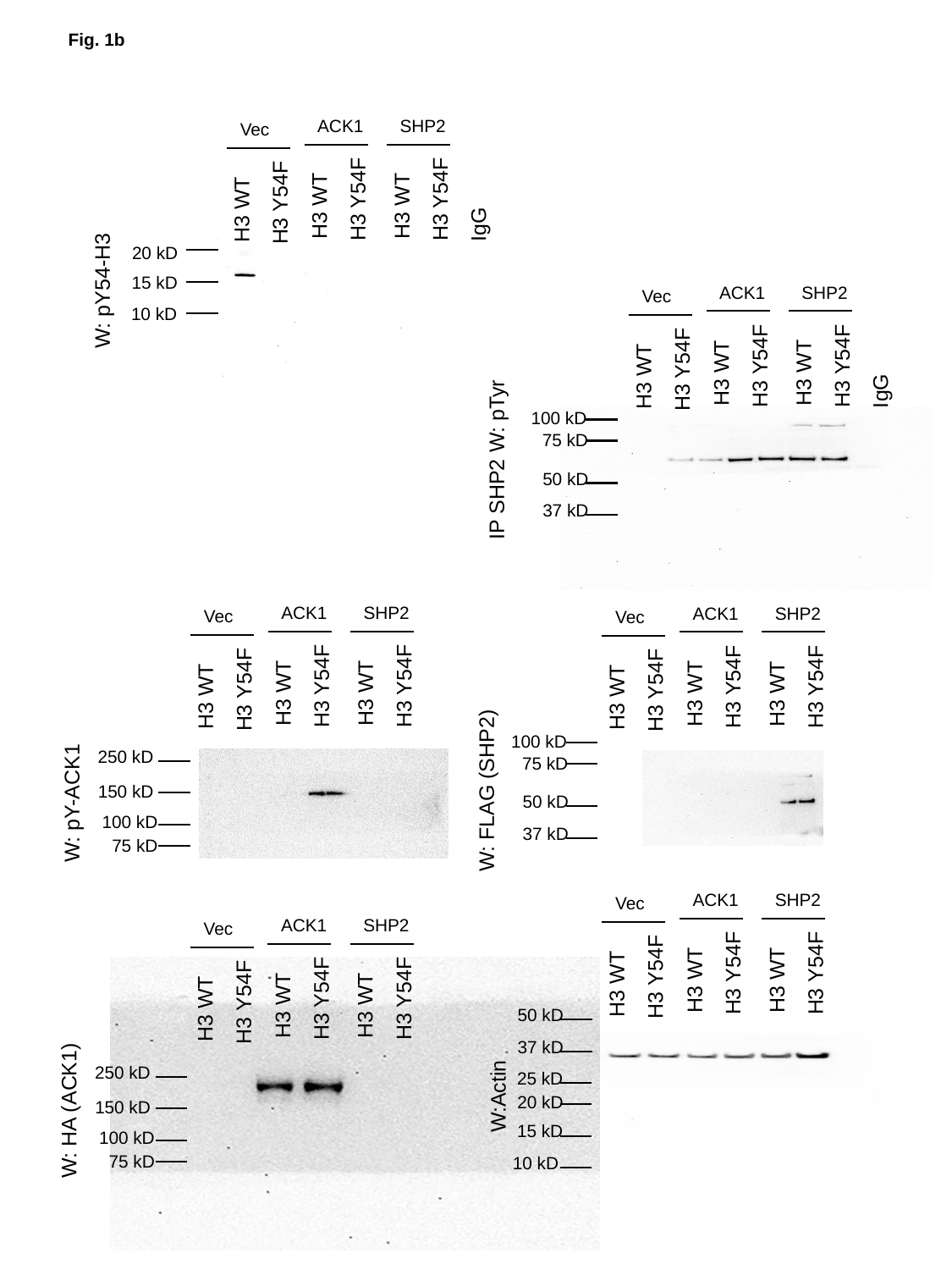

Fig. 1b
SHP2
ACK1
Vec
H3 Y54F
H3 Y54F
H3 Y54F
H3 WT
H3 WT
H3 WT
IgG
 20 kD
W: pY54-H3
 15 kD
SHP2
ACK1
Vec
 10 kD
H3 Y54F
H3 Y54F
H3 Y54F
H3 WT
H3 WT
H3 WT
IgG
 100 kD
IP SHP2 W: pTyr
 75 kD
 50 kD
 37 kD
SHP2
ACK1
SHP2
ACK1
Vec
Vec
H3 Y54F
H3 Y54F
H3 Y54F
H3 Y54F
H3 Y54F
H3 Y54F
H3 WT
H3 WT
H3 WT
H3 WT
H3 WT
H3 WT
 100 kD
250 kD
 75 kD
W: pY-ACK1
W: FLAG (SHP2)
150 kD
 50 kD
 100 kD
 37 kD
 75 kD
SHP2
ACK1
Vec
SHP2
ACK1
Vec
H3 Y54F
H3 Y54F
H3 Y54F
H3 WT
H3 WT
H3 WT
H3 Y54F
H3 Y54F
H3 Y54F
H3 WT
H3 WT
H3 WT
 50 kD
 37 kD
W:Actin
250 kD
 25 kD
W: HA (ACK1)
 20 kD
150 kD
 15 kD
 100 kD
 75 kD
 10 kD

## Slide 3
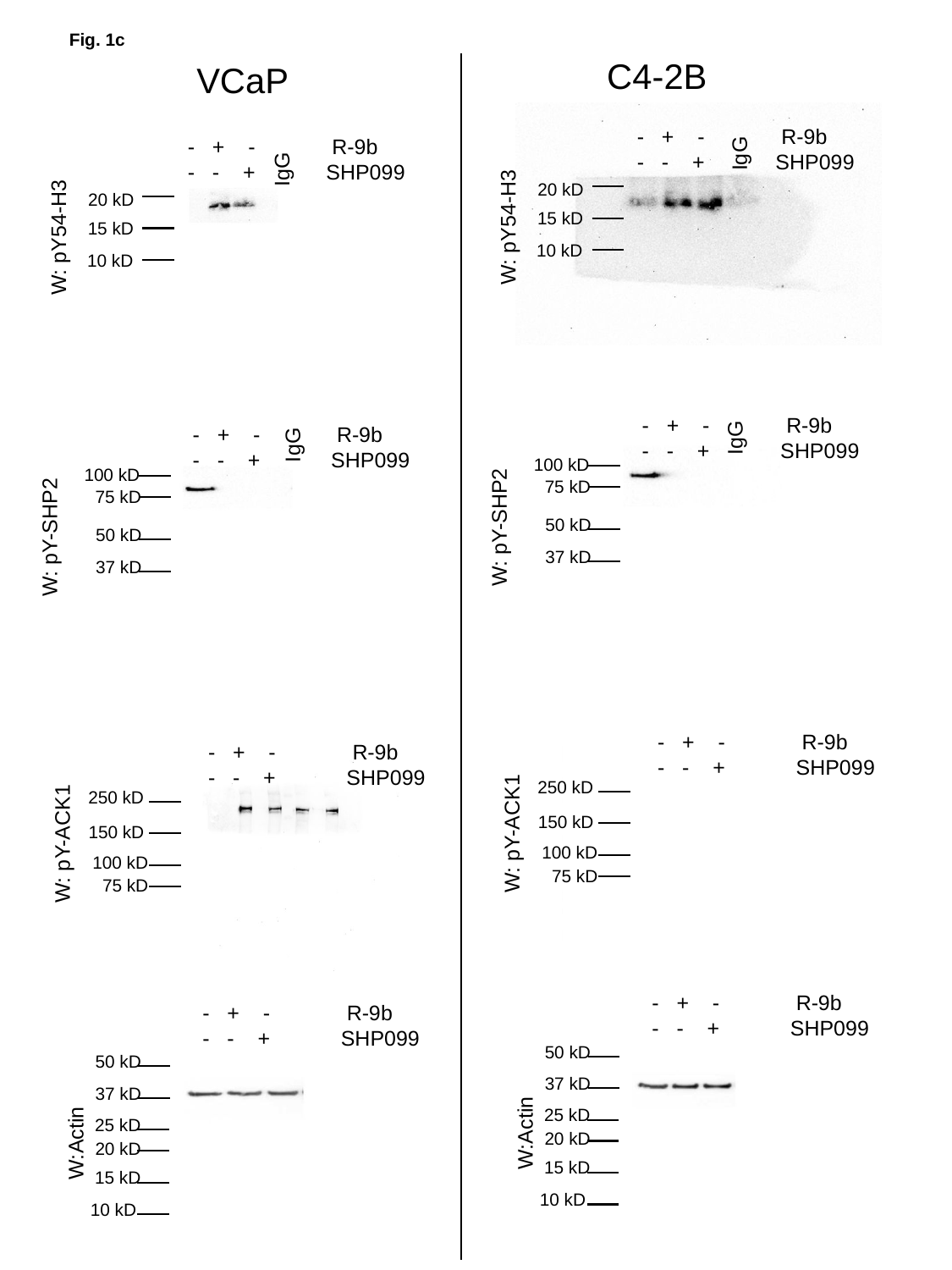

Fig. 1c
C4-2B
VCaP
 - + - R-9b
 - - + SHP099
IgG
 - + - R-9b
 - - + SHP099
IgG
 20 kD
 20 kD
W: pY54-H3
 15 kD
W: pY54-H3
 15 kD
 10 kD
 10 kD
 - + - R-9b
 - - + SHP099
IgG
IgG
 - + - R-9b
 - - + SHP099
 100 kD
 100 kD
 75 kD
 75 kD
W: pY-SHP2
 50 kD
W: pY-SHP2
 50 kD
 37 kD
 37 kD
 - + - R-9b
 - - + SHP099
 - + - R-9b
 - - + SHP099
250 kD
250 kD
W: pY-ACK1
W: pY-ACK1
150 kD
150 kD
 100 kD
 100 kD
 75 kD
 75 kD
 - + - R-9b
 - - + SHP099
 - + - R-9b
 - - + SHP099
 50 kD
 50 kD
 37 kD
 37 kD
W:Actin
W:Actin
 25 kD
 25 kD
 20 kD
 20 kD
 15 kD
 15 kD
 10 kD
 10 kD

## Slide 4
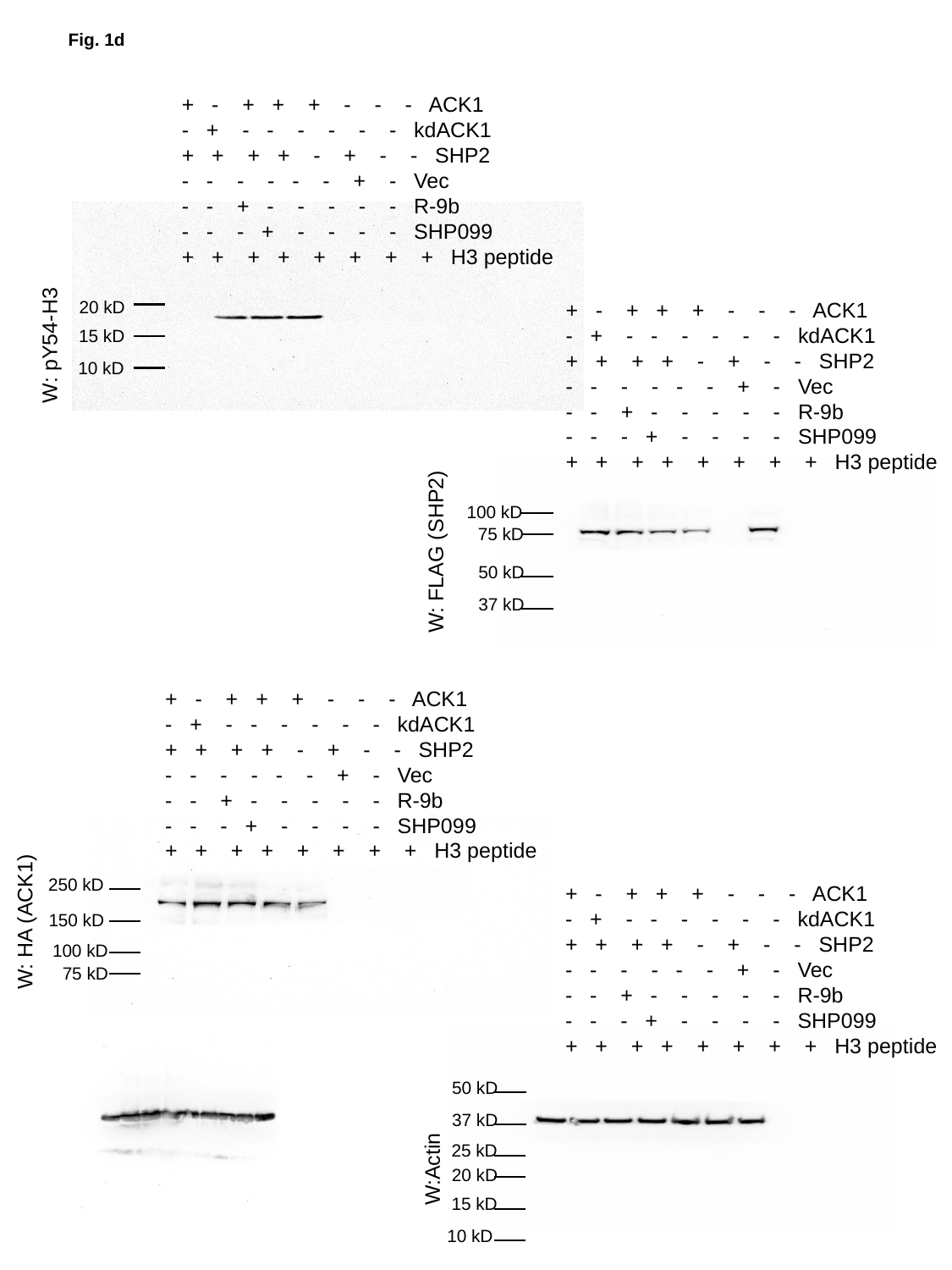

Fig. 1d
+ - + + + - - - ACK1
- + - - - - - - kdACK1
+ + + + - + - - SHP2
- - - - - - + - Vec
- - + - - - - - R-9b
- - - + - - - - SHP099
+ + + + + + + + H3 peptide
 20 kD
+ - + + + - - - ACK1
- + - - - - - - kdACK1
+ + + + - + - - SHP2
- - - - - - + - Vec
- - + - - - - - R-9b
- - - + - - - - SHP099
+ + + + + + + + H3 peptide
W: pY54-H3
 15 kD
 10 kD
 100 kD
 75 kD
W: FLAG (SHP2)
 50 kD
 37 kD
+ - + + + - - - ACK1
- + - - - - - - kdACK1
+ + + + - + - - SHP2
- - - - - - + - Vec
- - + - - - - - R-9b
- - - + - - - - SHP099
+ + + + + + + + H3 peptide
250 kD
+ - + + + - - - ACK1
- + - - - - - - kdACK1
+ + + + - + - - SHP2
- - - - - - + - Vec
- - + - - - - - R-9b
- - - + - - - - SHP099
+ + + + + + + + H3 peptide
W: HA (ACK1)
150 kD
 100 kD
 75 kD
 50 kD
 37 kD
W:Actin
 25 kD
 20 kD
 15 kD
 10 kD

## Slide 5
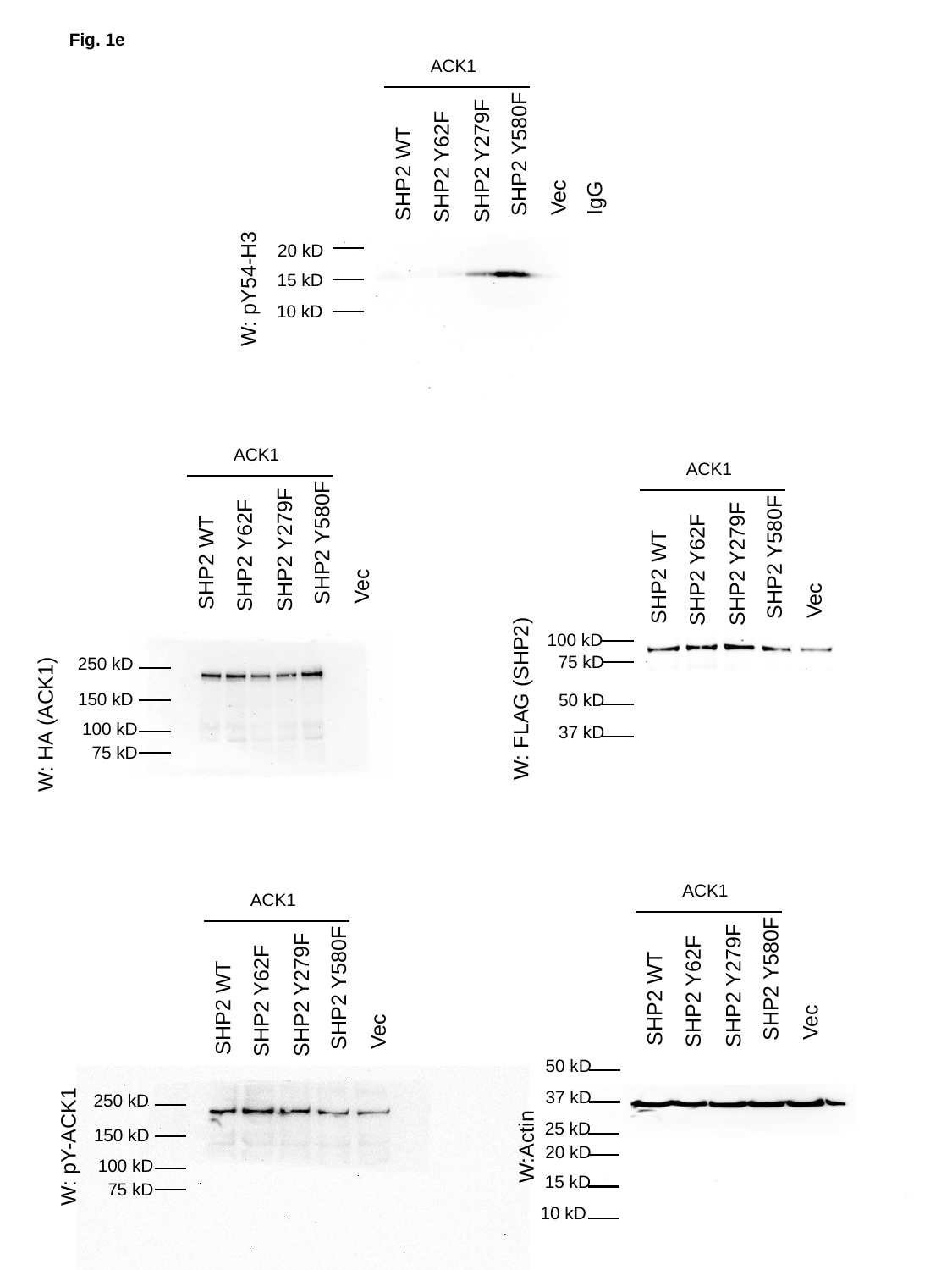

Fig. 1e
ACK1
SHP2 Y580F
SHP2 Y279F
SHP2 Y62F
SHP2 WT
Vec
IgG
 20 kD
W: pY54-H3
 15 kD
 10 kD
ACK1
ACK1
SHP2 Y580F
SHP2 Y279F
SHP2 Y62F
SHP2 Y580F
SHP2 WT
SHP2 Y279F
SHP2 Y62F
Vec
SHP2 WT
Vec
 100 kD
 75 kD
250 kD
W: FLAG (SHP2)
150 kD
 50 kD
W: HA (ACK1)
 100 kD
 37 kD
 75 kD
ACK1
ACK1
SHP2 Y580F
SHP2 Y279F
SHP2 Y580F
SHP2 Y62F
SHP2 Y279F
SHP2 WT
SHP2 Y62F
SHP2 WT
Vec
Vec
 50 kD
 37 kD
250 kD
W: pY-ACK1
W:Actin
 25 kD
150 kD
 20 kD
 100 kD
 15 kD
 75 kD
 10 kD

## Slide 6
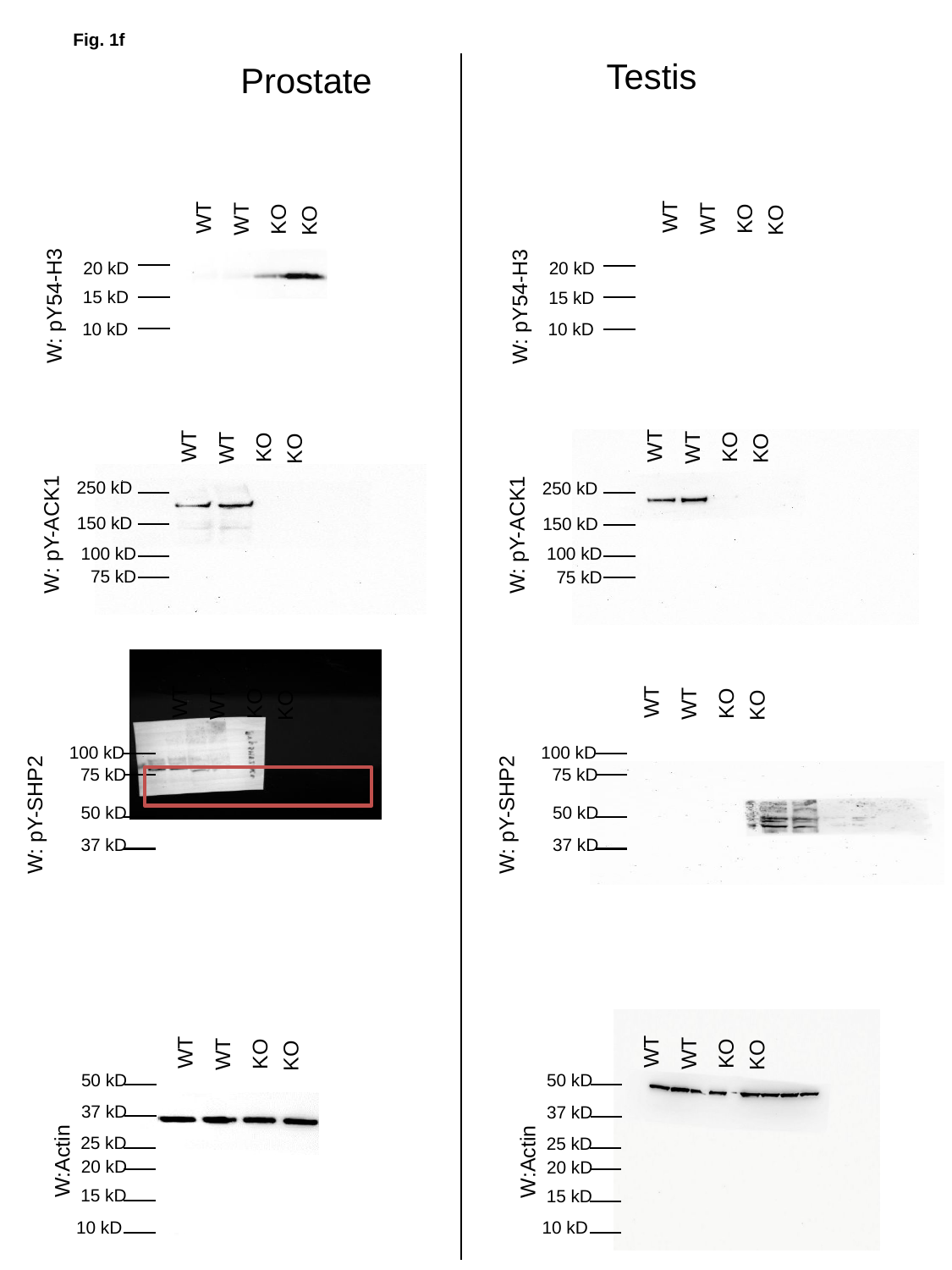

Fig. 1f
Testis
Prostate
KO
KO
WT
WT
KO
KO
WT
WT
 20 kD
 20 kD
W: pY54-H3
W: pY54-H3
 15 kD
 15 kD
 10 kD
 10 kD
KO
KO
WT
WT
KO
KO
WT
WT
250 kD
250 kD
W: pY-ACK1
W: pY-ACK1
150 kD
150 kD
 100 kD
 100 kD
 75 kD
 75 kD
KO
KO
WT
WT
KO
KO
WT
WT
 100 kD
 100 kD
 75 kD
 75 kD
W: pY-SHP2
W: pY-SHP2
 50 kD
 50 kD
 37 kD
 37 kD
KO
KO
WT
WT
KO
KO
WT
WT
 50 kD
 50 kD
 37 kD
 37 kD
W:Actin
W:Actin
 25 kD
 25 kD
 20 kD
 20 kD
 15 kD
 15 kD
 10 kD
 10 kD

## Slide 7
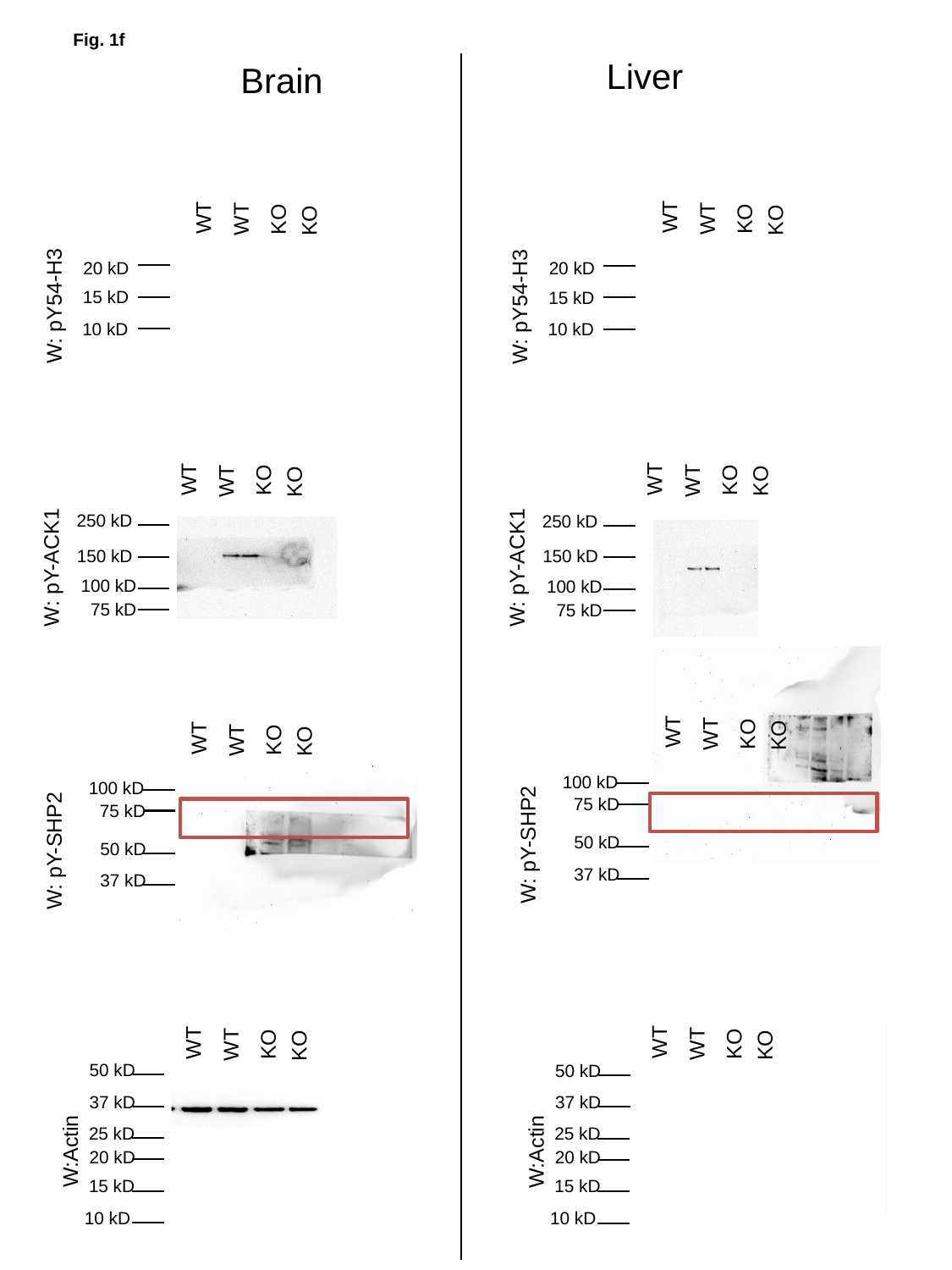

Fig. 1f
Liver
Brain
KO
KO
WT
WT
KO
KO
WT
WT
 20 kD
 20 kD
W: pY54-H3
W: pY54-H3
 15 kD
 15 kD
 10 kD
 10 kD
KO
KO
WT
WT
KO
KO
WT
WT
250 kD
250 kD
W: pY-ACK1
W: pY-ACK1
150 kD
150 kD
 100 kD
 100 kD
 75 kD
 75 kD
KO
WT
KO
WT
KO
KO
WT
WT
 100 kD
 100 kD
 75 kD
 75 kD
W: pY-SHP2
W: pY-SHP2
 50 kD
 50 kD
 37 kD
 37 kD
KO
KO
WT
WT
KO
KO
WT
WT
 50 kD
 50 kD
 37 kD
 37 kD
W:Actin
W:Actin
 25 kD
 25 kD
 20 kD
 20 kD
 15 kD
 15 kD
 10 kD
 10 kD

## Slide 8
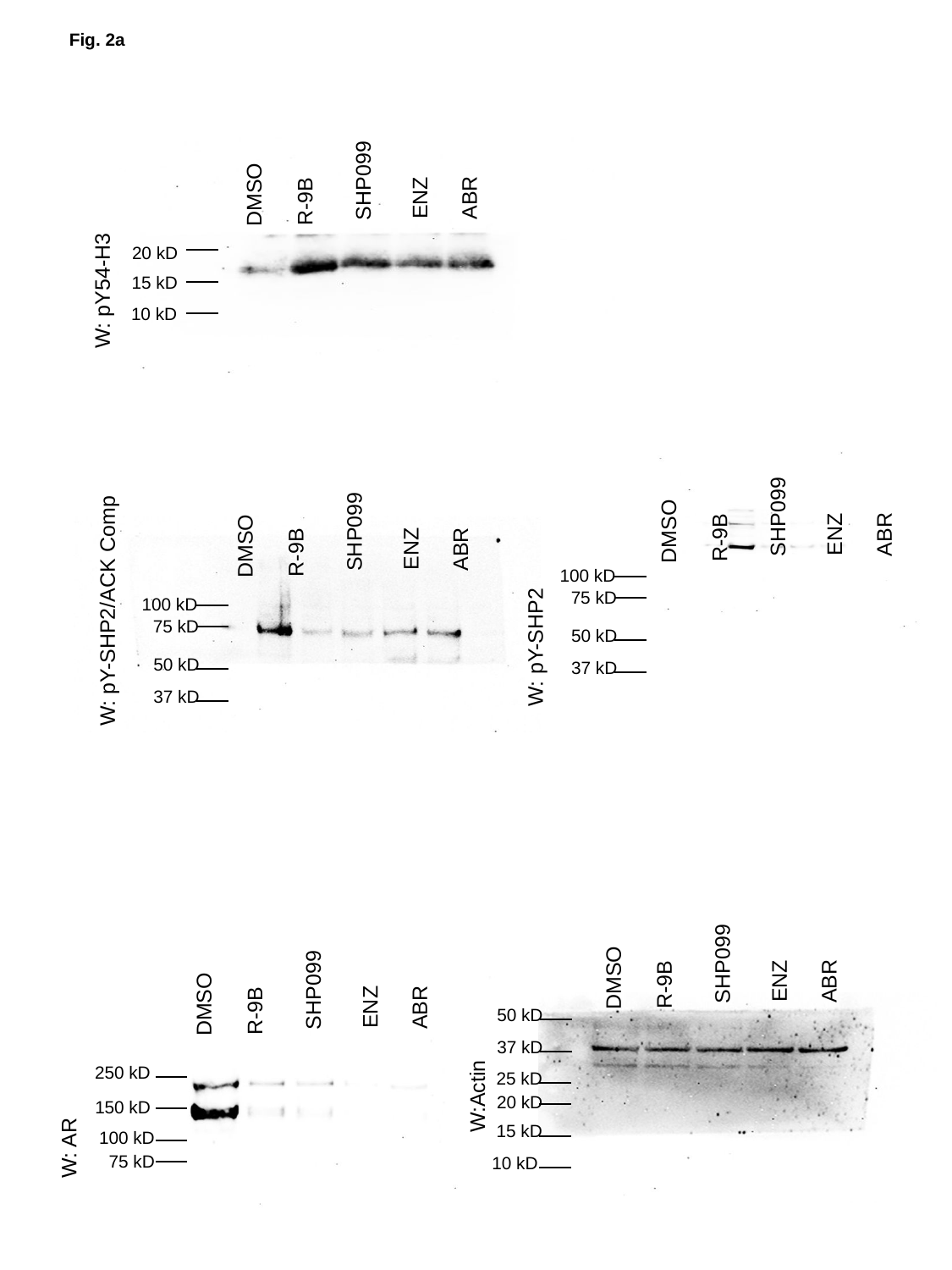

Fig. 2a
SHP099
ENZ
DMSO
ABR
R-9B
 20 kD
W: pY54-H3
 15 kD
 10 kD
SHP099
ENZ
DMSO
SHP099
ABR
R-9B
ENZ
DMSO
ABR
R-9B
 100 kD
W: pY-SHP2/ACK Comp
 75 kD
 100 kD
W: pY-SHP2
 75 kD
 50 kD
 50 kD
 37 kD
 37 kD
SHP099
ENZ
DMSO
ABR
R-9B
SHP099
ENZ
DMSO
ABR
R-9B
 50 kD
 37 kD
W:Actin
250 kD
 25 kD
W: AR
 20 kD
150 kD
 15 kD
 100 kD
 75 kD
 10 kD

## Slide 9
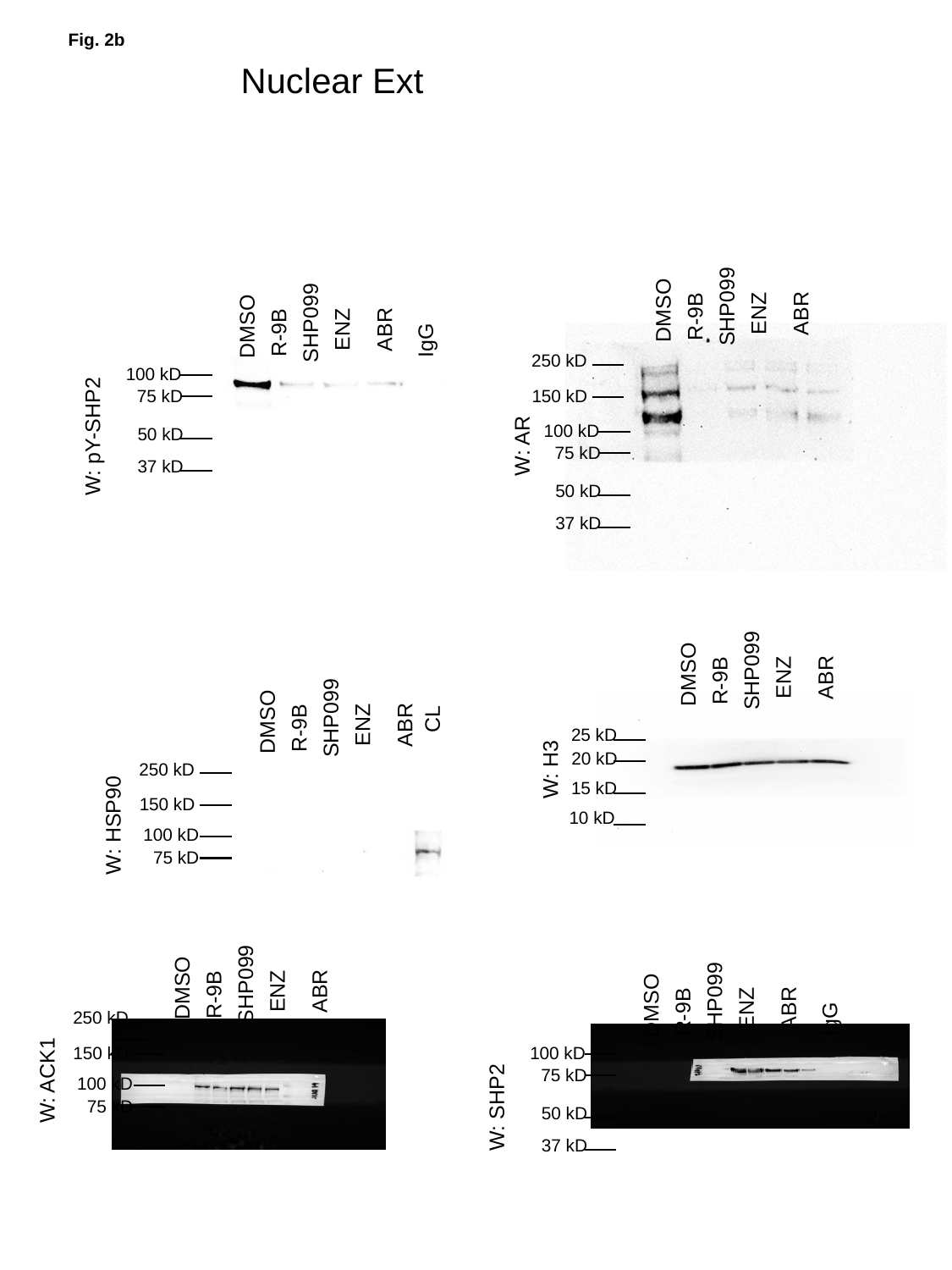

Fig. 2b
Nuclear Ext
SHP099
ENZ
DMSO
ABR
R-9B
SHP099
ENZ
DMSO
ABR
R-9B
IgG
250 kD
 100 kD
150 kD
 75 kD
W: AR
W: pY-SHP2
 100 kD
 50 kD
 75 kD
 37 kD
 50 kD
 37 kD
SHP099
ENZ
DMSO
ABR
R-9B
SHP099
CL
ENZ
DMSO
ABR
R-9B
W: H3
 25 kD
 20 kD
250 kD
W: HSP90
 15 kD
150 kD
 10 kD
 100 kD
 75 kD
SHP099
ENZ
DMSO
ABR
R-9B
SHP099
ENZ
DMSO
ABR
R-9B
IgG
250 kD
W: ACK1
150 kD
 100 kD
 75 kD
W: SHP2
 100 kD
 75 kD
 50 kD
 37 kD

## Slide 10
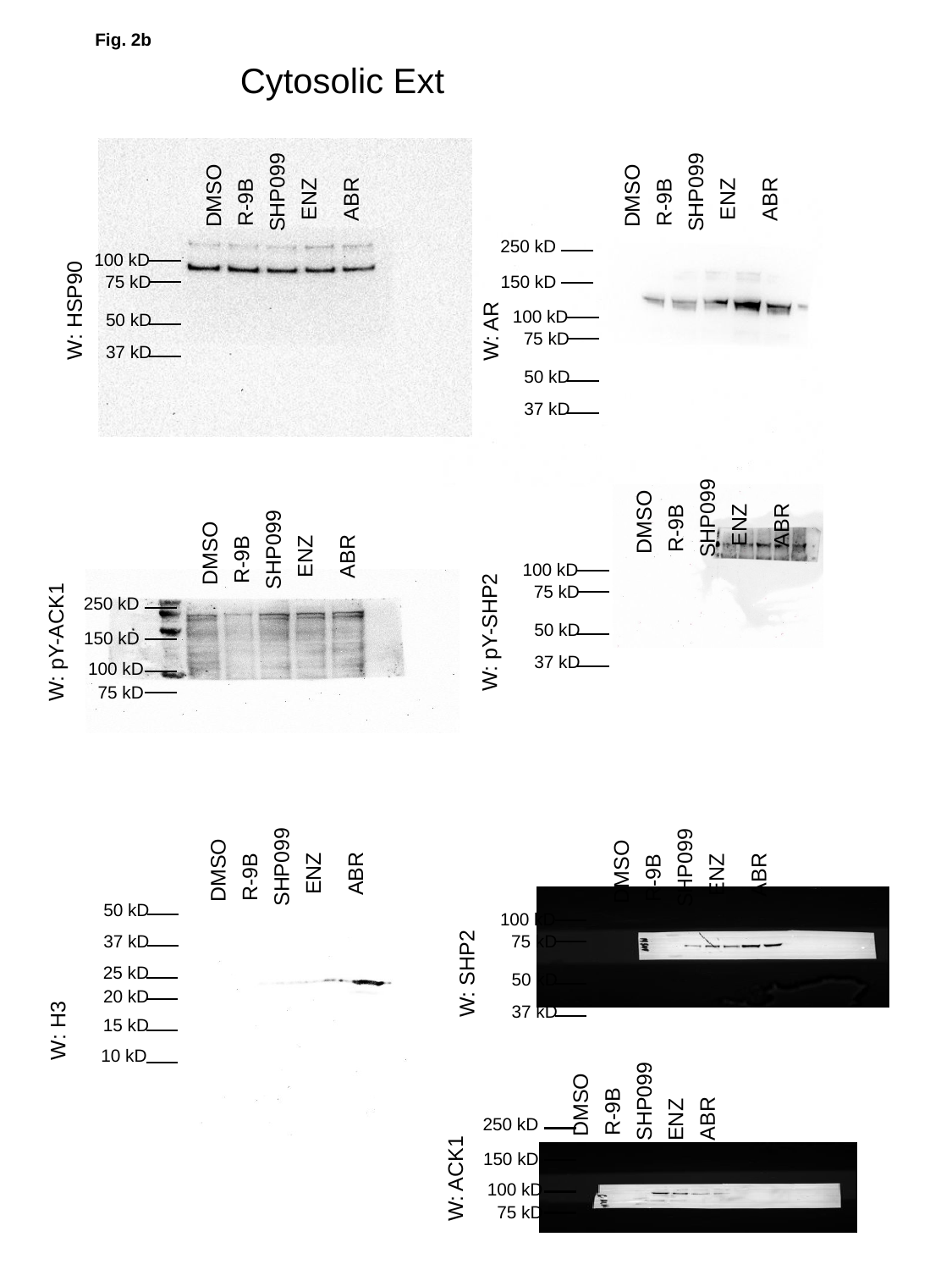

Fig. 2b
Cytosolic Ext
SHP099
SHP099
ENZ
DMSO
ENZ
DMSO
ABR
ABR
R-9B
R-9B
250 kD
W: HSP90
 100 kD
150 kD
 75 kD
W: AR
 100 kD
 50 kD
 75 kD
 37 kD
 50 kD
 37 kD
SHP099
ENZ
DMSO
ABR
R-9B
SHP099
ENZ
DMSO
ABR
R-9B
 100 kD
 75 kD
250 kD
W: pY-ACK1
W: pY-SHP2
 50 kD
150 kD
 37 kD
 100 kD
 75 kD
SHP099
SHP099
ENZ
DMSO
ENZ
DMSO
ABR
ABR
R-9B
R-9B
 50 kD
 100 kD
 75 kD
 37 kD
W: SHP2
 25 kD
W: H3
 50 kD
 20 kD
 37 kD
 15 kD
 10 kD
SHP099
DMSO
R-9B
ENZ
ABR
250 kD
W: ACK1
150 kD
 100 kD
 75 kD

## Slide 11
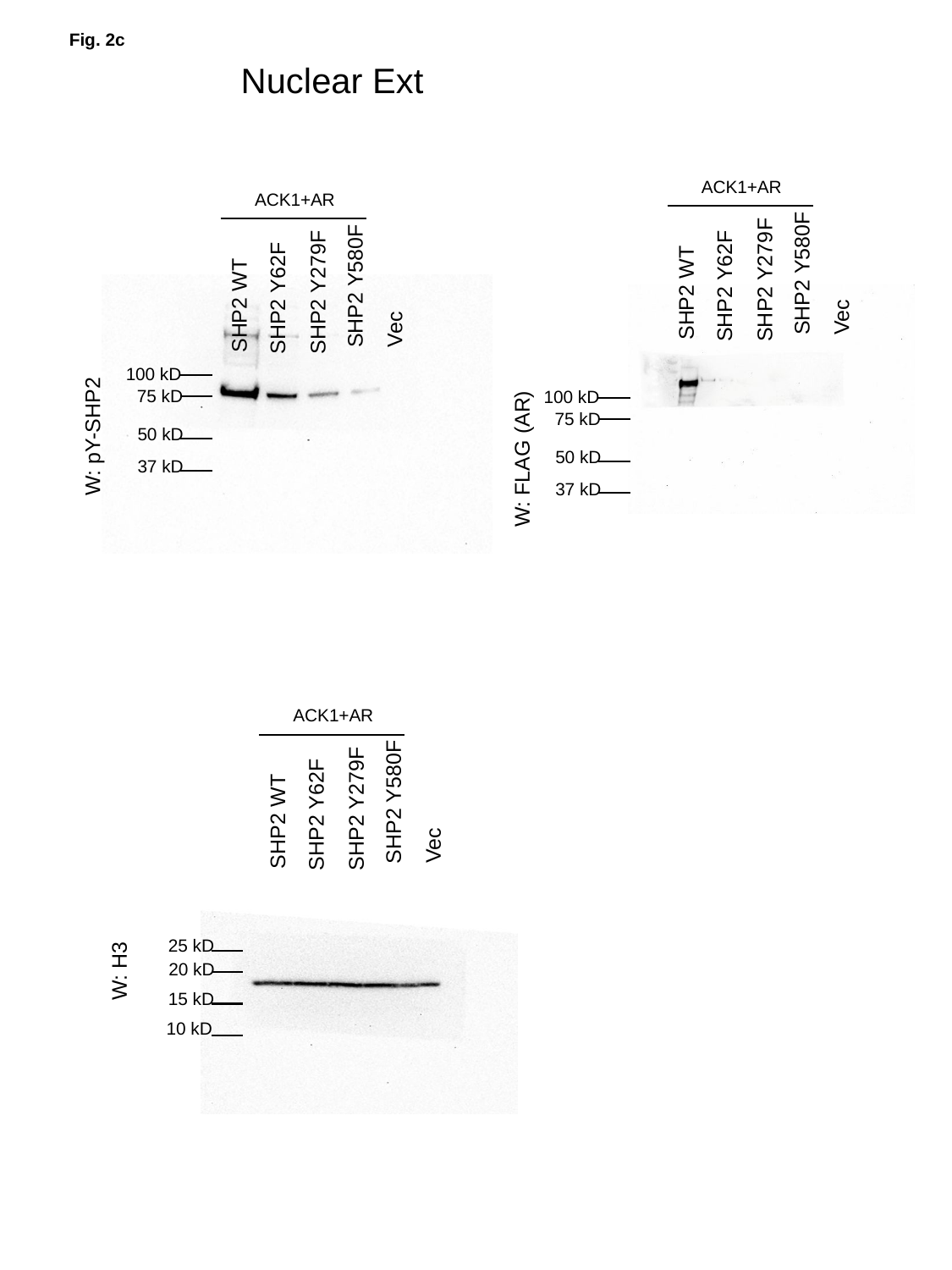

Fig. 2c
Nuclear Ext
ACK1+AR
ACK1+AR
SHP2 Y580F
SHP2 Y279F
SHP2 Y62F
SHP2 Y580F
SHP2 Y279F
SHP2 WT
SHP2 Y62F
SHP2 WT
Vec
Vec
 100 kD
 75 kD
 100 kD
 75 kD
W: pY-SHP2
 50 kD
W: FLAG (AR)
 50 kD
 37 kD
 37 kD
ACK1+AR
SHP2 Y580F
SHP2 Y279F
SHP2 Y62F
SHP2 WT
Vec
W: H3
 25 kD
 20 kD
 15 kD
 10 kD

## Slide 12
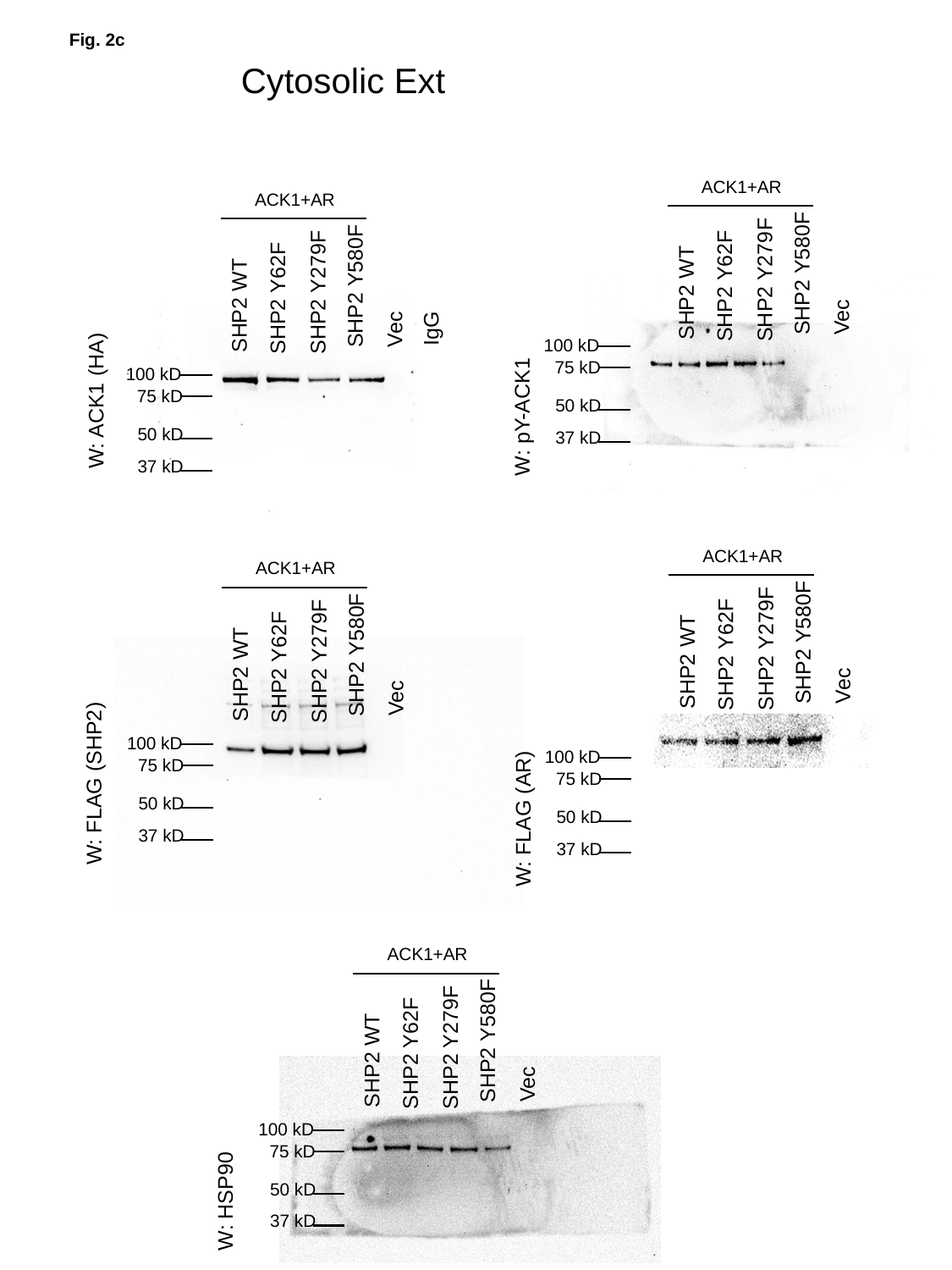

Fig. 2c
Cytosolic Ext
ACK1+AR
ACK1+AR
SHP2 Y580F
SHP2 Y279F
SHP2 Y62F
SHP2 Y580F
SHP2 Y279F
SHP2 WT
SHP2 Y62F
SHP2 WT
Vec
IgG
Vec
 100 kD
 75 kD
 100 kD
W: ACK1 (HA)
W: pY-ACK1
 75 kD
 50 kD
 50 kD
 37 kD
 37 kD
ACK1+AR
ACK1+AR
SHP2 Y580F
SHP2 Y279F
SHP2 Y62F
SHP2 Y580F
SHP2 Y279F
SHP2 WT
SHP2 Y62F
SHP2 WT
Vec
Vec
 100 kD
 100 kD
 75 kD
W: FLAG (SHP2)
 75 kD
W: FLAG (AR)
 50 kD
 50 kD
 37 kD
 37 kD
ACK1+AR
SHP2 Y580F
SHP2 Y279F
SHP2 Y62F
SHP2 WT
Vec
 100 kD
 75 kD
W: HSP90
 50 kD
 37 kD

## Slide 13
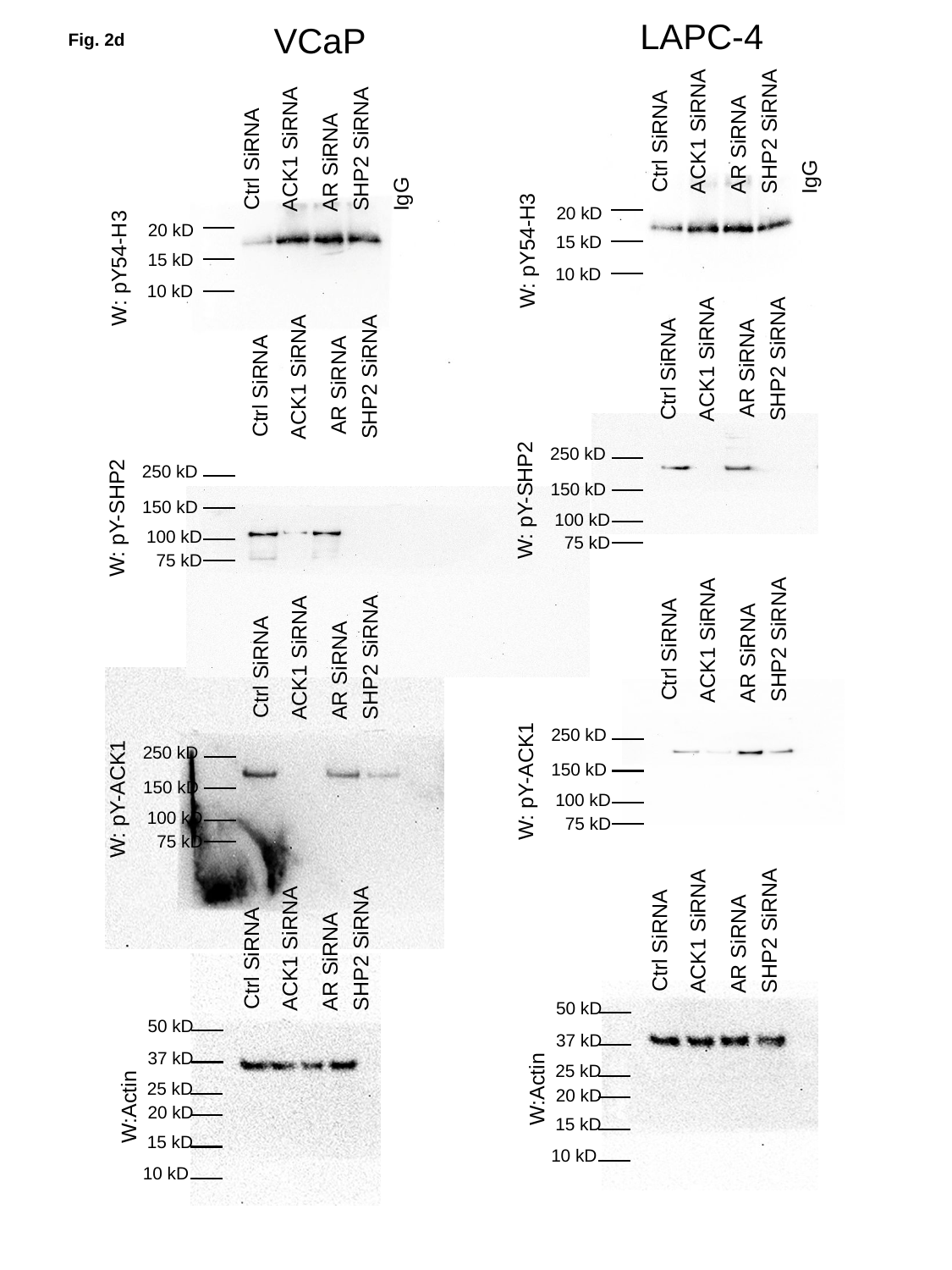

LAPC-4
VCaP
Fig. 2d
IgG
AR SiRNA
Ctrl SiRNA
SHP2 SiRNA
ACK1 SiRNA
IgG
AR SiRNA
Ctrl SiRNA
SHP2 SiRNA
ACK1 SiRNA
 20 kD
 20 kD
W: pY54-H3
 15 kD
W: pY54-H3
 15 kD
 10 kD
 10 kD
AR SiRNA
Ctrl SiRNA
SHP2 SiRNA
ACK1 SiRNA
AR SiRNA
Ctrl SiRNA
SHP2 SiRNA
ACK1 SiRNA
250 kD
W: pY-SHP2
250 kD
W: pY-SHP2
150 kD
150 kD
 100 kD
 100 kD
 75 kD
 75 kD
AR SiRNA
Ctrl SiRNA
SHP2 SiRNA
ACK1 SiRNA
AR SiRNA
Ctrl SiRNA
SHP2 SiRNA
ACK1 SiRNA
250 kD
W: pY-ACK1
250 kD
W: pY-ACK1
150 kD
150 kD
 100 kD
 100 kD
 75 kD
 75 kD
AR SiRNA
Ctrl SiRNA
SHP2 SiRNA
ACK1 SiRNA
AR SiRNA
Ctrl SiRNA
SHP2 SiRNA
ACK1 SiRNA
 50 kD
 50 kD
 37 kD
 37 kD
W:Actin
 25 kD
W:Actin
 25 kD
 20 kD
 20 kD
 15 kD
 15 kD
 10 kD
 10 kD

## Slide 14
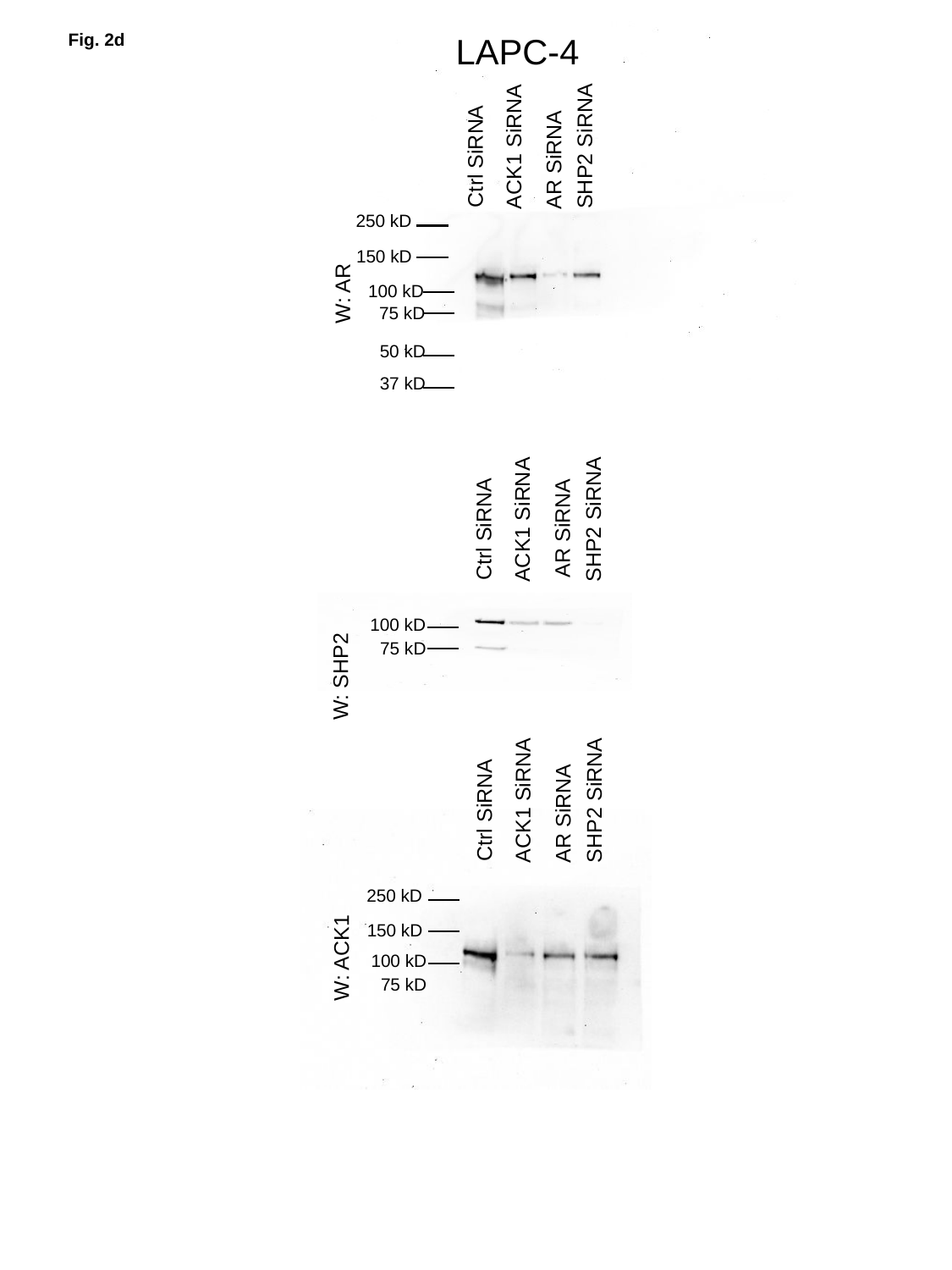

Fig. 2d
LAPC-4
AR SiRNA
Ctrl SiRNA
SHP2 SiRNA
ACK1 SiRNA
250 kD
W: AR
150 kD
 100 kD
 75 kD
 50 kD
 37 kD
AR SiRNA
Ctrl SiRNA
SHP2 SiRNA
ACK1 SiRNA
 100 kD
W: SHP2
 75 kD
AR SiRNA
Ctrl SiRNA
SHP2 SiRNA
ACK1 SiRNA
250 kD
W: ACK1
150 kD
 100 kD
 75 kD

## Slide 15
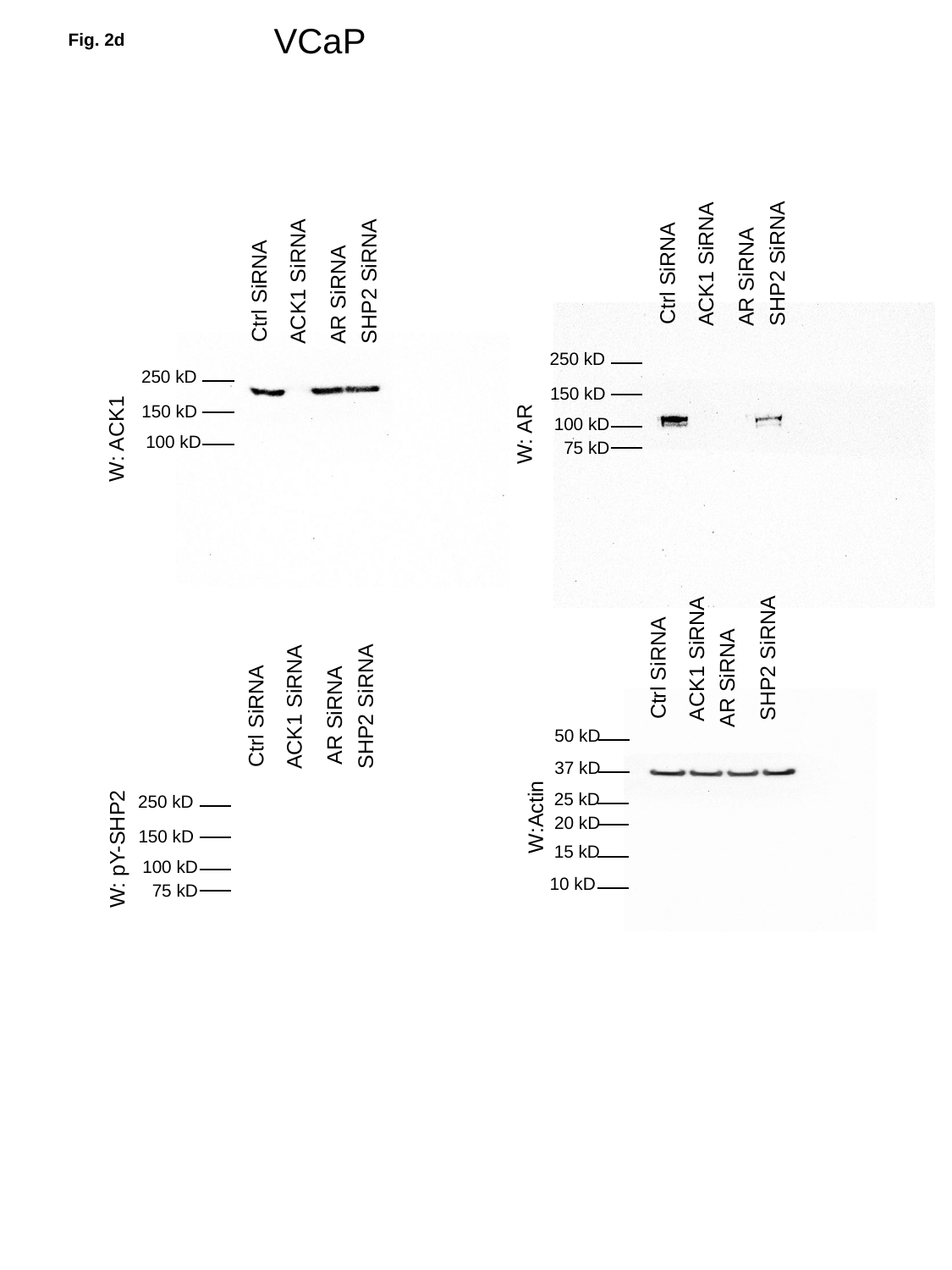

VCaP
Fig. 2d
AR SiRNA
Ctrl SiRNA
SHP2 SiRNA
ACK1 SiRNA
AR SiRNA
Ctrl SiRNA
SHP2 SiRNA
ACK1 SiRNA
250 kD
W: AR
250 kD
W: ACK1
150 kD
150 kD
 100 kD
 100 kD
 75 kD
AR SiRNA
Ctrl SiRNA
SHP2 SiRNA
ACK1 SiRNA
AR SiRNA
Ctrl SiRNA
SHP2 SiRNA
ACK1 SiRNA
 50 kD
 37 kD
W:Actin
 25 kD
250 kD
W: pY-SHP2
 20 kD
150 kD
 15 kD
 100 kD
 10 kD
 75 kD

## Slide 16
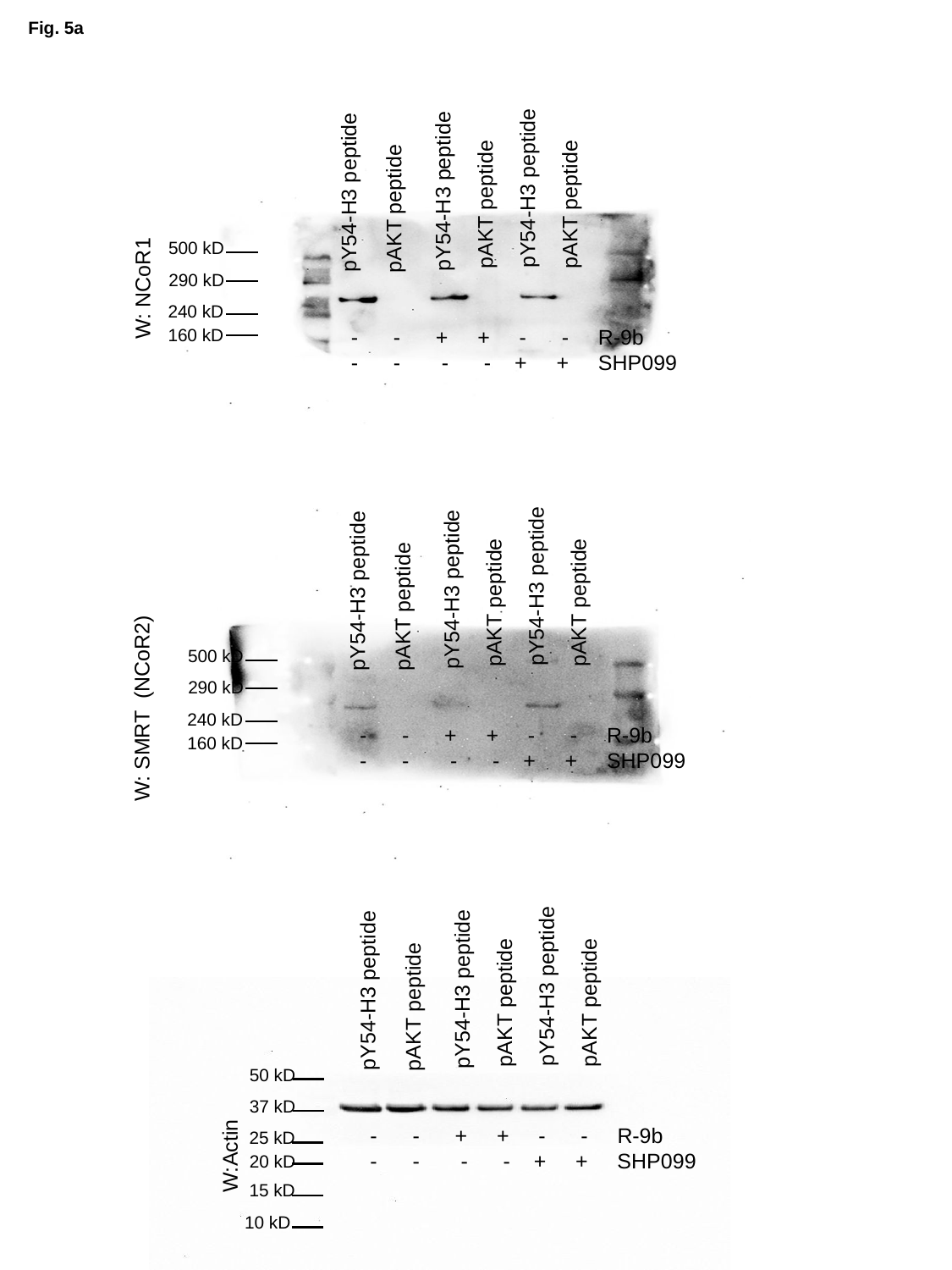

Fig. 5a
pY54-H3 peptide
pY54-H3 peptide
pY54-H3 peptide
pAKT peptide
pAKT peptide
pAKT peptide
500 kD
W: NCoR1
290 kD
240 kD
- - + + - - R-9b
- - - - + + SHP099
160 kD
pY54-H3 peptide
pY54-H3 peptide
pY54-H3 peptide
pAKT peptide
pAKT peptide
pAKT peptide
500 kD
W: SMRT (NCoR2)
290 kD
240 kD
- - + + - - R-9b
- - - - + + SHP099
160 kD
pY54-H3 peptide
pY54-H3 peptide
pY54-H3 peptide
pAKT peptide
pAKT peptide
pAKT peptide
 50 kD
 37 kD
W:Actin
- - + + - - R-9b
- - - - + + SHP099
 25 kD
 20 kD
 15 kD
 10 kD

## Slide 17
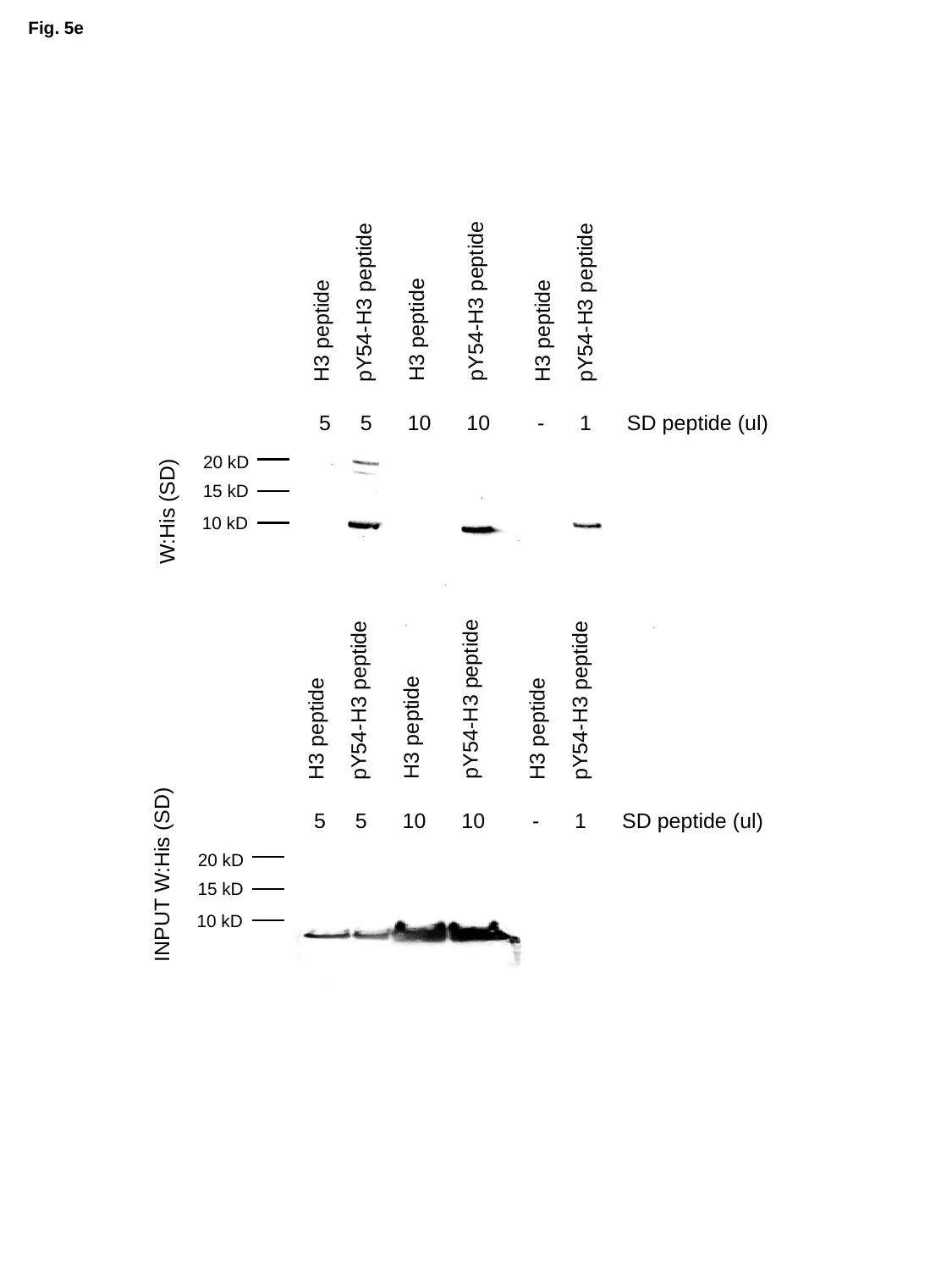

Fig. 5e
pY54-H3 peptide
pY54-H3 peptide
pY54-H3 peptide
H3 peptide
H3 peptide
H3 peptide
5 5 10 10 - 1 SD peptide (ul)
 20 kD
W:His (SD)
 15 kD
 10 kD
pY54-H3 peptide
pY54-H3 peptide
pY54-H3 peptide
H3 peptide
H3 peptide
H3 peptide
5 5 10 10 - 1 SD peptide (ul)
 20 kD
INPUT W:His (SD)
 15 kD
 10 kD

## Slide 18
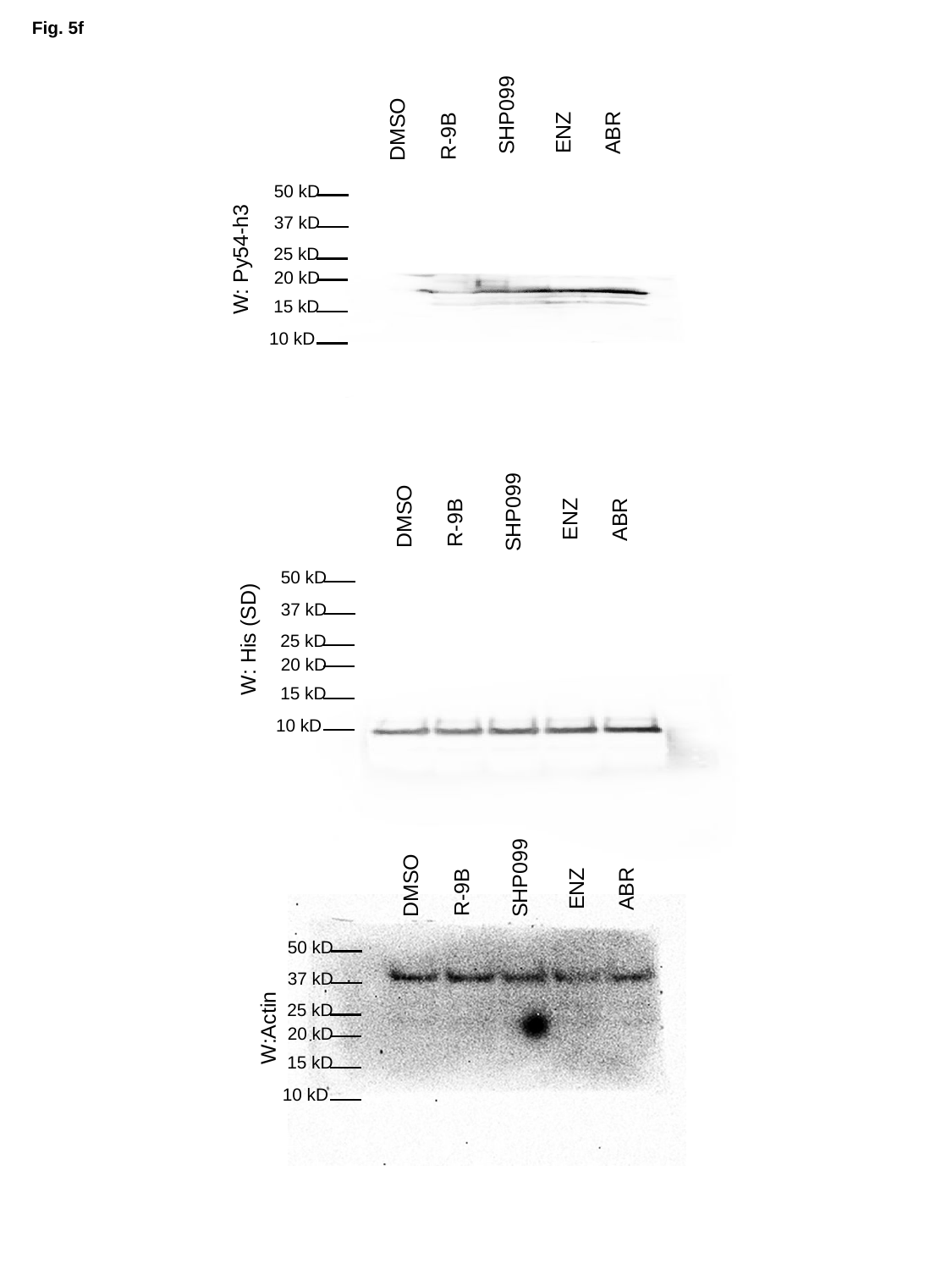

Fig. 5f
SHP099
ENZ
DMSO
ABR
R-9B
 50 kD
 37 kD
W: Py54-h3
 25 kD
 20 kD
 15 kD
 10 kD
SHP099
ENZ
DMSO
ABR
R-9B
 50 kD
 37 kD
W: His (SD)
 25 kD
 20 kD
 15 kD
 10 kD
SHP099
ENZ
DMSO
ABR
R-9B
 50 kD
 37 kD
W:Actin
 25 kD
 20 kD
 15 kD
 10 kD

## Slide 19
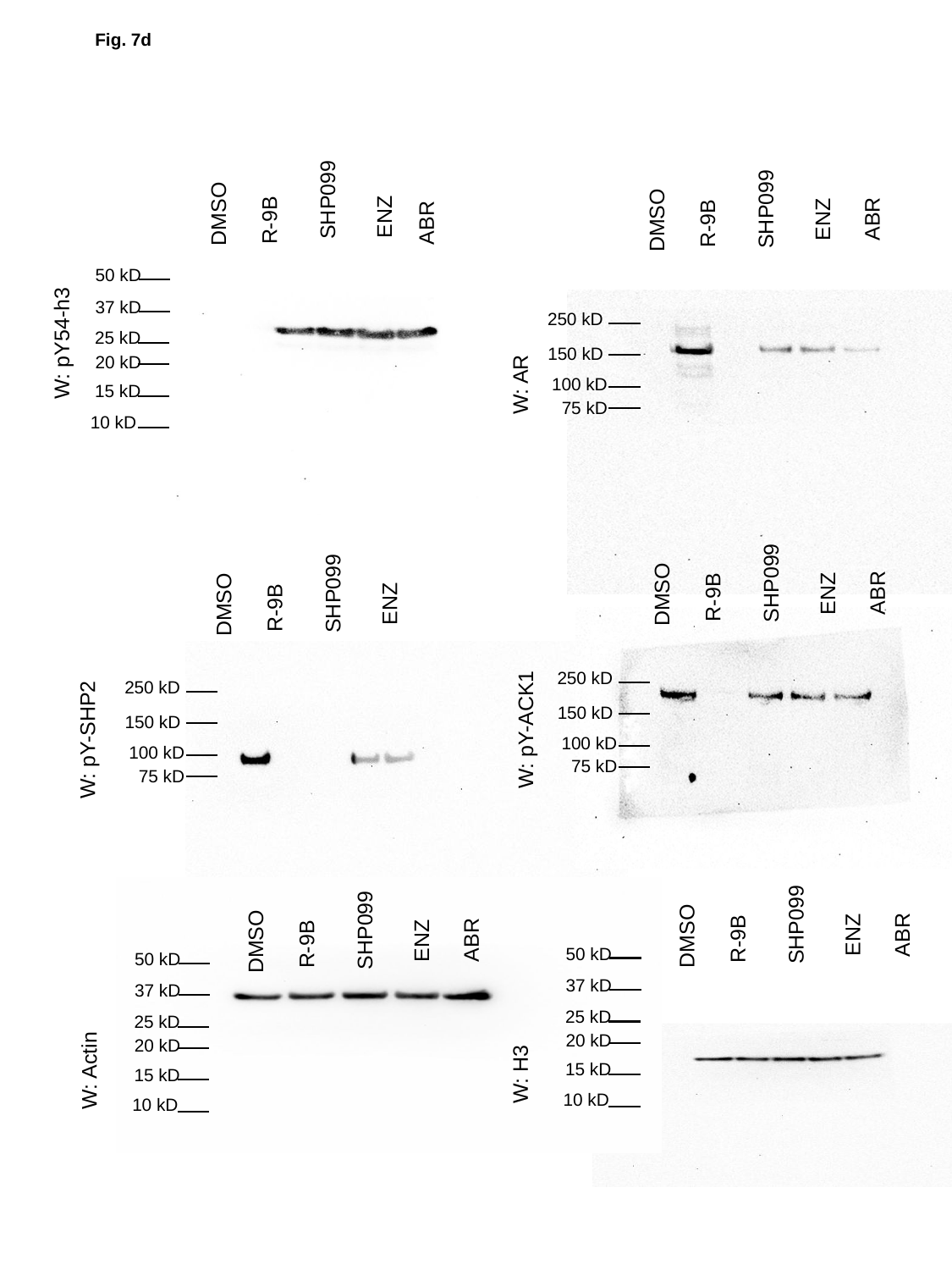

Fig. 7d
SHP099
SHP099
ENZ
DMSO
ABR
ENZ
DMSO
R-9B
ABR
R-9B
 50 kD
 37 kD
250 kD
W: AR
W: pY54-h3
 25 kD
150 kD
 20 kD
 100 kD
 15 kD
 75 kD
 10 kD
SHP099
SHP099
ENZ
DMSO
ABR
R-9B
ENZ
DMSO
R-9B
250 kD
250 kD
W: pY-ACK1
150 kD
W: pY-SHP2
150 kD
 100 kD
 100 kD
 75 kD
 75 kD
SHP099
SHP099
ENZ
DMSO
ABR
ENZ
R-9B
DMSO
ABR
R-9B
 50 kD
 50 kD
 37 kD
 37 kD
 25 kD
W: H3
 25 kD
W: Actin
 20 kD
 20 kD
 15 kD
 15 kD
 10 kD
 10 kD

## Slide 20
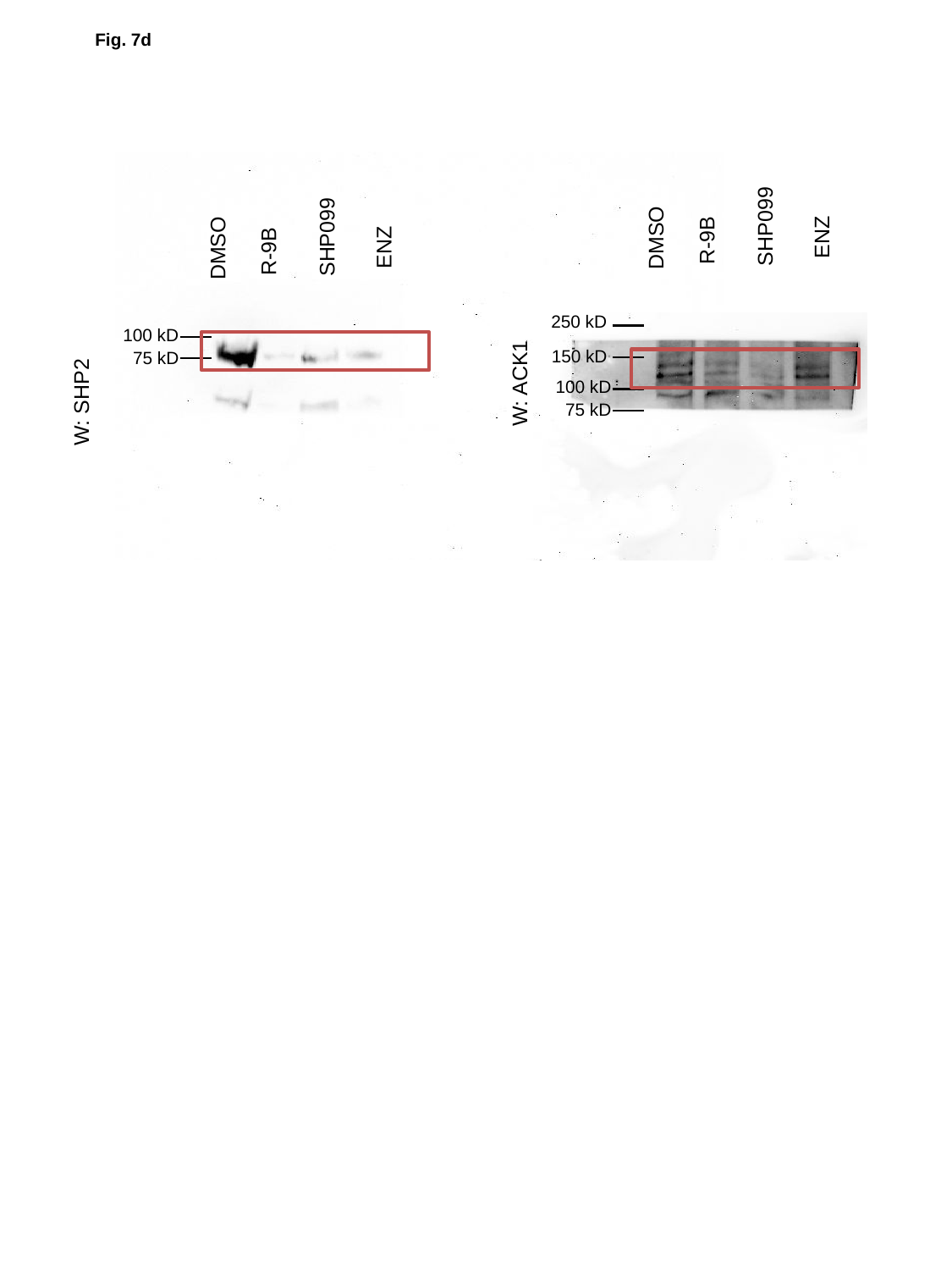

Fig. 7d
SHP099
SHP099
ENZ
DMSO
R-9B
ENZ
DMSO
R-9B
250 kD
 100 kD
W: ACK1
150 kD
 75 kD
W: SHP2
 100 kD
 75 kD

## Slide 21
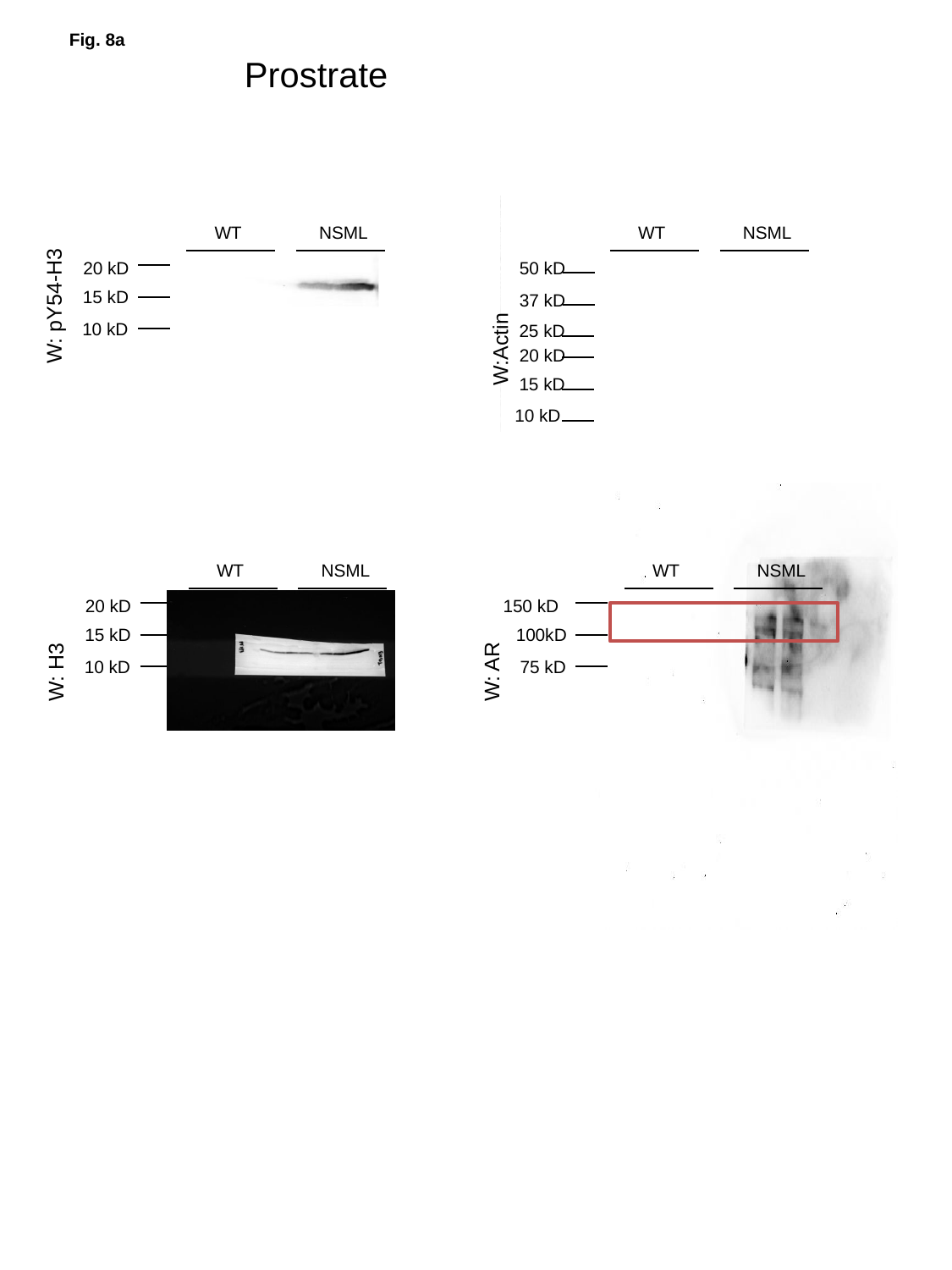

Fig. 8a
Prostrate
WT NSML
WT NSML
 20 kD
 50 kD
W: pY54-H3
 15 kD
 37 kD
W:Actin
 10 kD
 25 kD
 20 kD
 15 kD
 10 kD
WT NSML
WT NSML
 20 kD
 150 kD
W: H3
W: AR
 15 kD
 100kD
 10 kD
 75 kD

## Slide 22
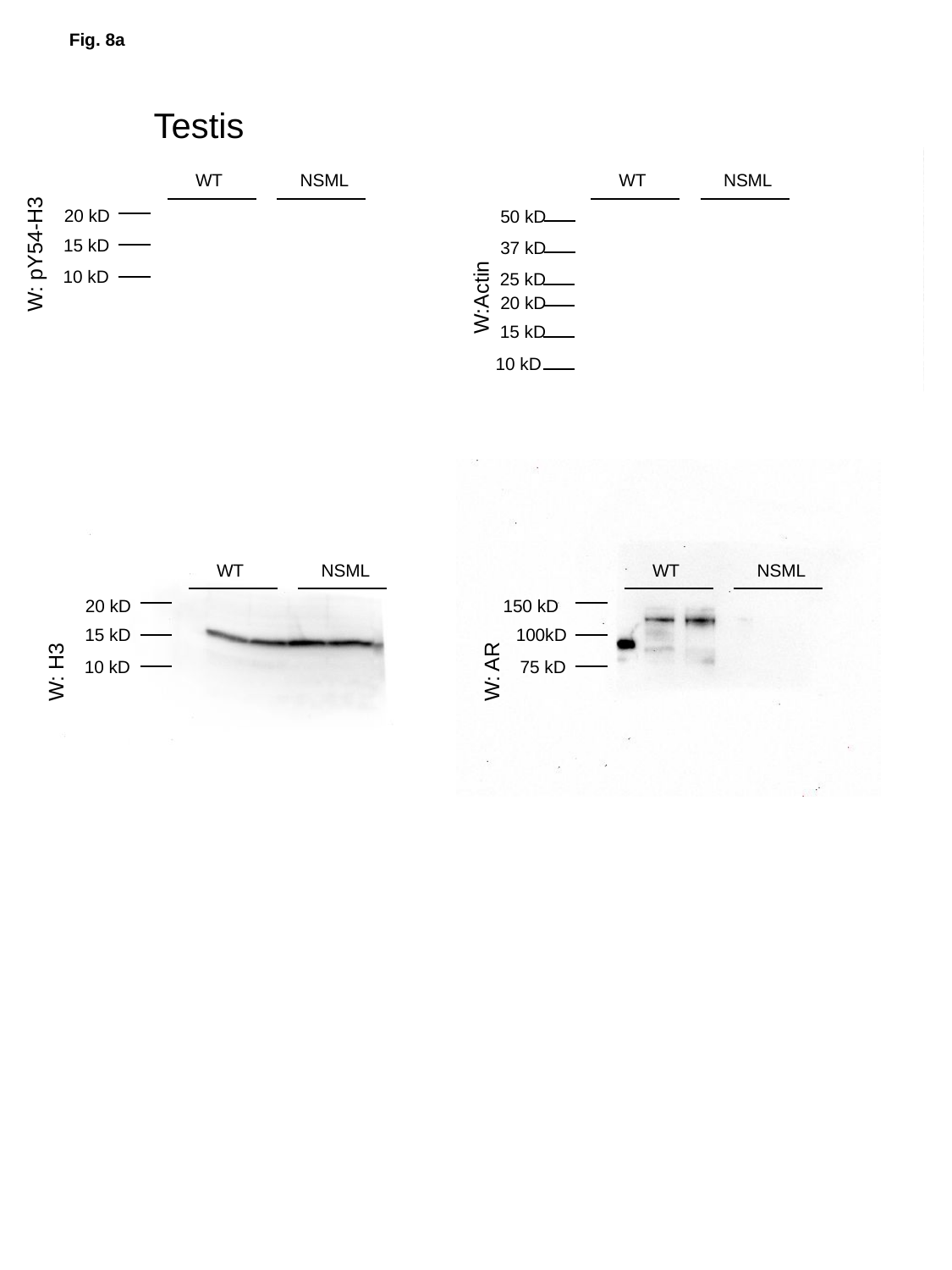

Fig. 8a
Testis
WT NSML
WT NSML
 20 kD
 50 kD
W: pY54-H3
 15 kD
 37 kD
W:Actin
 10 kD
 25 kD
 20 kD
 15 kD
 10 kD
WT NSML
WT NSML
 20 kD
 150 kD
W: H3
W: AR
 15 kD
 100kD
 10 kD
 75 kD

## Slide 23
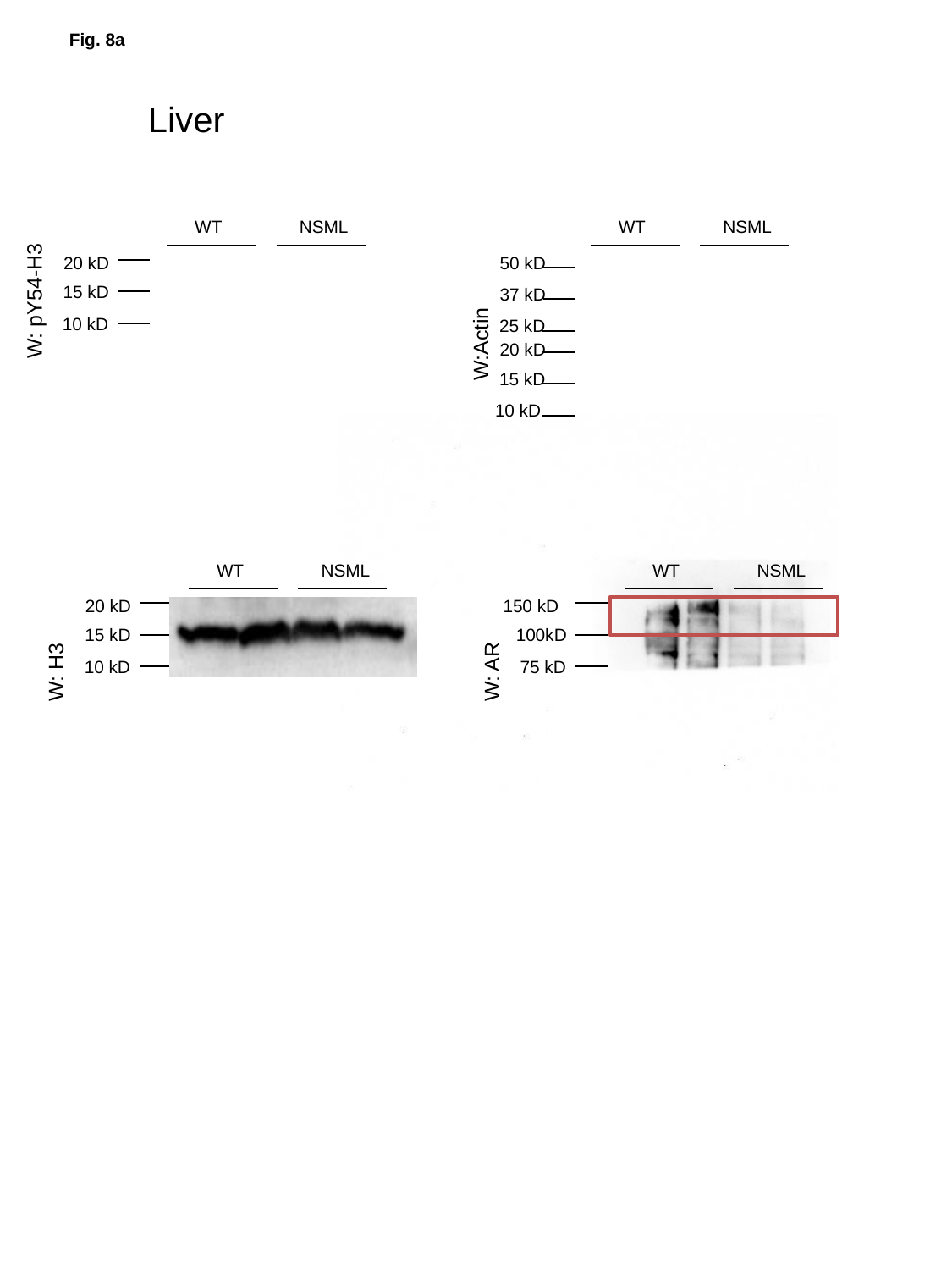

Fig. 8a
Liver
WT NSML
WT NSML
 20 kD
 50 kD
W: pY54-H3
 15 kD
 37 kD
W:Actin
 10 kD
 25 kD
 20 kD
 15 kD
 10 kD
WT NSML
WT NSML
 20 kD
 150 kD
W: H3
W: AR
 15 kD
 100kD
 10 kD
 75 kD

## Slide 24
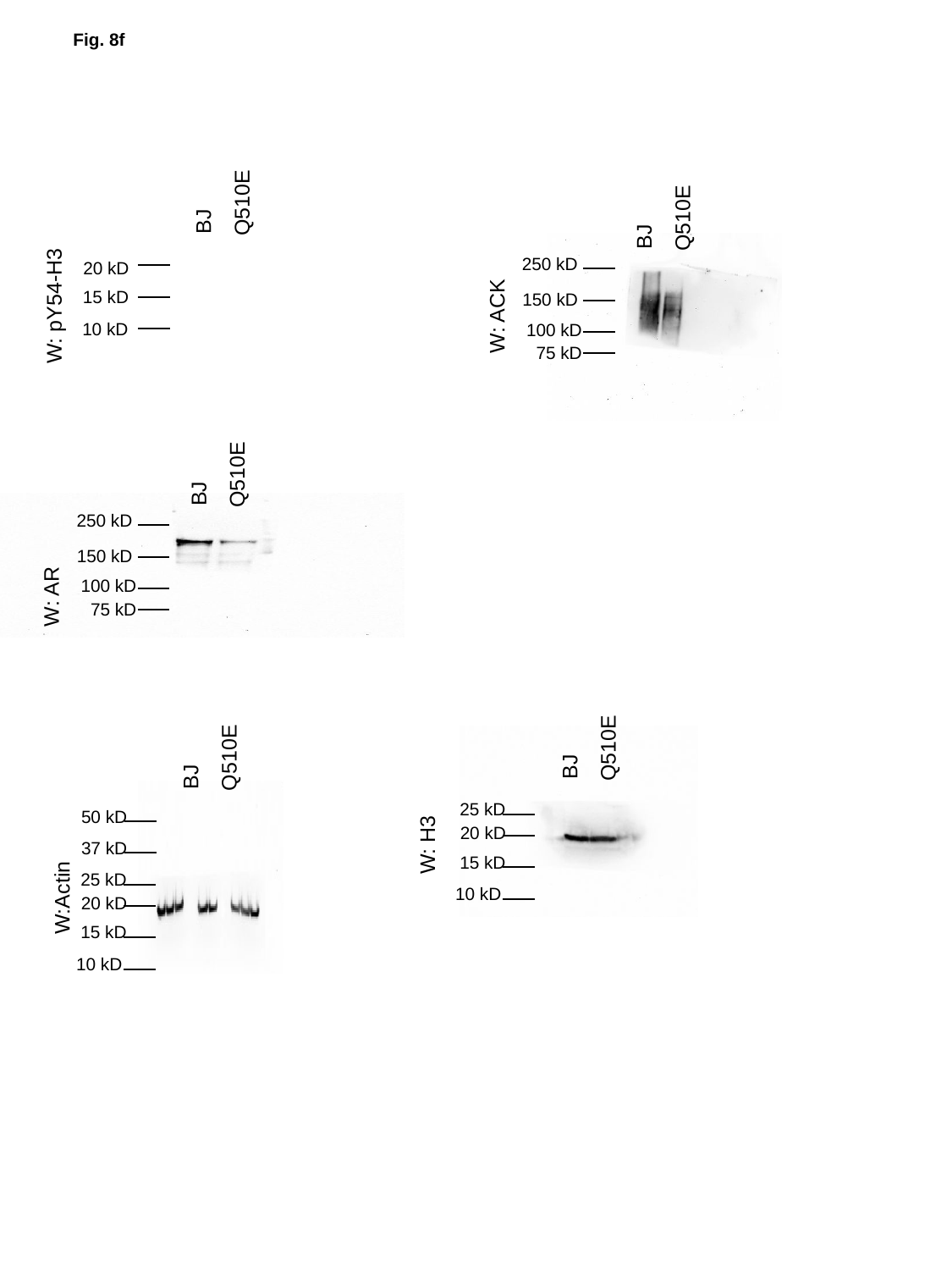

Fig. 8f
Q510E
BJ
Q510E
BJ
W: ACK
250 kD
 20 kD
W: pY54-H3
 15 kD
150 kD
 10 kD
 100 kD
 75 kD
Q510E
BJ
250 kD
W: AR
150 kD
 100 kD
 75 kD
Q510E
BJ
Q510E
BJ
W: H3
 25 kD
 50 kD
 20 kD
 37 kD
 15 kD
W:Actin
 25 kD
 10 kD
 20 kD
 15 kD
 10 kD

## Slide 25
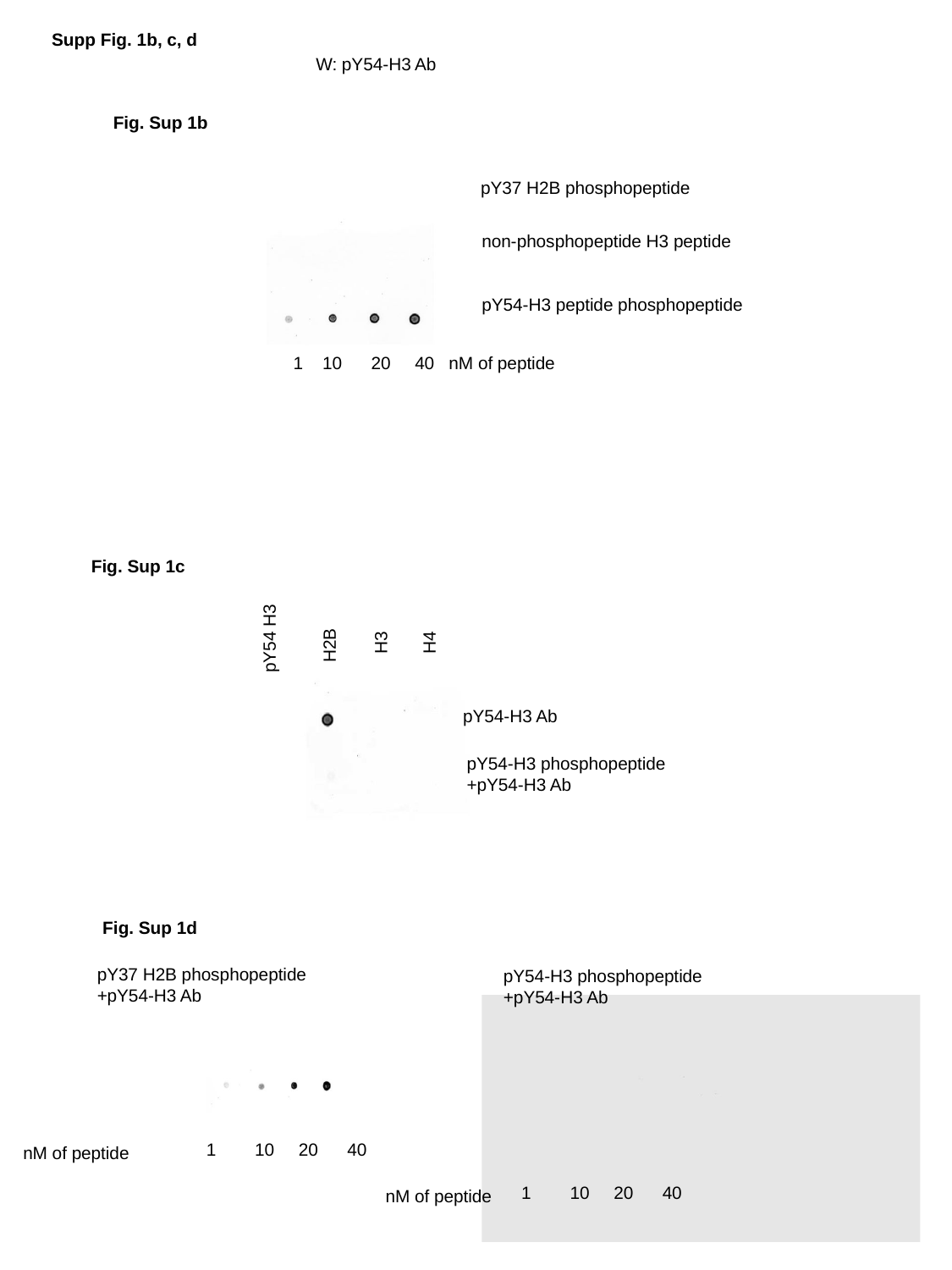

Supp Fig. 1b, c, d
W: pY54-H3 Ab
Fig. Sup 1b
pY37 H2B phosphopeptide
non-phosphopeptide H3 peptide
pY54-H3 peptide phosphopeptide
1 10 20 40 nM of peptide
Fig. Sup 1c
H3
H4
H2B
pY54 H3
pY54-H3 Ab
pY54-H3 phosphopeptide
+pY54-H3 Ab
Fig. Sup 1d
pY37 H2B phosphopeptide
+pY54-H3 Ab
pY54-H3 phosphopeptide
+pY54-H3 Ab
1 10 20 40
nM of peptide
1 10 20 40
nM of peptide

## Slide 26
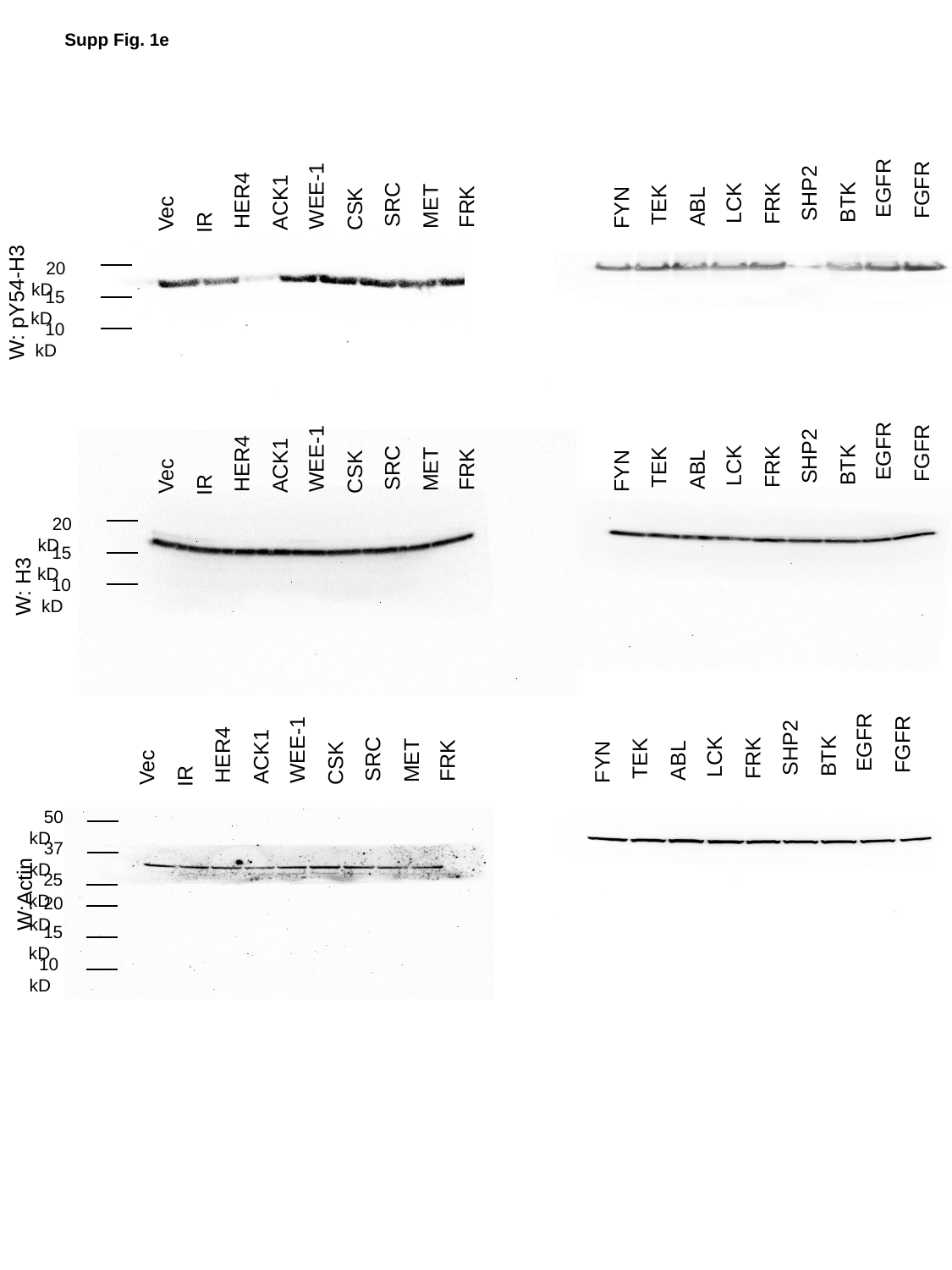

Supp Fig. 1e
FGFR
BTK
FRK
EGFR
ABL
MET
FYN
SHP2
ACK1
CSK
LCK
IR
TEK
SRC
FRK
HER4
WEE-1
Vec
 20 kD
W: pY54-H3
 15 kD
 10 kD
FGFR
BTK
FRK
EGFR
ABL
MET
FYN
SHP2
ACK1
CSK
LCK
IR
TEK
SRC
FRK
HER4
WEE-1
Vec
 20 kD
W: H3
 15 kD
 10 kD
FGFR
BTK
FRK
EGFR
ABL
MET
FYN
SHP2
ACK1
CSK
LCK
IR
TEK
SRC
FRK
HER4
WEE-1
Vec
 50 kD
 37 kD
W:Actin
 25 kD
 20 kD
 15 kD
 10 kD

## Slide 27
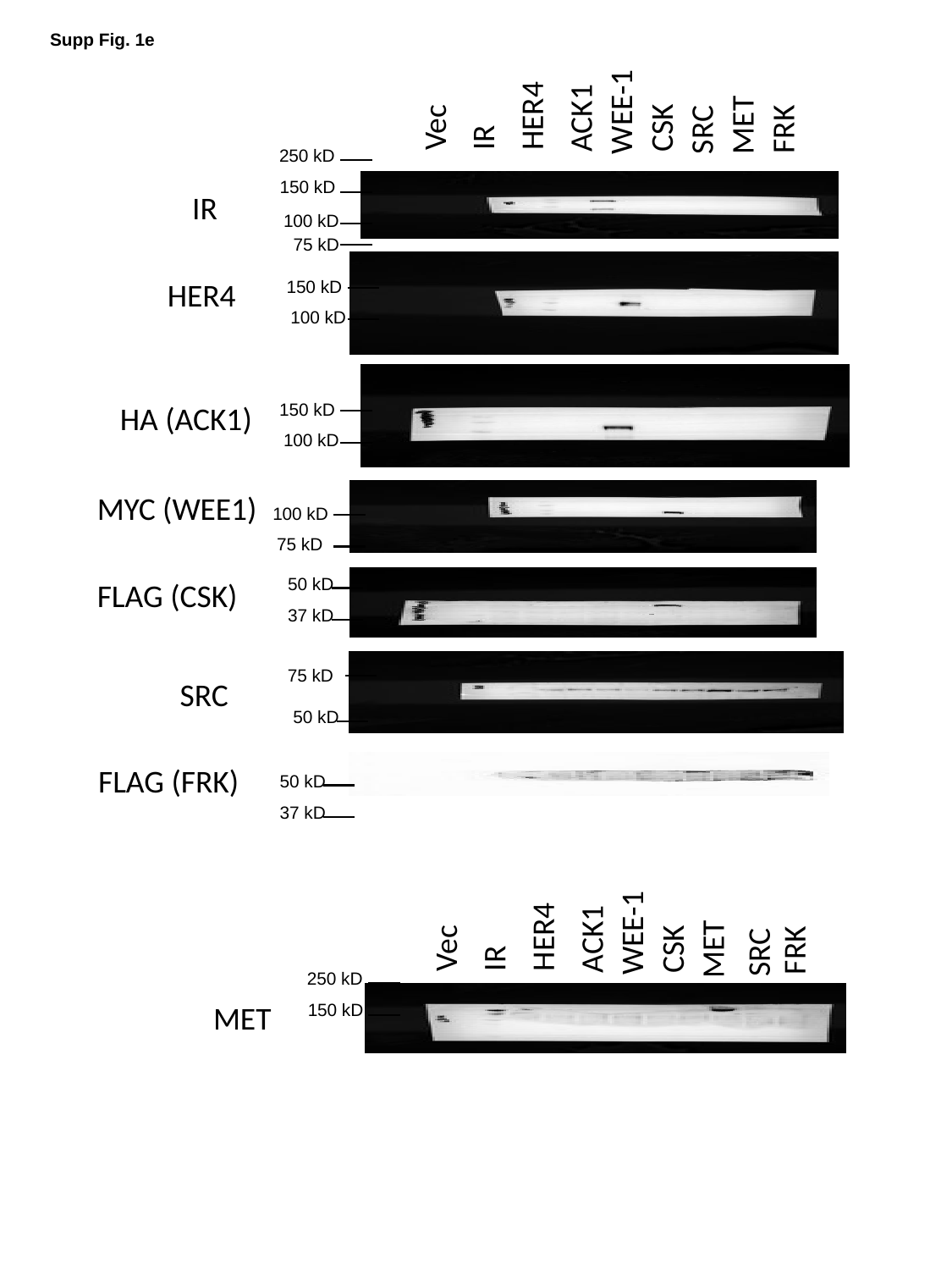

Supp Fig. 1e
Vec
IR
HER4
ACK1
CSK
FRK
WEE-1
SRC
MET
250 kD
150 kD
IR
 100 kD
 75 kD
HER4
150 kD
MET
 100 kD
HA (ACK1)
150 kD
 100 kD
MYC (WEE1)
100 kD
 75 kD
 50 kD
FLAG (CSK)
 37 kD
75 kD
SRC
 50 kD
FLAG (FRK)
 50 kD
 37 kD
Vec
IR
HER4
ACK1
CSK
FRK
WEE-1
SRC
MET
250 kD
MET
150 kD

## Slide 28
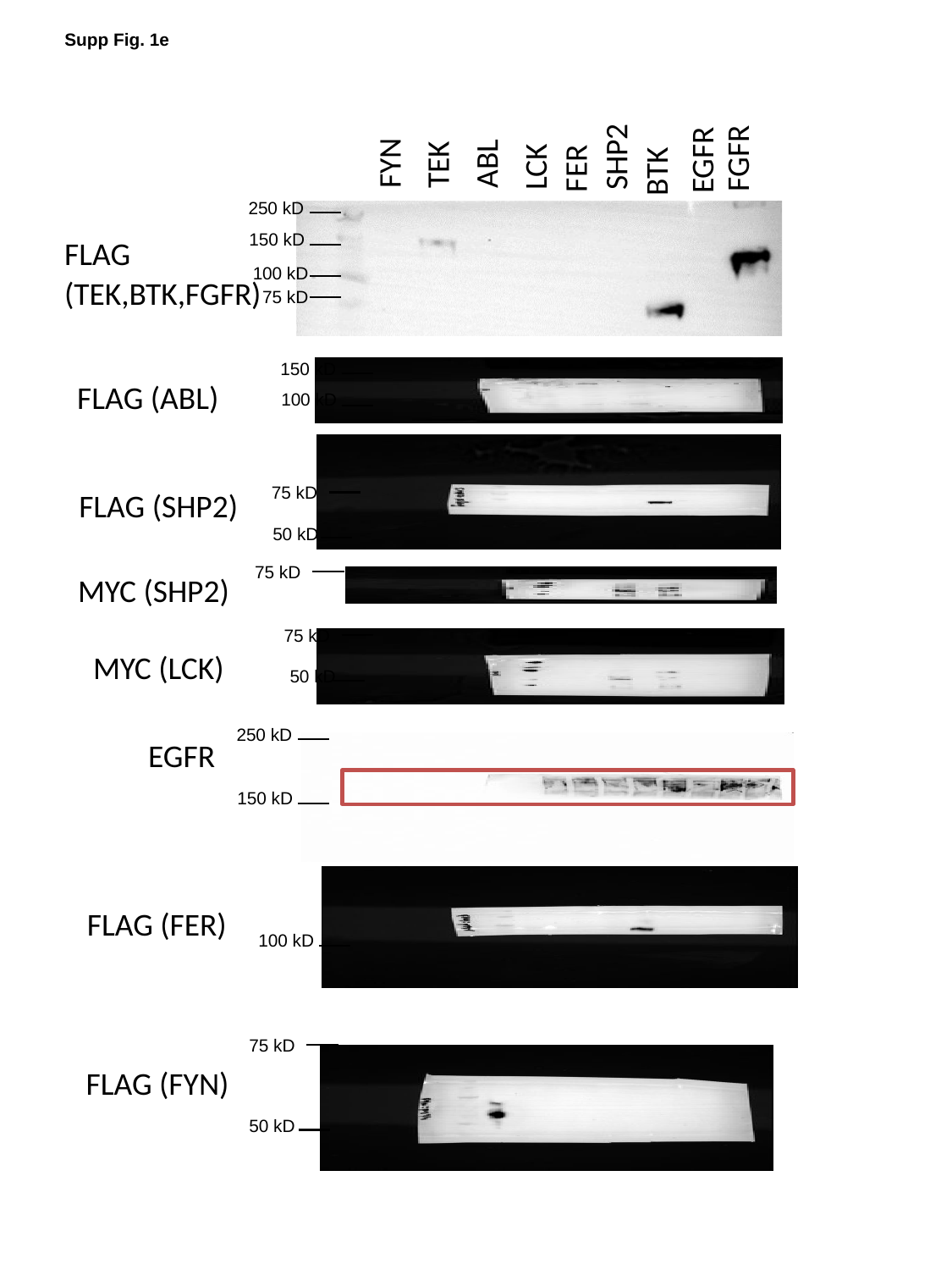

Supp Fig. 1e
FYN
TEK
ABL
LCK
SHP2
FGFR
FER
EGFR
BTK
250 kD
150 kD
FLAG (TEK,BTK,FGFR)
 100 kD
 75 kD
150 kD
FLAG (ABL)
100 kD
75 kD
FLAG (SHP2)
 50 kD
75 kD
MYC (SHP2)
75 kD
MYC (LCK)
 50 kD
250 kD
EGFR
150 kD
FLAG (FER)
100 kD
75 kD
FLAG (FYN)
 50 kD

## Slide 29
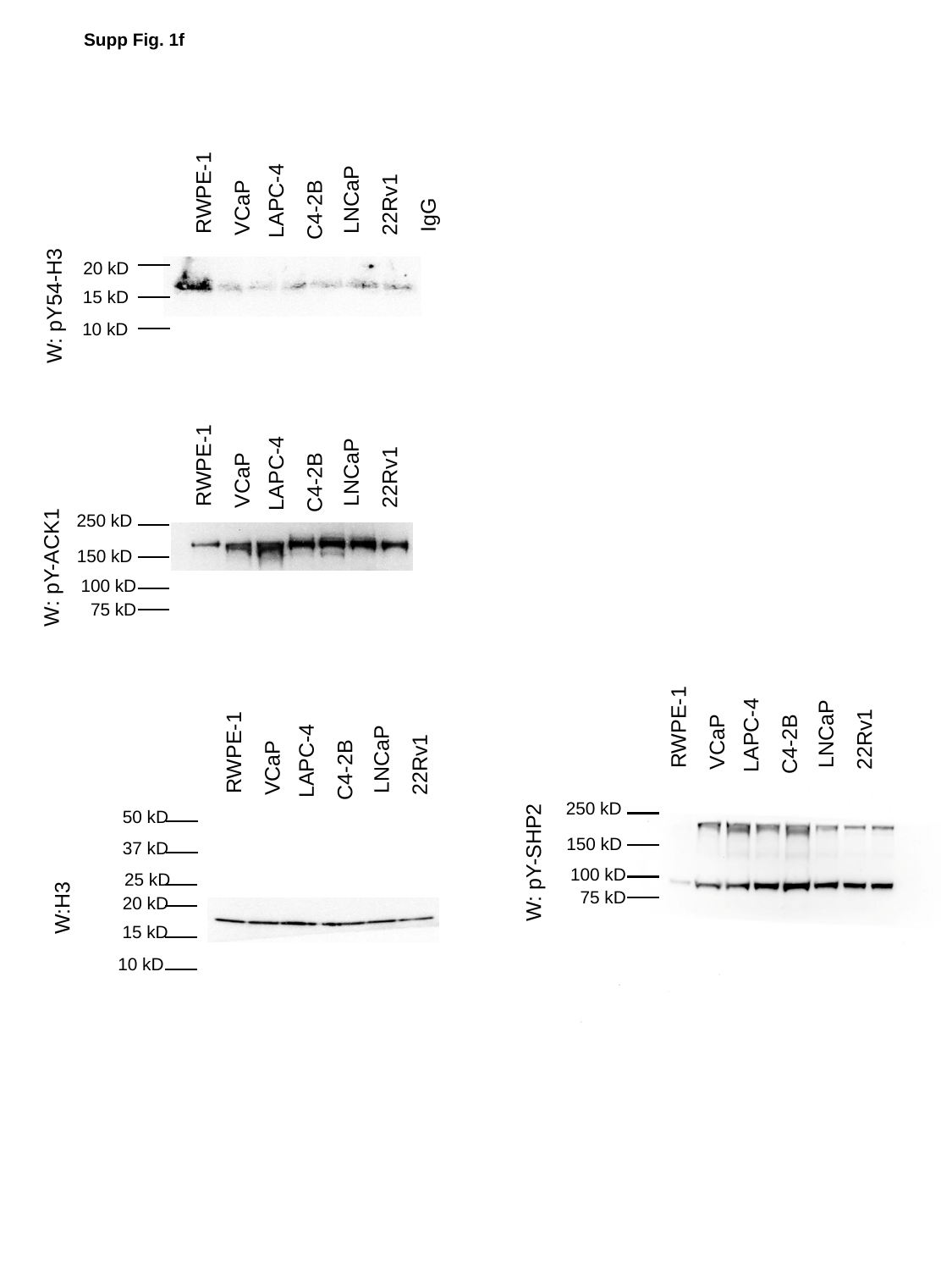

Supp Fig. 1f
22Rv1
VCaP
C4-2B
IgG
LNCaP
RWPE-1
LAPC-4
 20 kD
W: pY54-H3
 15 kD
 10 kD
22Rv1
VCaP
C4-2B
LNCaP
RWPE-1
LAPC-4
250 kD
W: pY-ACK1
150 kD
 100 kD
 75 kD
22Rv1
VCaP
C4-2B
LNCaP
RWPE-1
LAPC-4
22Rv1
VCaP
C4-2B
LNCaP
RWPE-1
LAPC-4
250 kD
 50 kD
150 kD
 37 kD
W: pY-SHP2
W:H3
 100 kD
 25 kD
 75 kD
 20 kD
 15 kD
 10 kD

## Slide 30
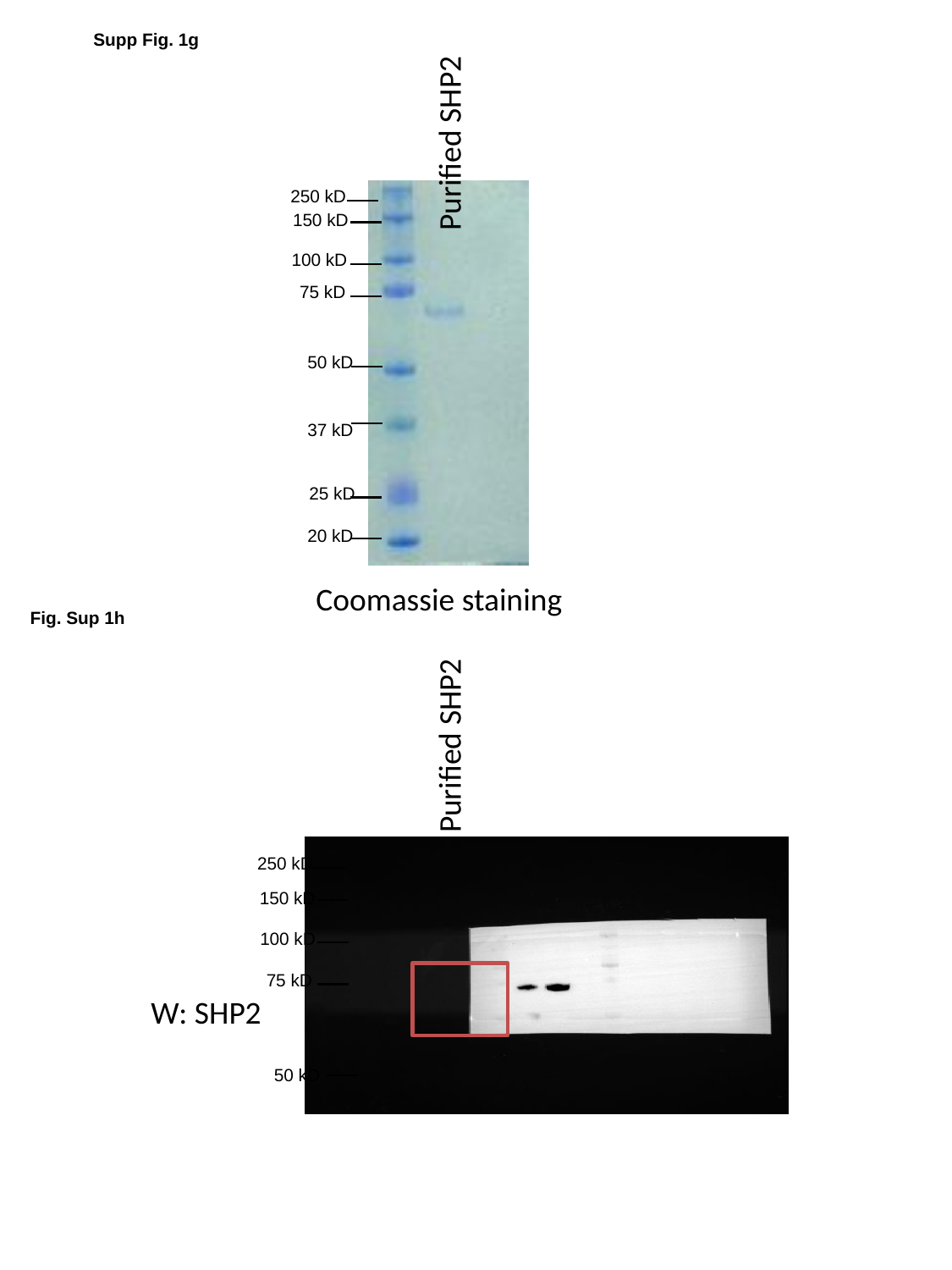

Supp Fig. 1g
Purified SHP2
250 kD
150 kD
 100 kD
 75 kD
 50 kD
 37 kD
 25 kD
 20 kD
Coomassie staining
Fig. Sup 1h
Purified SHP2
250 kD
150 kD
100 kD
 75 kD
W: SHP2
 50 kD

## Slide 31
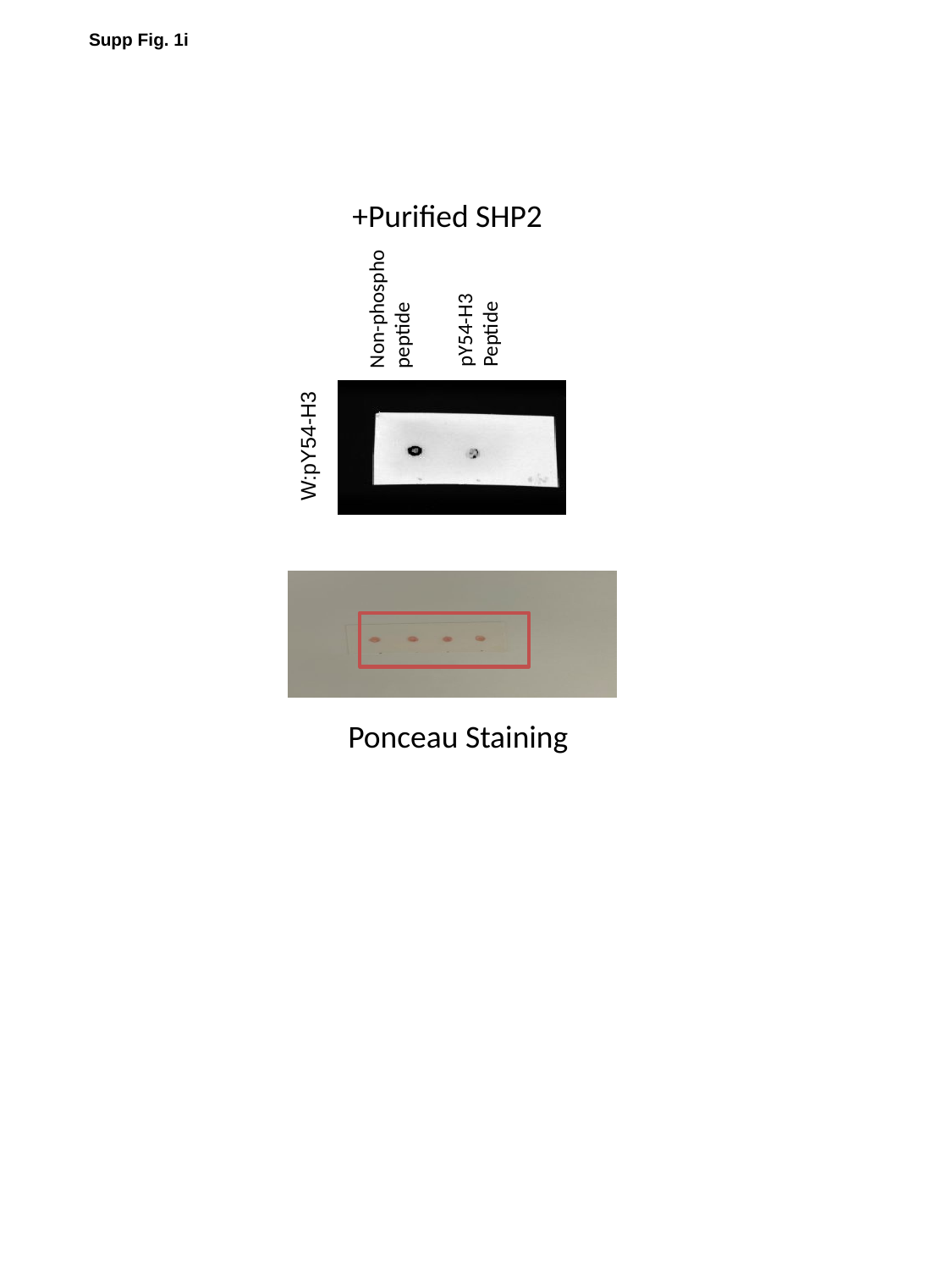

Supp Fig. 1i
+Purified SHP2
pY54-H3
Peptide
Non-phospho
peptide
W:pY54-H3
Ponceau Staining

## Slide 32
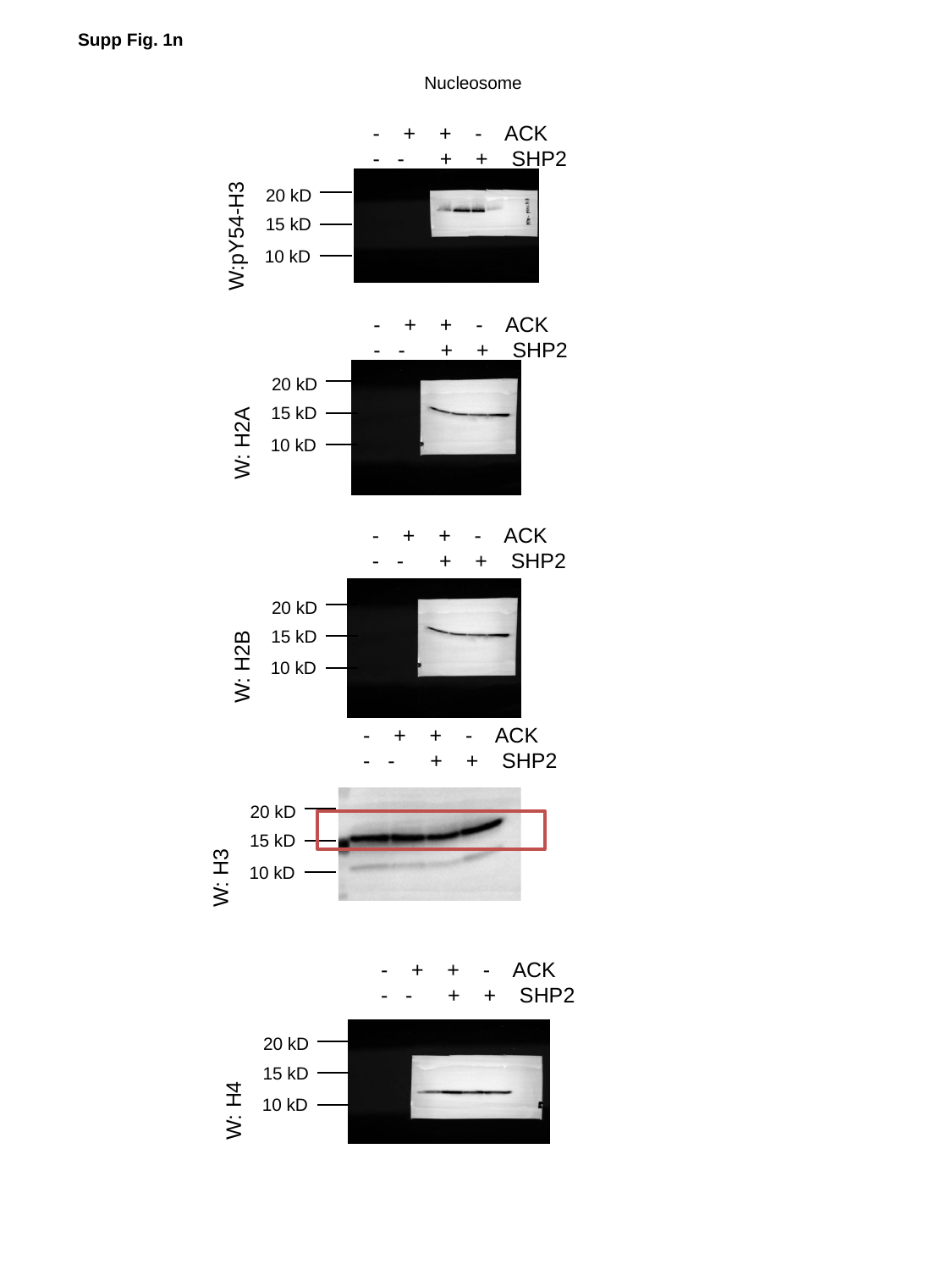

Supp Fig. 1n
Nucleosome
 - + + - ACK
 - - + + SHP2
 20 kD
W:pY54-H3
 15 kD
 10 kD
 - + + - ACK
 - - + + SHP2
 20 kD
W: H2A
 15 kD
 10 kD
 - + + - ACK
 - - + + SHP2
 20 kD
W: H2B
 15 kD
 10 kD
 - + + - ACK
 - - + + SHP2
 20 kD
W: H3
 15 kD
 10 kD
 - + + - ACK
 - - + + SHP2
 20 kD
W: H4
 15 kD
 10 kD

## Slide 33
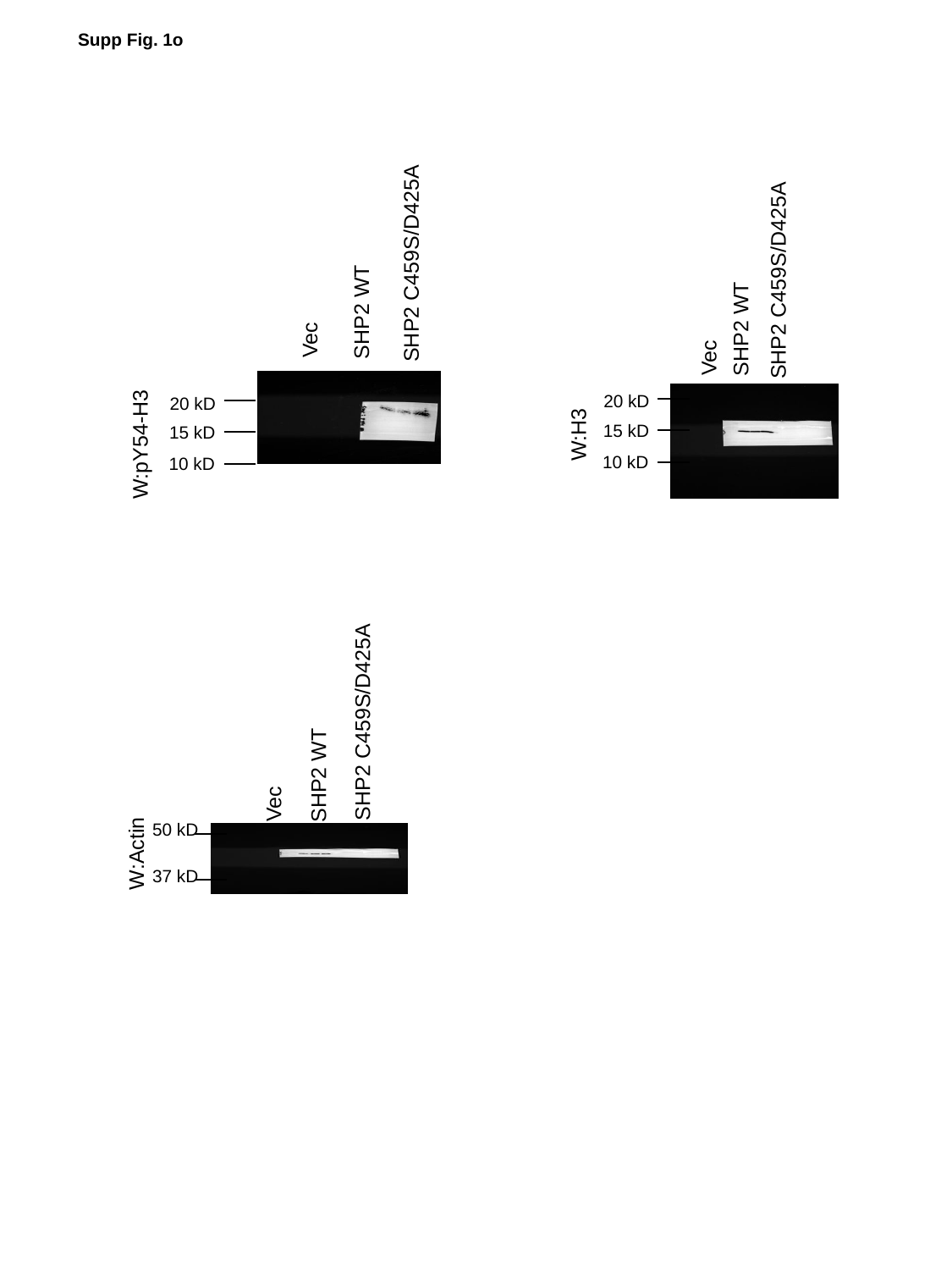

Supp Fig. 1o
SHP2 C459S/D425A
SHP2 C459S/D425A
Vec
SHP2 WT
SHP2 WT
Vec
W:H3
 20 kD
 20 kD
W:pY54-H3
 15 kD
 15 kD
 10 kD
 10 kD
SHP2 C459S/D425A
SHP2 WT
Vec
W:Actin
 50 kD
 37 kD

## Slide 34
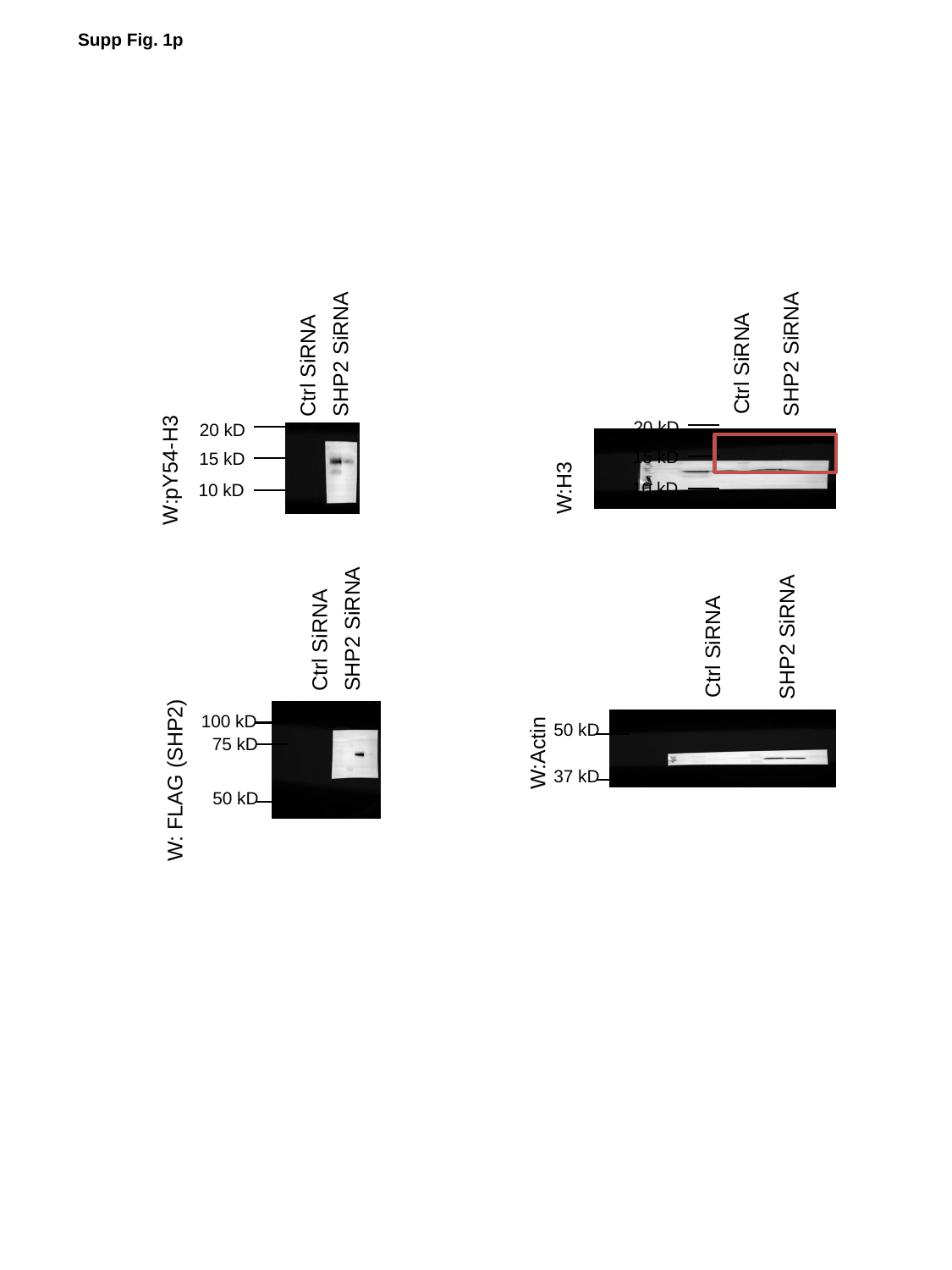

Supp Fig. 1p
Ctrl SiRNA
SHP2 SiRNA
SHP2 SiRNA
Ctrl SiRNA
 20 kD
 20 kD
W:H3
W:pY54-H3
 15 kD
 15 kD
 10 kD
 10 kD
SHP2 SiRNA
Ctrl SiRNA
Ctrl SiRNA
SHP2 SiRNA
 100 kD
W:Actin
 50 kD
 75 kD
W: FLAG (SHP2)
 37 kD
 50 kD

## Slide 35
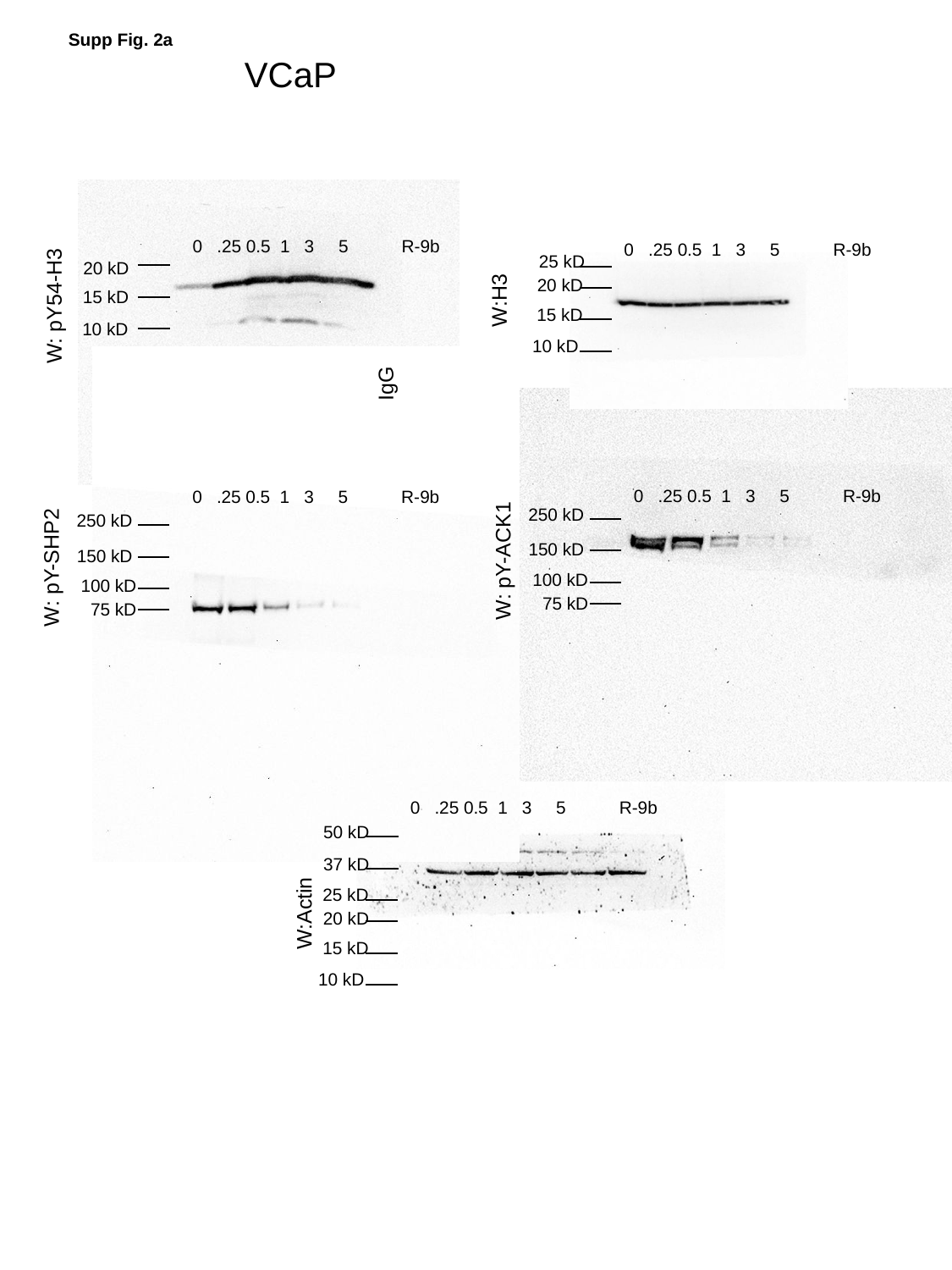

Supp Fig. 2a
VCaP
0 .25 0.5 1 3 5 R-9b
0 .25 0.5 1 3 5 R-9b
W:H3
 25 kD
 20 kD
 20 kD
W: pY54-H3
 15 kD
 15 kD
 10 kD
 10 kD
IgG
0 .25 0.5 1 3 5 R-9b
0 .25 0.5 1 3 5 R-9b
250 kD
250 kD
W: pY-ACK1
W: pY-SHP2
150 kD
150 kD
 100 kD
 100 kD
 75 kD
 75 kD
0 .25 0.5 1 3 5 R-9b
 50 kD
 37 kD
W:Actin
 25 kD
 20 kD
 15 kD
 10 kD

## Slide 36
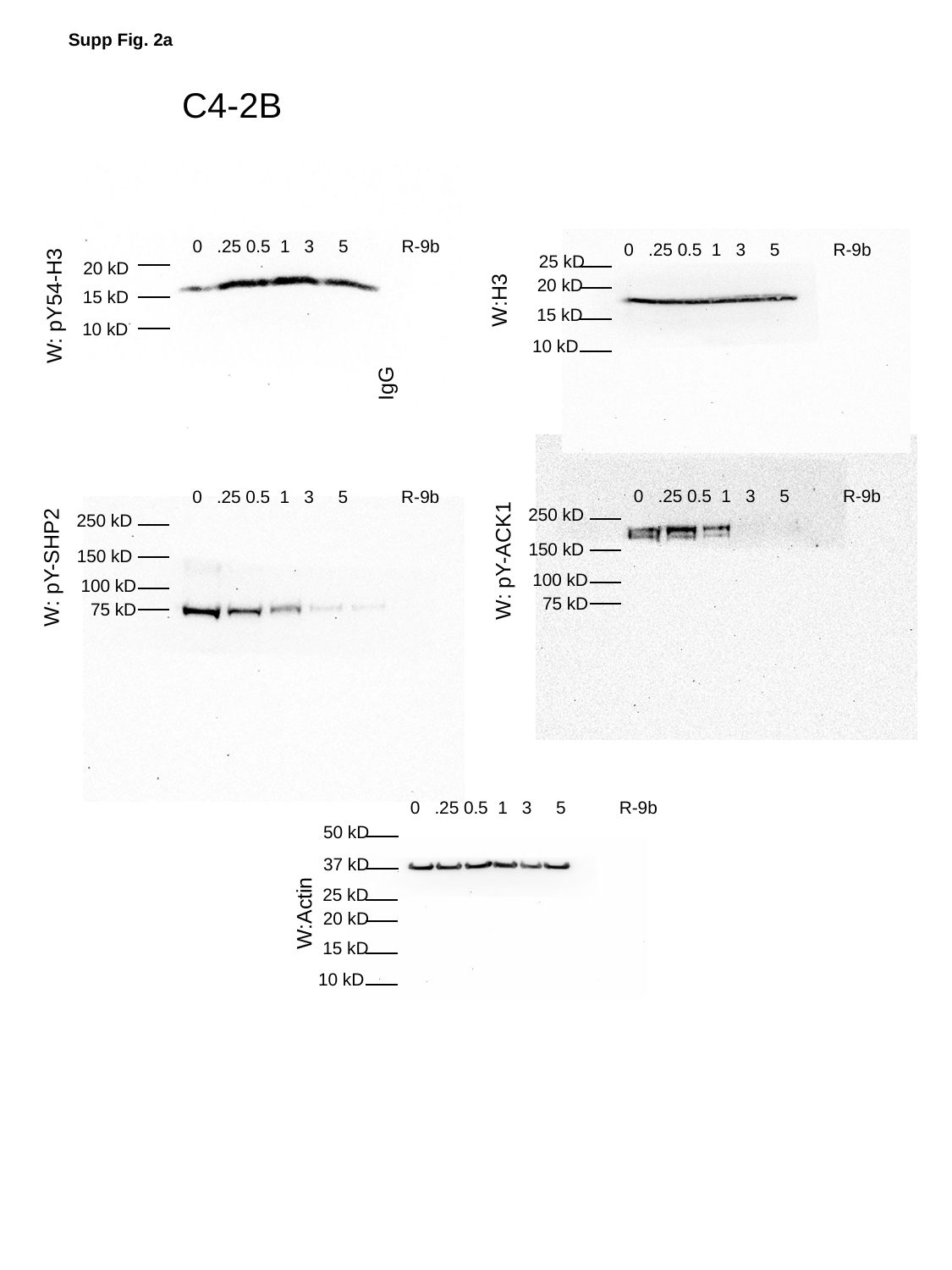

Supp Fig. 2a
C4-2B
0 .25 0.5 1 3 5 R-9b
0 .25 0.5 1 3 5 R-9b
W:H3
 25 kD
 20 kD
 20 kD
W: pY54-H3
 15 kD
 15 kD
 10 kD
 10 kD
IgG
0 .25 0.5 1 3 5 R-9b
0 .25 0.5 1 3 5 R-9b
250 kD
250 kD
W: pY-ACK1
W: pY-SHP2
150 kD
150 kD
 100 kD
 100 kD
 75 kD
 75 kD
0 .25 0.5 1 3 5 R-9b
 50 kD
 37 kD
W:Actin
 25 kD
 20 kD
 15 kD
 10 kD

## Slide 37
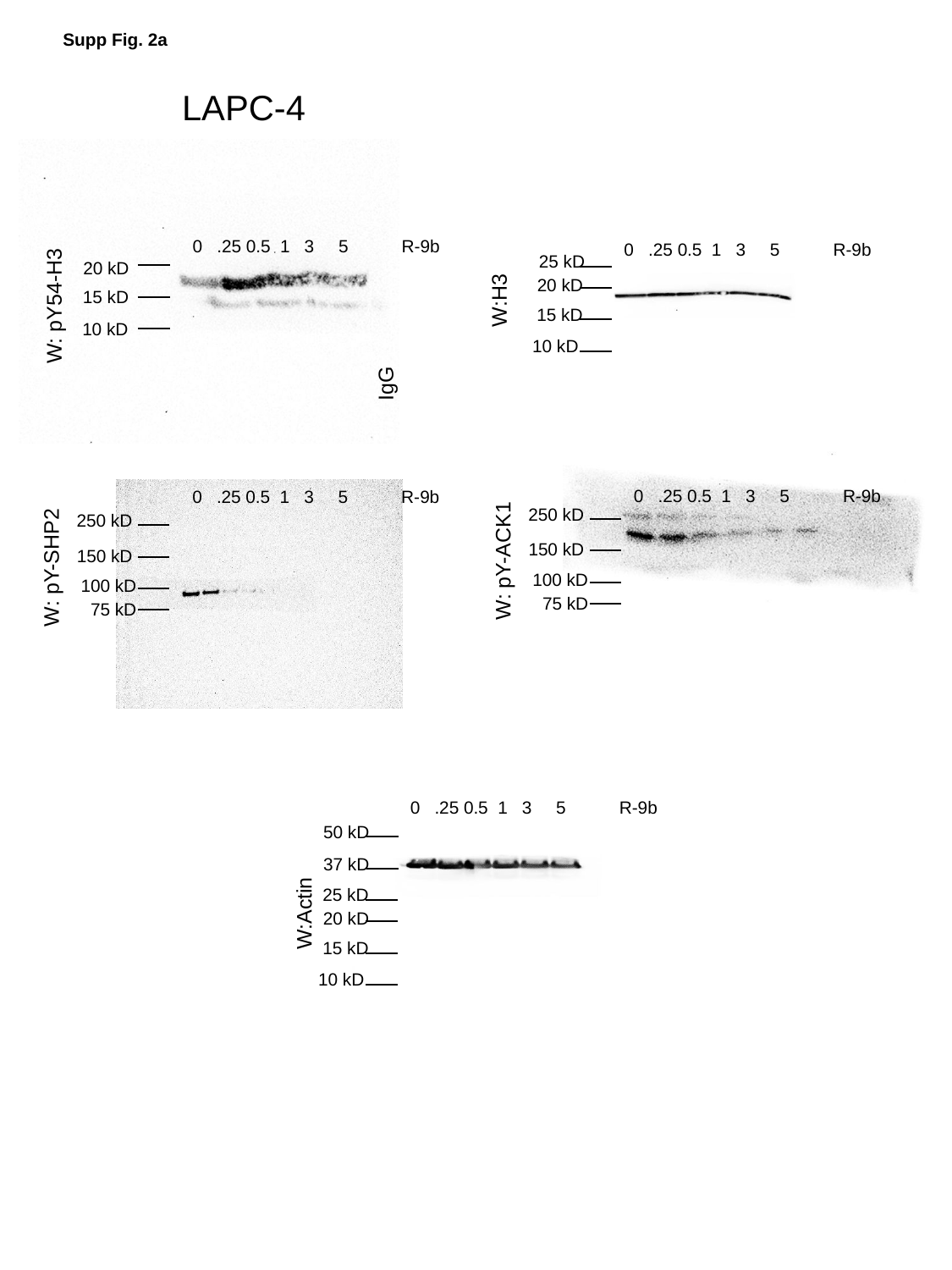

Supp Fig. 2a
LAPC-4
0 .25 0.5 1 3 5 R-9b
0 .25 0.5 1 3 5 R-9b
W:H3
 25 kD
 20 kD
 20 kD
W: pY54-H3
 15 kD
 15 kD
 10 kD
 10 kD
IgG
0 .25 0.5 1 3 5 R-9b
0 .25 0.5 1 3 5 R-9b
250 kD
250 kD
W: pY-ACK1
W: pY-SHP2
150 kD
150 kD
 100 kD
 100 kD
 75 kD
 75 kD
0 .25 0.5 1 3 5 R-9b
 50 kD
 37 kD
W:Actin
 25 kD
 20 kD
 15 kD
 10 kD

## Slide 38
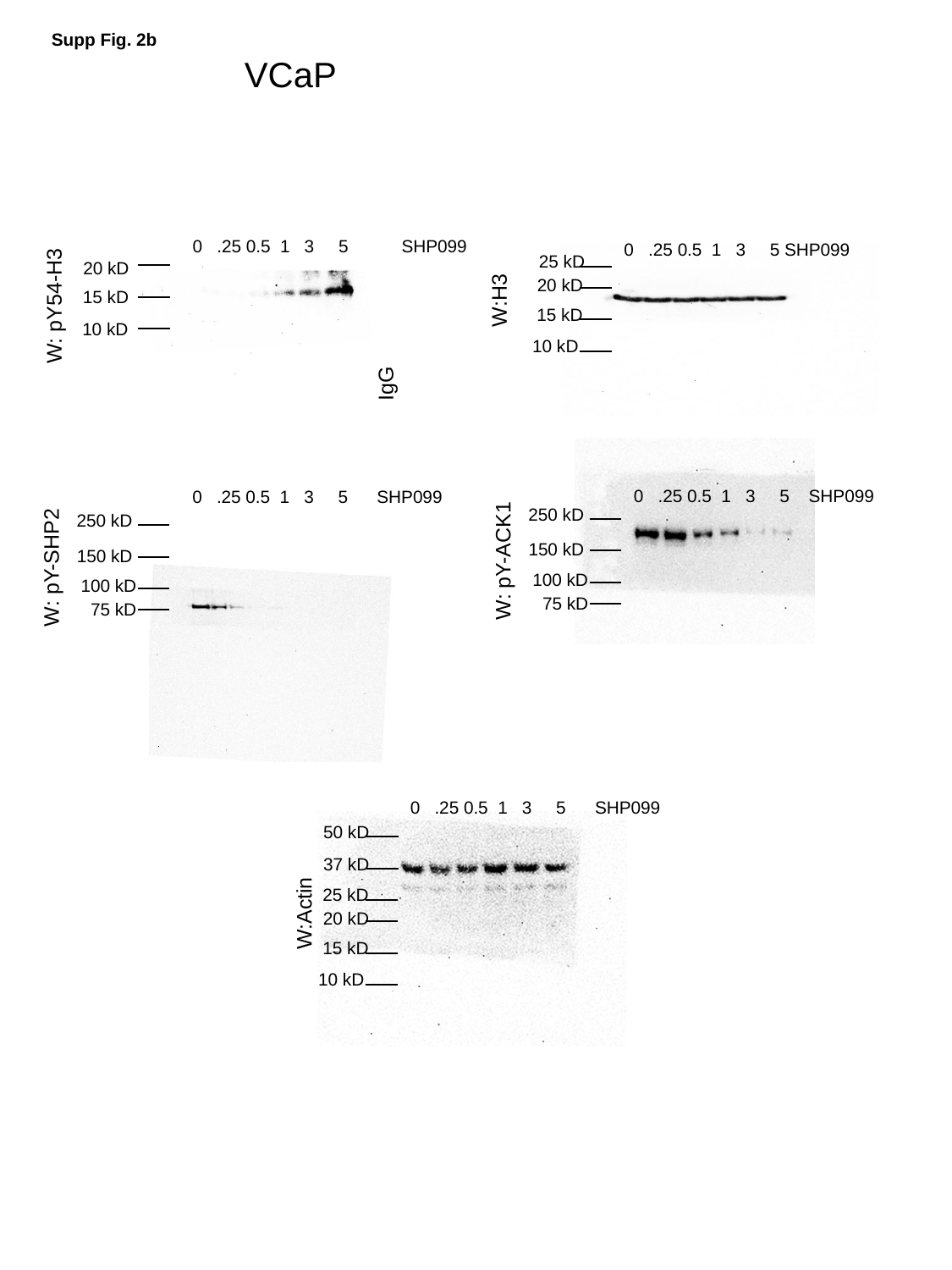

Supp Fig. 2b
VCaP
0 .25 0.5 1 3 5 SHP099
0 .25 0.5 1 3 5 SHP099
W:H3
 25 kD
 20 kD
 20 kD
W: pY54-H3
 15 kD
 15 kD
 10 kD
 10 kD
IgG
0 .25 0.5 1 3 5 SHP099
0 .25 0.5 1 3 5 SHP099
250 kD
250 kD
W: pY-ACK1
W: pY-SHP2
150 kD
150 kD
 100 kD
 100 kD
 75 kD
 75 kD
0 .25 0.5 1 3 5 SHP099
 50 kD
 37 kD
W:Actin
 25 kD
 20 kD
 15 kD
 10 kD

## Slide 39
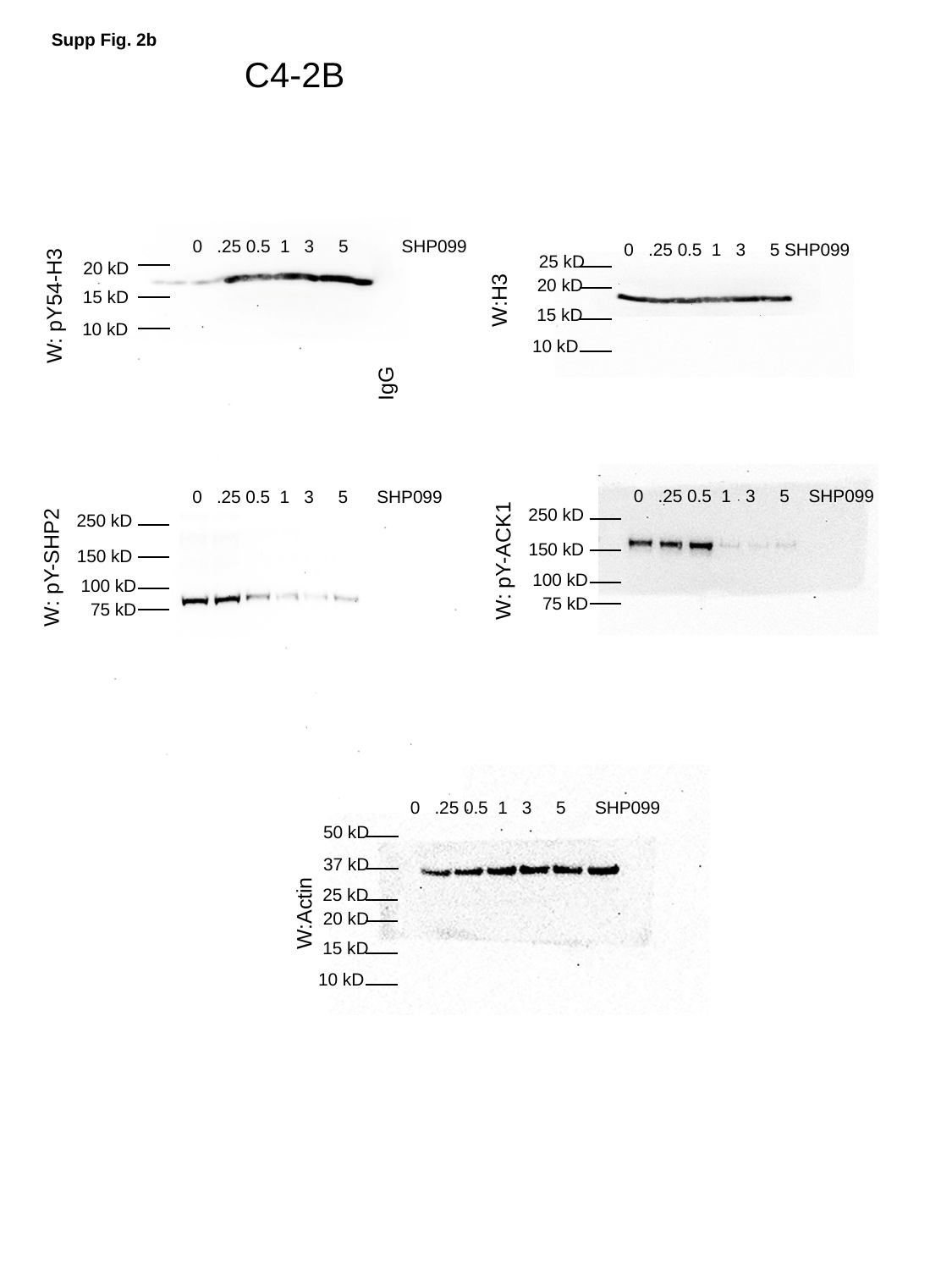

Supp Fig. 2b
C4-2B
0 .25 0.5 1 3 5 SHP099
0 .25 0.5 1 3 5 SHP099
W:H3
 25 kD
 20 kD
 20 kD
W: pY54-H3
 15 kD
 15 kD
 10 kD
 10 kD
IgG
0 .25 0.5 1 3 5 SHP099
0 .25 0.5 1 3 5 SHP099
250 kD
250 kD
W: pY-ACK1
W: pY-SHP2
150 kD
150 kD
 100 kD
 100 kD
 75 kD
 75 kD
0 .25 0.5 1 3 5 SHP099
 50 kD
 37 kD
W:Actin
 25 kD
 20 kD
 15 kD
 10 kD

## Slide 40
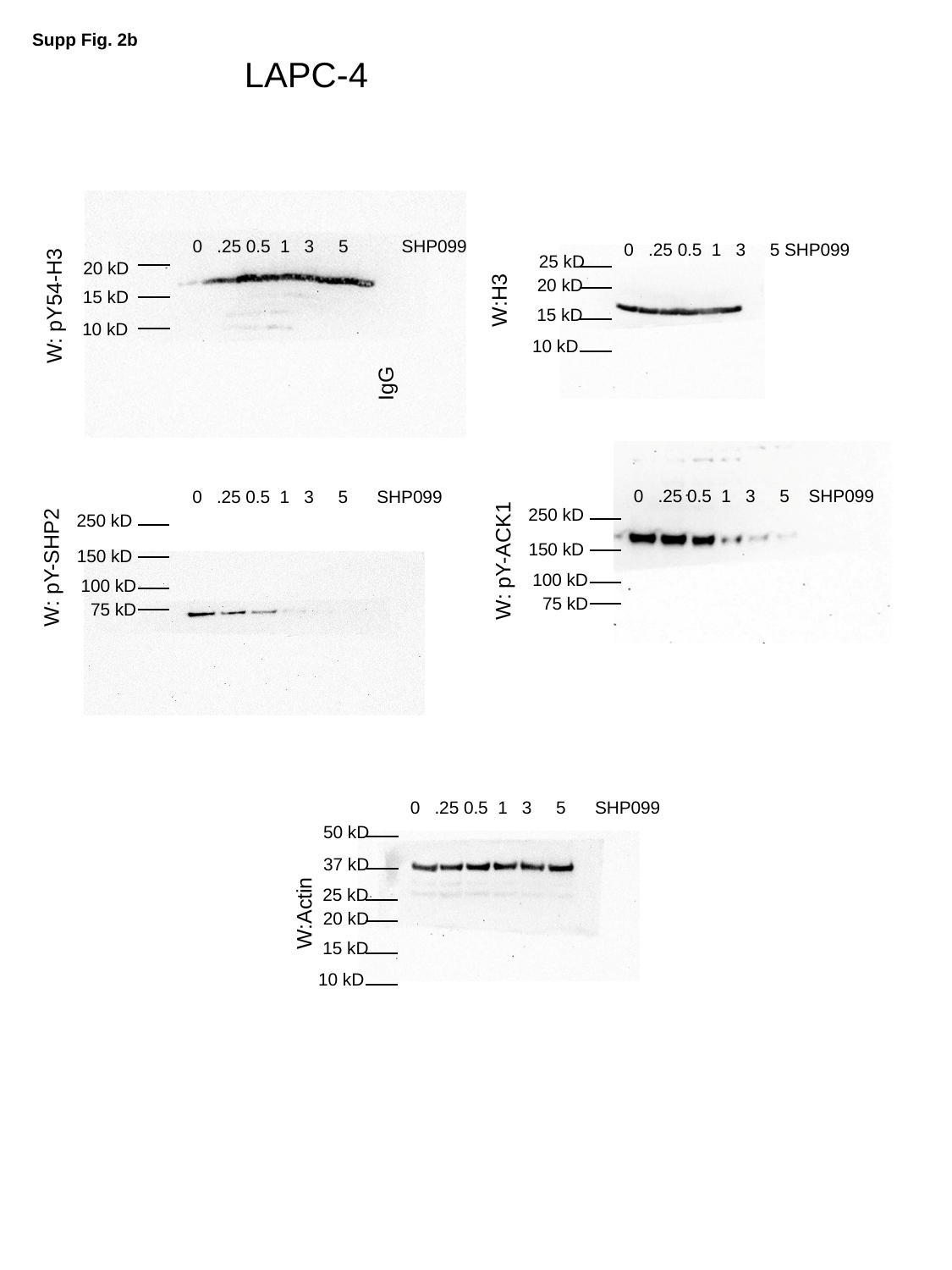

Supp Fig. 2b
LAPC-4
0 .25 0.5 1 3 5 SHP099
0 .25 0.5 1 3 5 SHP099
W:H3
 25 kD
 20 kD
 20 kD
W: pY54-H3
 15 kD
 15 kD
 10 kD
 10 kD
IgG
0 .25 0.5 1 3 5 SHP099
0 .25 0.5 1 3 5 SHP099
250 kD
250 kD
W: pY-ACK1
W: pY-SHP2
150 kD
150 kD
 100 kD
 100 kD
 75 kD
 75 kD
0 .25 0.5 1 3 5 SHP099
 50 kD
 37 kD
W:Actin
 25 kD
 20 kD
 15 kD
 10 kD

## Slide 41
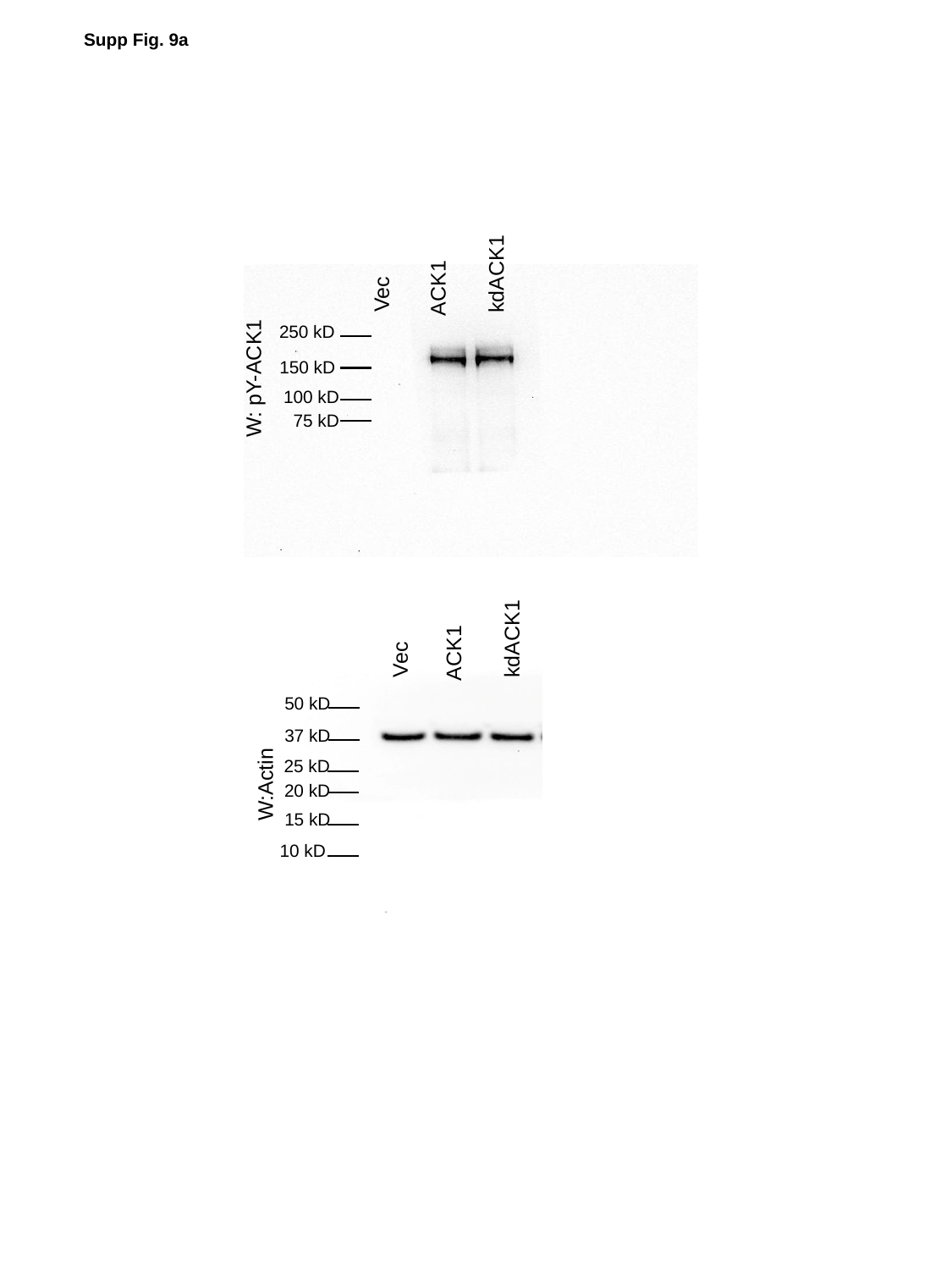

Supp Fig. 9a
kdACK1
ACK1
Vec
250 kD
W: pY-ACK1
150 kD
 100 kD
 75 kD
kdACK1
ACK1
Vec
 50 kD
 37 kD
W:Actin
 25 kD
 20 kD
 15 kD
 10 kD

## Slide 42
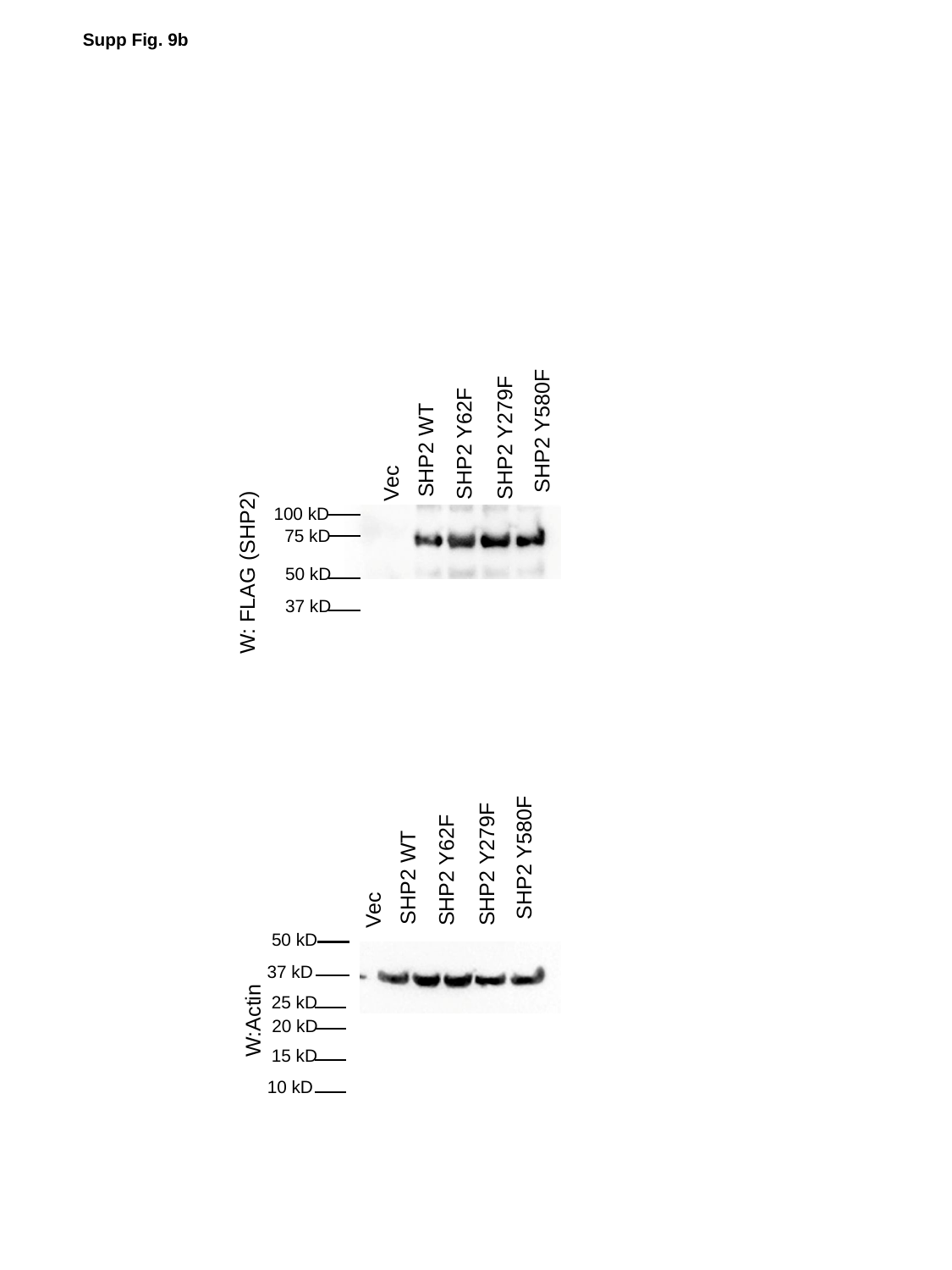

Supp Fig. 9b
SHP2 Y580F
SHP2 Y279F
SHP2 Y62F
SHP2 WT
Vec
 100 kD
 75 kD
W: FLAG (SHP2)
 50 kD
 37 kD
SHP2 Y580F
SHP2 Y279F
SHP2 Y62F
SHP2 WT
Vec
 50 kD
 37 kD
W:Actin
 25 kD
 20 kD
 15 kD
 10 kD

## Slide 43
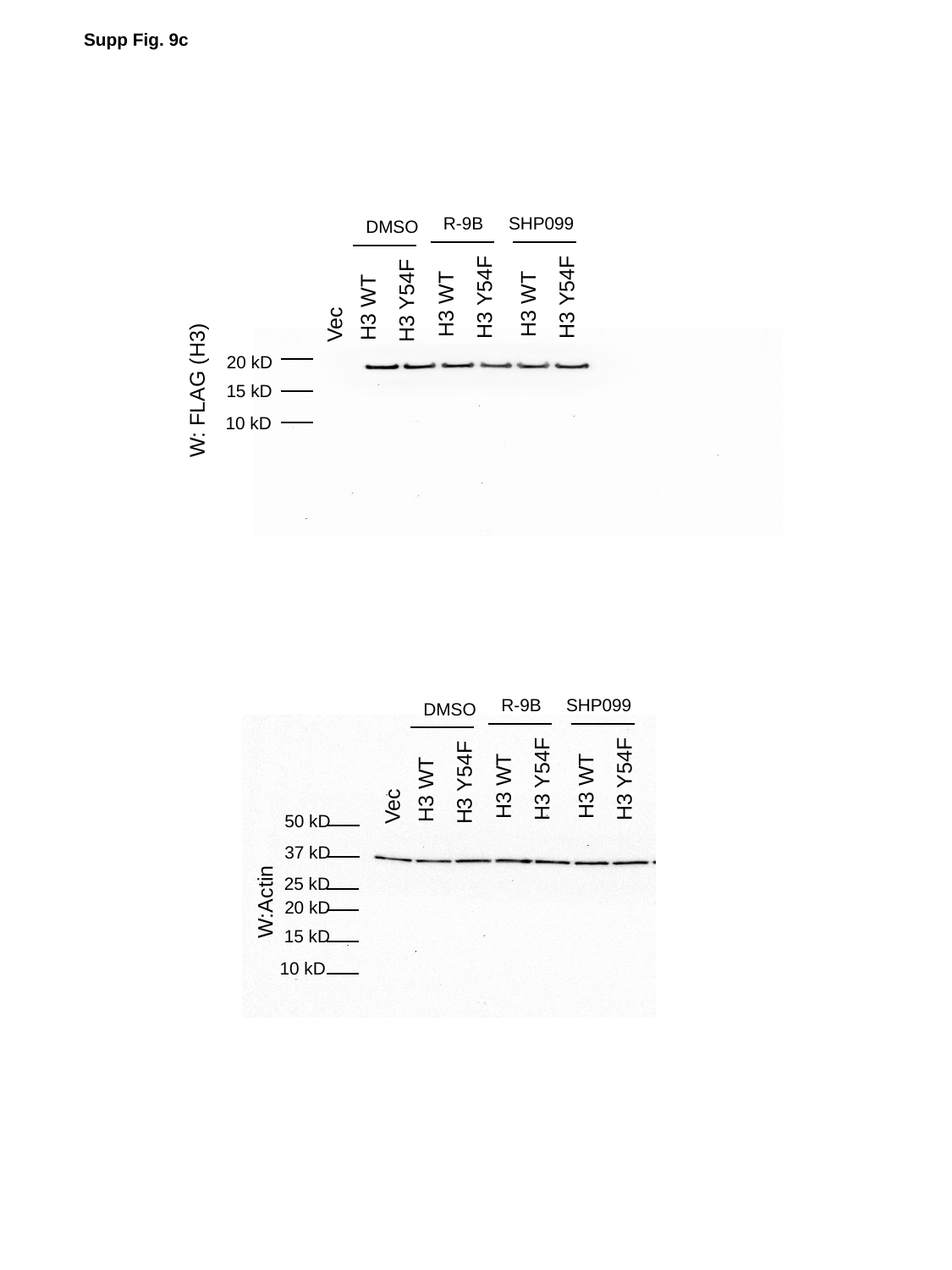

Supp Fig. 9c
SHP099
R-9B
DMSO
H3 Y54F
H3 Y54F
H3 Y54F
H3 WT
H3 WT
H3 WT
Vec
 20 kD
W: FLAG (H3)
 15 kD
 10 kD
SHP099
R-9B
DMSO
H3 Y54F
H3 Y54F
H3 Y54F
H3 WT
H3 WT
H3 WT
Vec
 50 kD
 37 kD
W:Actin
 25 kD
 20 kD
 15 kD
 10 kD

## Slide 44
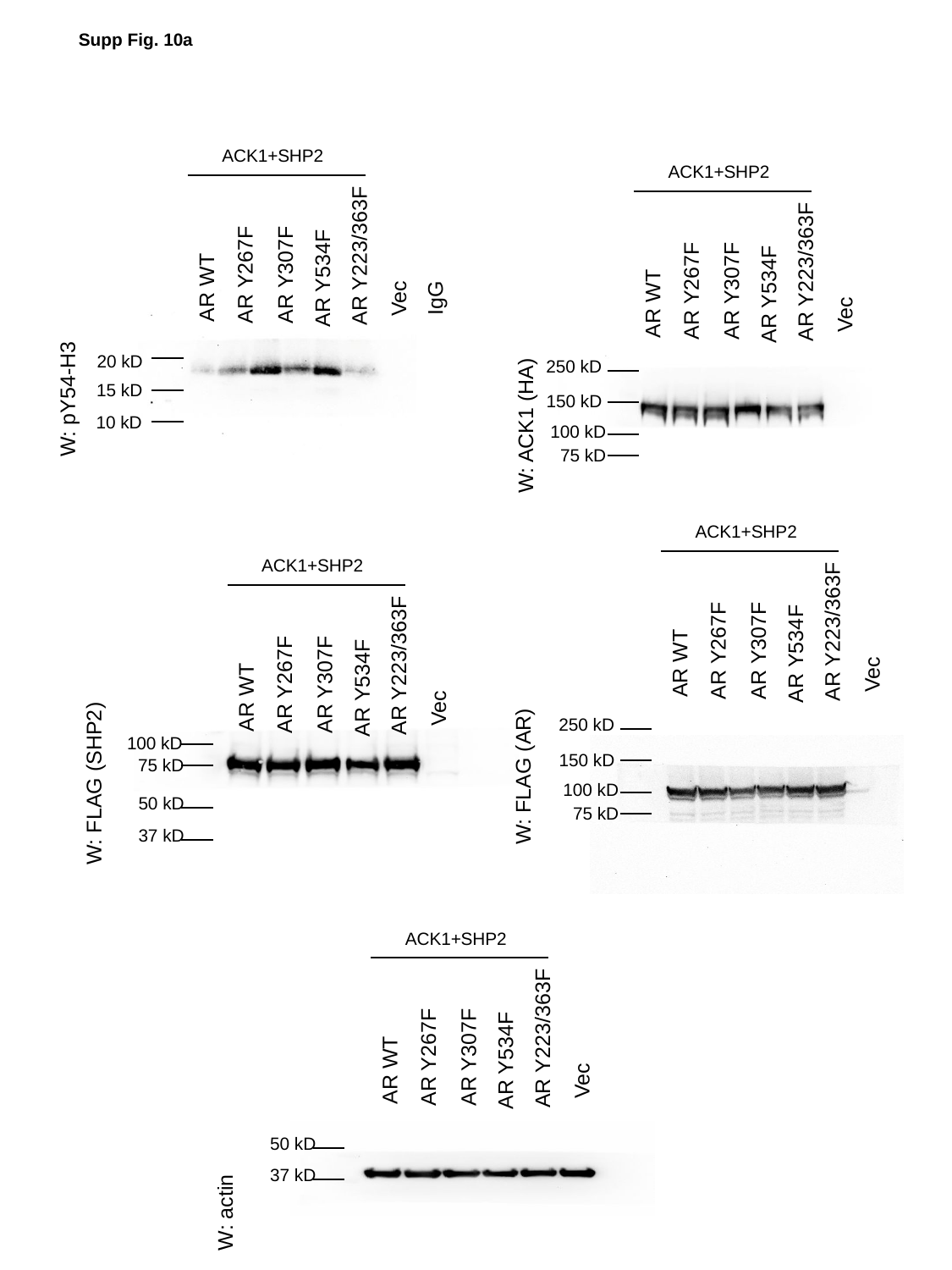

Supp Fig. 10a
ACK1+SHP2
ACK1+SHP2
AR Y223/363F
AR Y223/363F
AR Y307F
AR Y534F
AR Y267F
AR WT
AR Y307F
AR Y534F
AR Y267F
IgG
Vec
AR WT
Vec
 20 kD
250 kD
W: pY54-H3
 15 kD
150 kD
W: ACK1 (HA)
 10 kD
 100 kD
 75 kD
ACK1+SHP2
ACK1+SHP2
AR Y223/363F
AR Y307F
AR Y534F
AR Y223/363F
AR Y267F
AR WT
Vec
AR Y307F
AR Y534F
AR Y267F
AR WT
Vec
250 kD
 100 kD
W: FLAG (AR)
150 kD
 75 kD
W: FLAG (SHP2)
 100 kD
 50 kD
 75 kD
 37 kD
ACK1+SHP2
AR Y223/363F
AR Y307F
AR Y534F
AR Y267F
AR WT
Vec
 50 kD
 37 kD
W: actin

## Slide 45
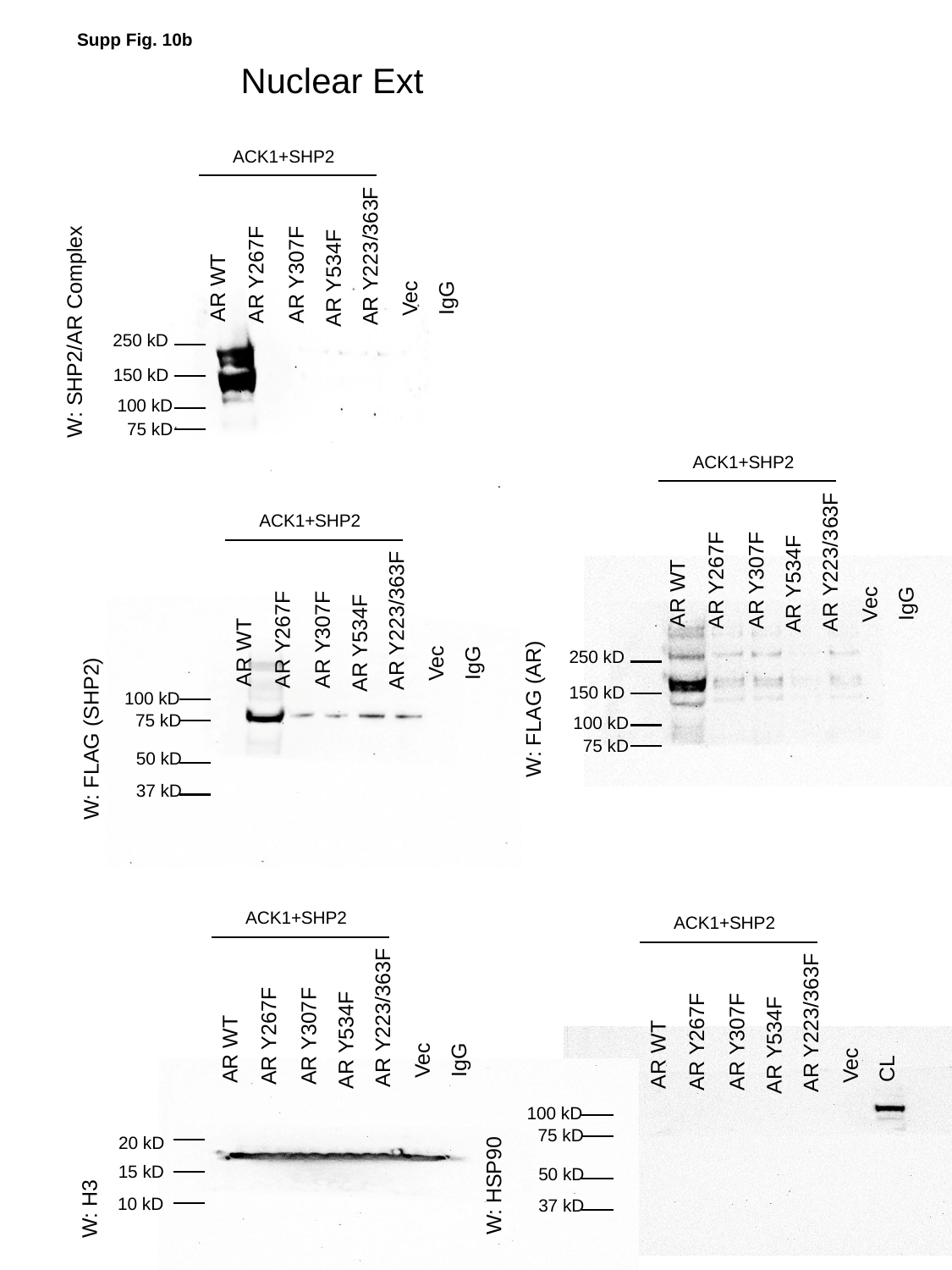

Supp Fig. 10b
Nuclear Ext
ACK1+SHP2
AR Y223/363F
AR Y307F
AR Y534F
AR Y267F
AR WT
IgG
Vec
W: SHP2/AR Complex
250 kD
150 kD
 100 kD
 75 kD
ACK1+SHP2
ACK1+SHP2
AR Y223/363F
AR Y307F
AR Y534F
AR Y267F
AR WT
IgG
Vec
AR Y223/363F
AR Y307F
AR Y534F
AR Y267F
AR WT
IgG
Vec
250 kD
W: FLAG (AR)
150 kD
 100 kD
 75 kD
 100 kD
W: FLAG (SHP2)
 75 kD
 50 kD
 37 kD
ACK1+SHP2
ACK1+SHP2
AR Y223/363F
AR Y223/363F
AR Y307F
AR Y534F
AR Y307F
AR Y267F
AR Y534F
AR Y267F
AR WT
AR WT
IgG
Vec
CL
Vec
 100 kD
 75 kD
 20 kD
W: HSP90
W: H3
 15 kD
 50 kD
 10 kD
 37 kD

## Slide 46
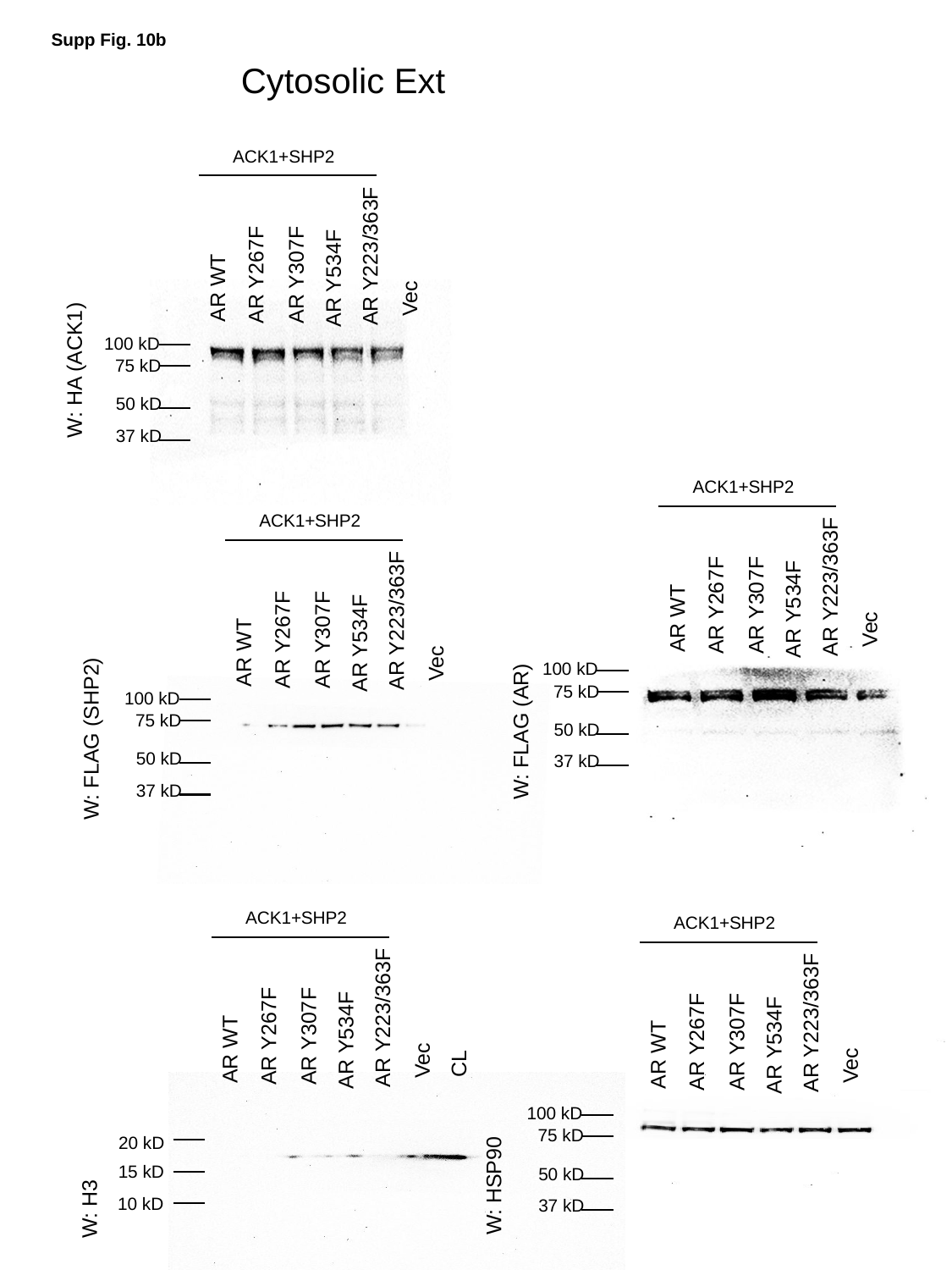

Supp Fig. 10b
Cytosolic Ext
ACK1+SHP2
AR Y223/363F
AR Y307F
AR Y534F
AR Y267F
AR WT
Vec
W: HA (ACK1)
 100 kD
 75 kD
 50 kD
 37 kD
ACK1+SHP2
ACK1+SHP2
AR Y223/363F
AR Y307F
AR Y534F
AR Y223/363F
AR Y267F
AR WT
Vec
AR Y307F
AR Y534F
AR Y267F
AR WT
Vec
 100 kD
 75 kD
 100 kD
W: FLAG (AR)
 75 kD
W: FLAG (SHP2)
 50 kD
 50 kD
 37 kD
 37 kD
ACK1+SHP2
ACK1+SHP2
AR Y223/363F
AR Y223/363F
AR Y307F
AR Y534F
AR Y307F
AR Y267F
AR Y534F
AR Y267F
AR WT
AR WT
CL
Vec
Vec
 100 kD
 75 kD
 20 kD
W: HSP90
W: H3
 15 kD
 50 kD
 10 kD
 37 kD

## Slide 47
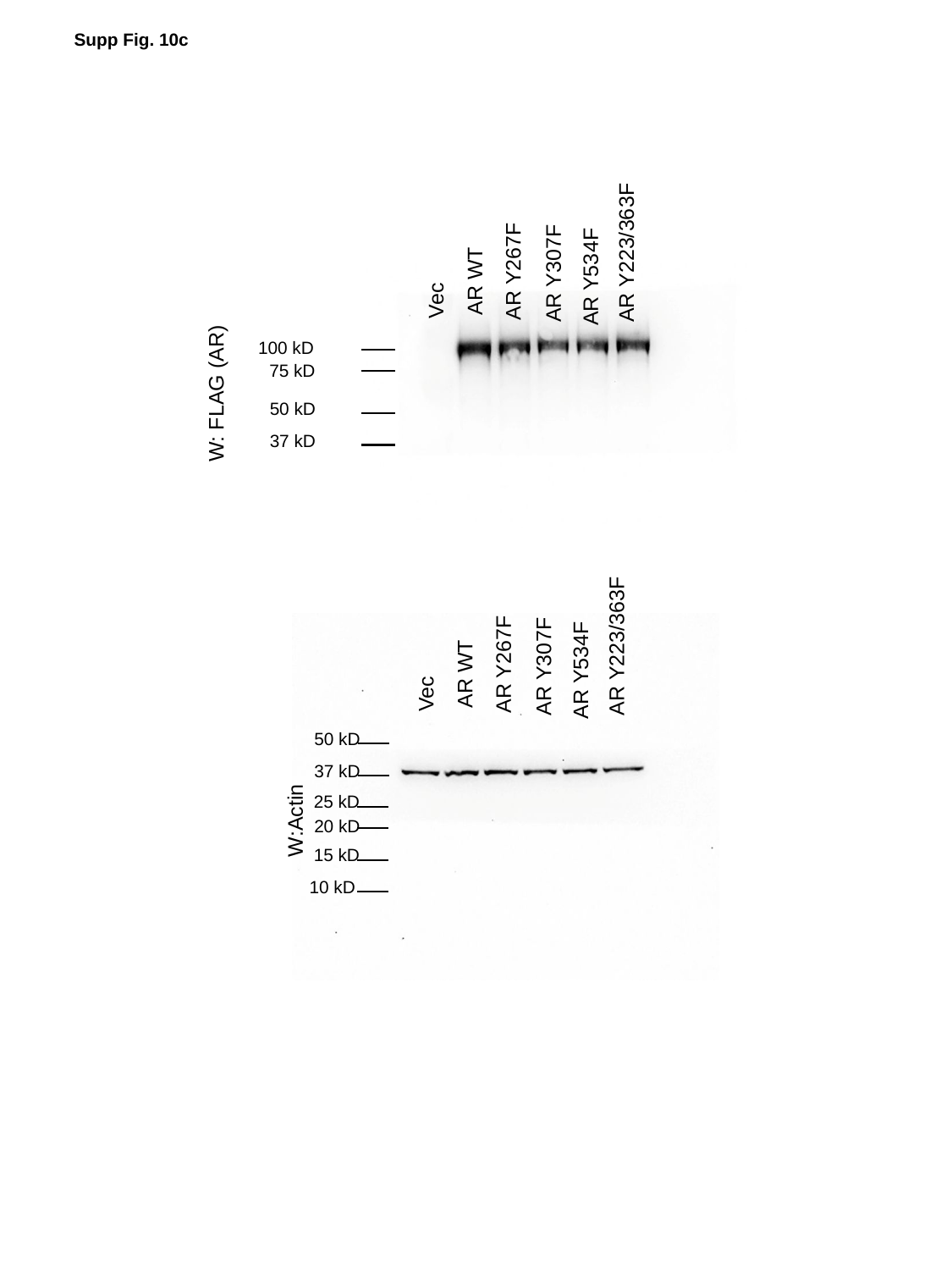

Supp Fig. 10c
AR Y223/363F
AR Y307F
AR Y534F
AR Y267F
AR WT
Vec
W: FLAG (AR)
 100 kD
 75 kD
 50 kD
 37 kD
AR Y223/363F
AR Y307F
AR Y534F
AR Y267F
AR WT
Vec
 50 kD
 37 kD
W:Actin
 25 kD
 20 kD
 15 kD
 10 kD

## Slide 48
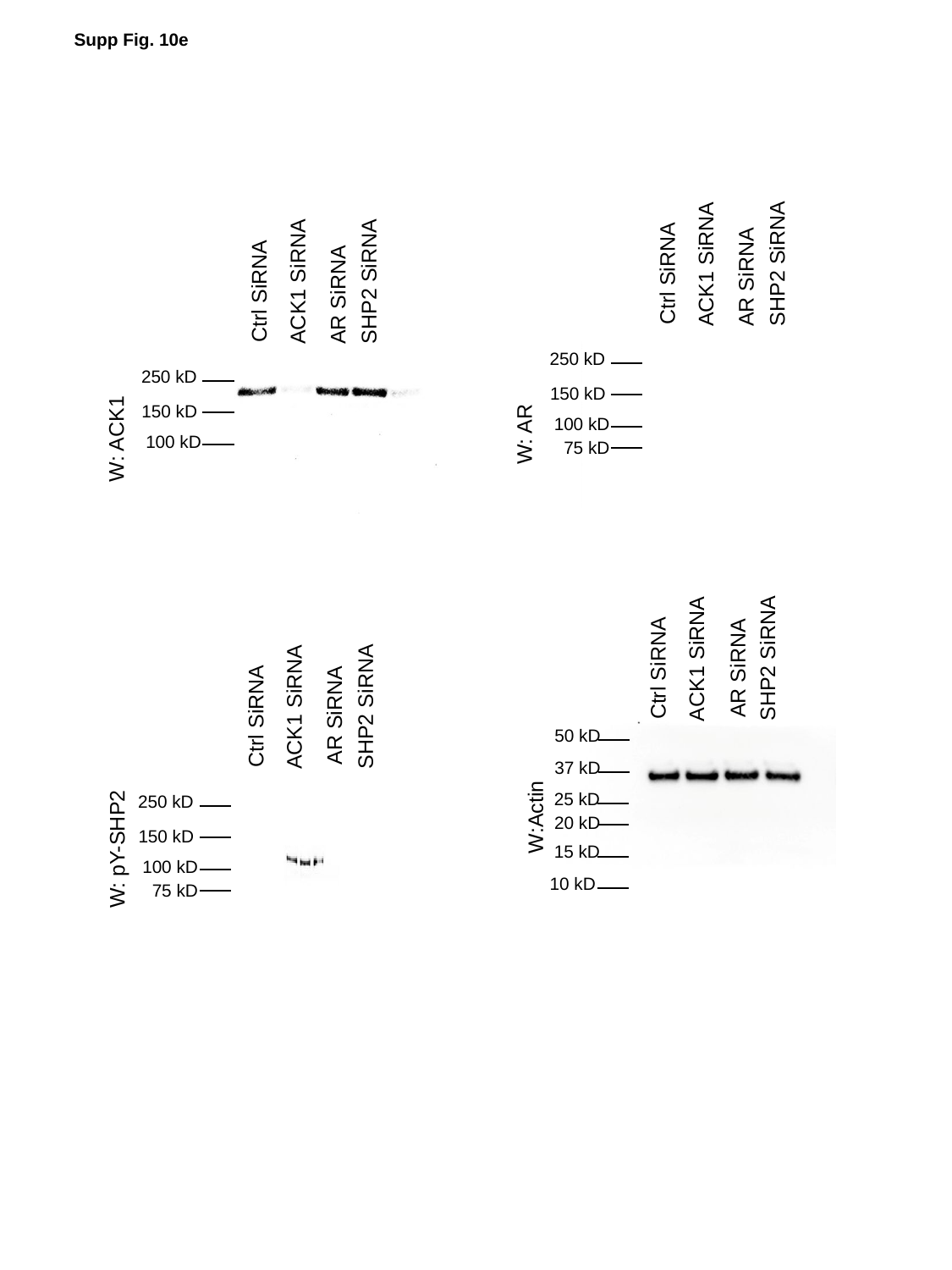

Supp Fig. 10e
AR SiRNA
Ctrl SiRNA
SHP2 SiRNA
ACK1 SiRNA
AR SiRNA
Ctrl SiRNA
SHP2 SiRNA
ACK1 SiRNA
250 kD
W: AR
250 kD
W: ACK1
150 kD
150 kD
 100 kD
 100 kD
 75 kD
AR SiRNA
Ctrl SiRNA
SHP2 SiRNA
ACK1 SiRNA
AR SiRNA
Ctrl SiRNA
SHP2 SiRNA
ACK1 SiRNA
 50 kD
 37 kD
W:Actin
 25 kD
250 kD
W: pY-SHP2
 20 kD
150 kD
 15 kD
 100 kD
 10 kD
 75 kD

## Slide 49
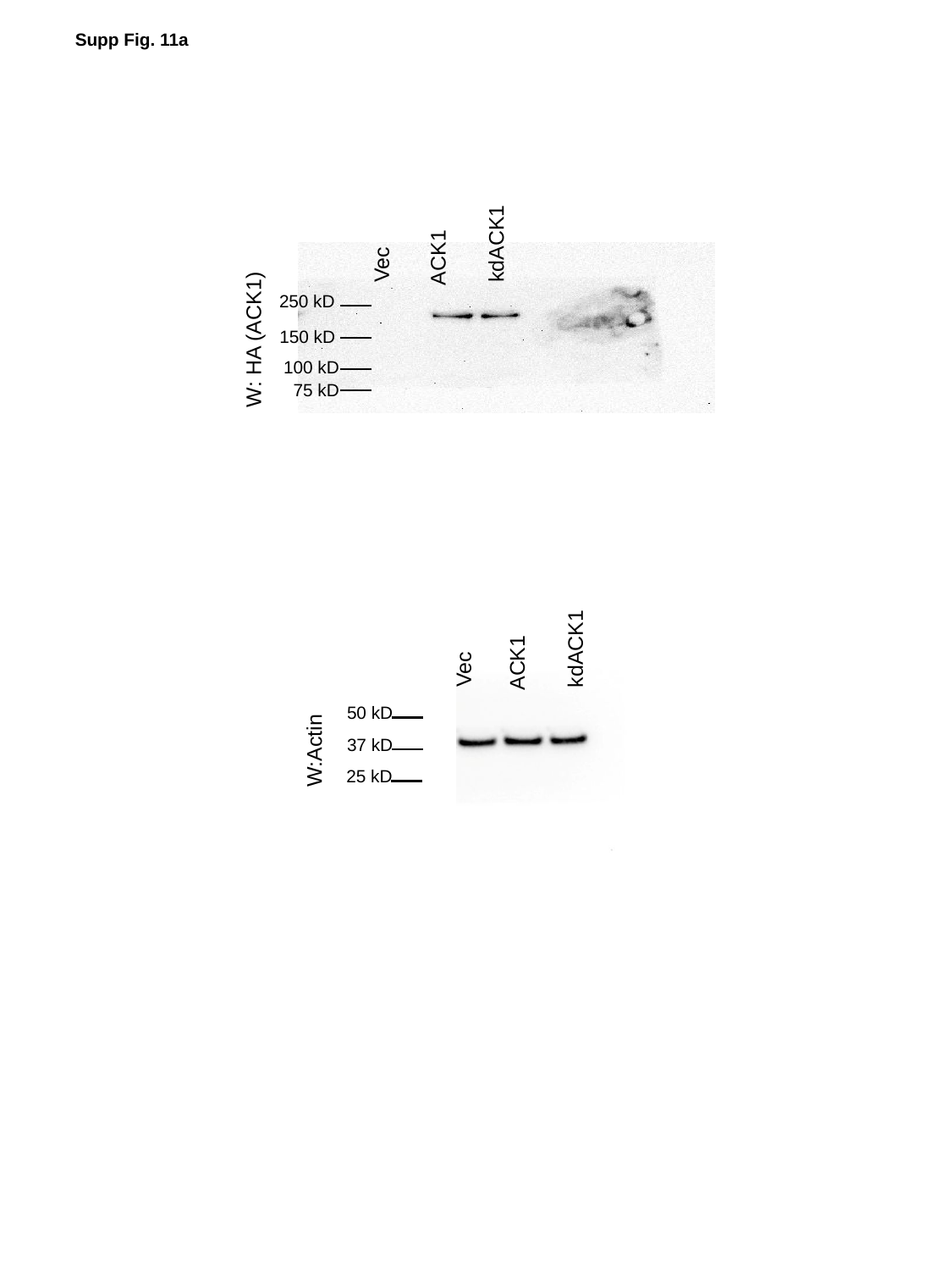

Supp Fig. 11a
kdACK1
ACK1
Vec
250 kD
W: HA (ACK1)
150 kD
 100 kD
 75 kD
kdACK1
ACK1
Vec
 50 kD
W:Actin
 37 kD
 25 kD

## Slide 50
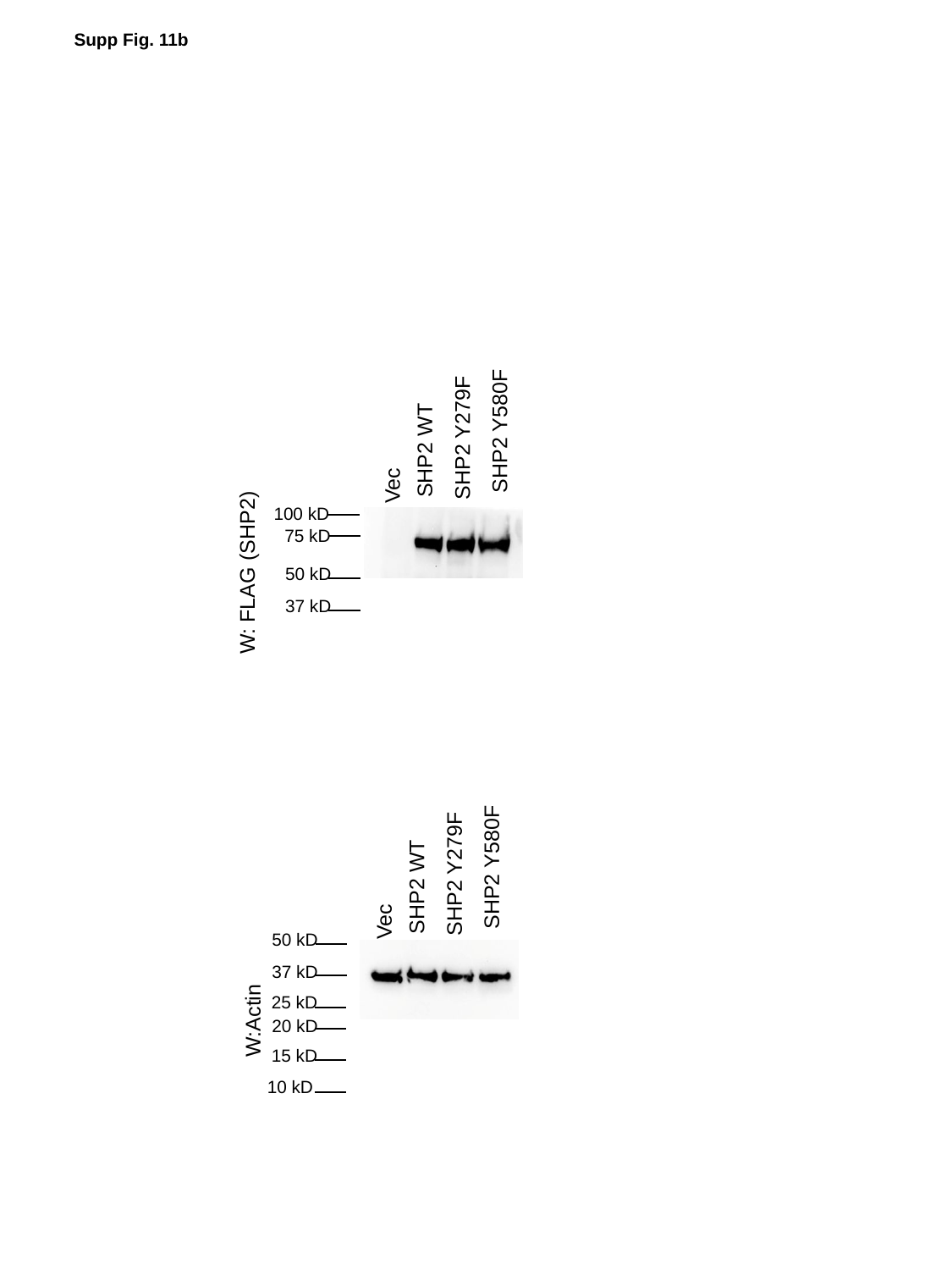

Supp Fig. 11b
SHP2 Y580F
SHP2 Y279F
SHP2 WT
Vec
 100 kD
 75 kD
W: FLAG (SHP2)
 50 kD
 37 kD
SHP2 Y580F
SHP2 Y279F
SHP2 WT
Vec
 50 kD
 37 kD
W:Actin
 25 kD
 20 kD
 15 kD
 10 kD

## Slide 51
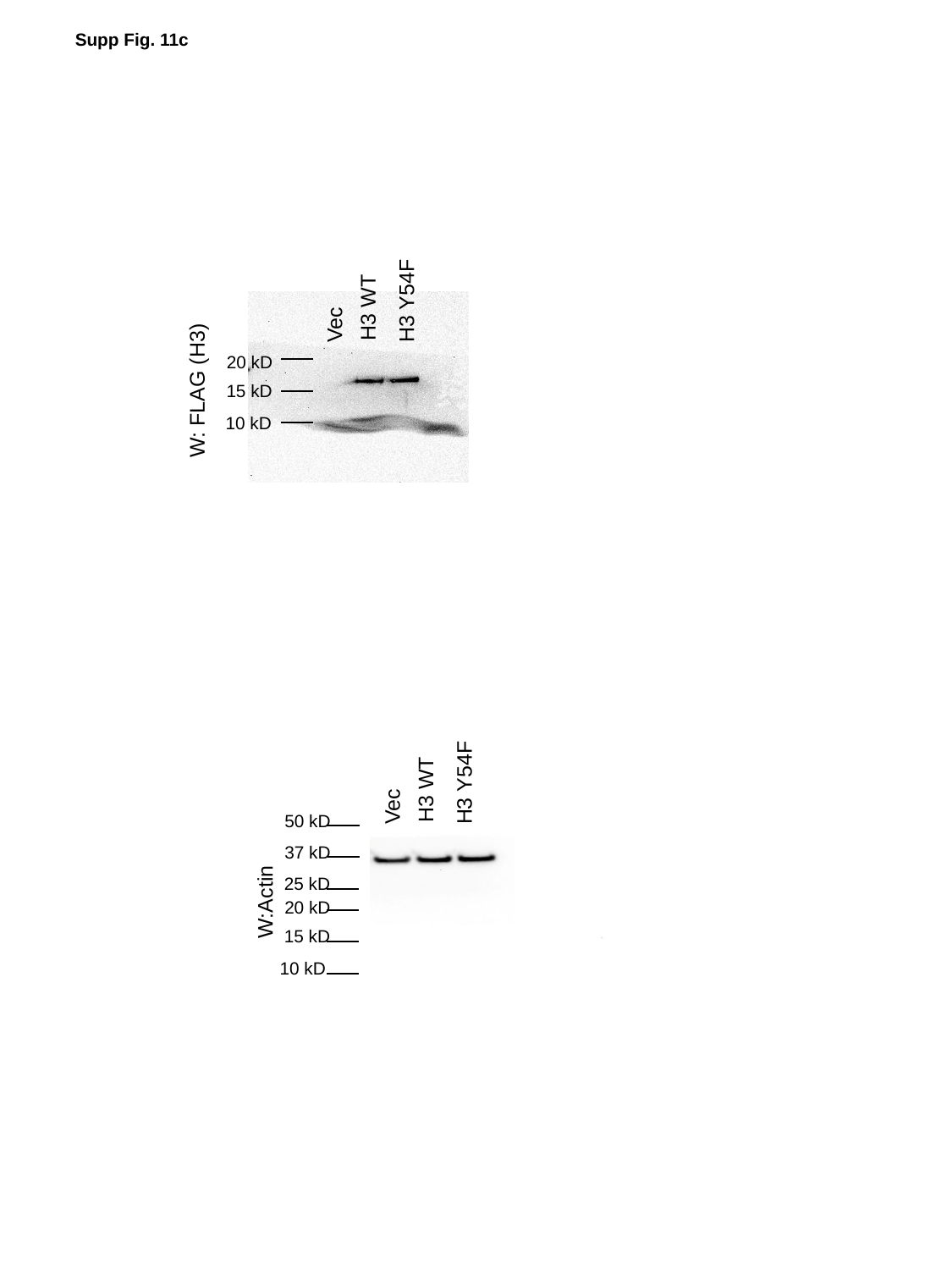

Supp Fig. 11c
H3 Y54F
H3 WT
Vec
 20 kD
W: FLAG (H3)
 15 kD
 10 kD
H3 Y54F
H3 WT
Vec
 50 kD
 37 kD
W:Actin
 25 kD
 20 kD
 15 kD
 10 kD

## Slide 52
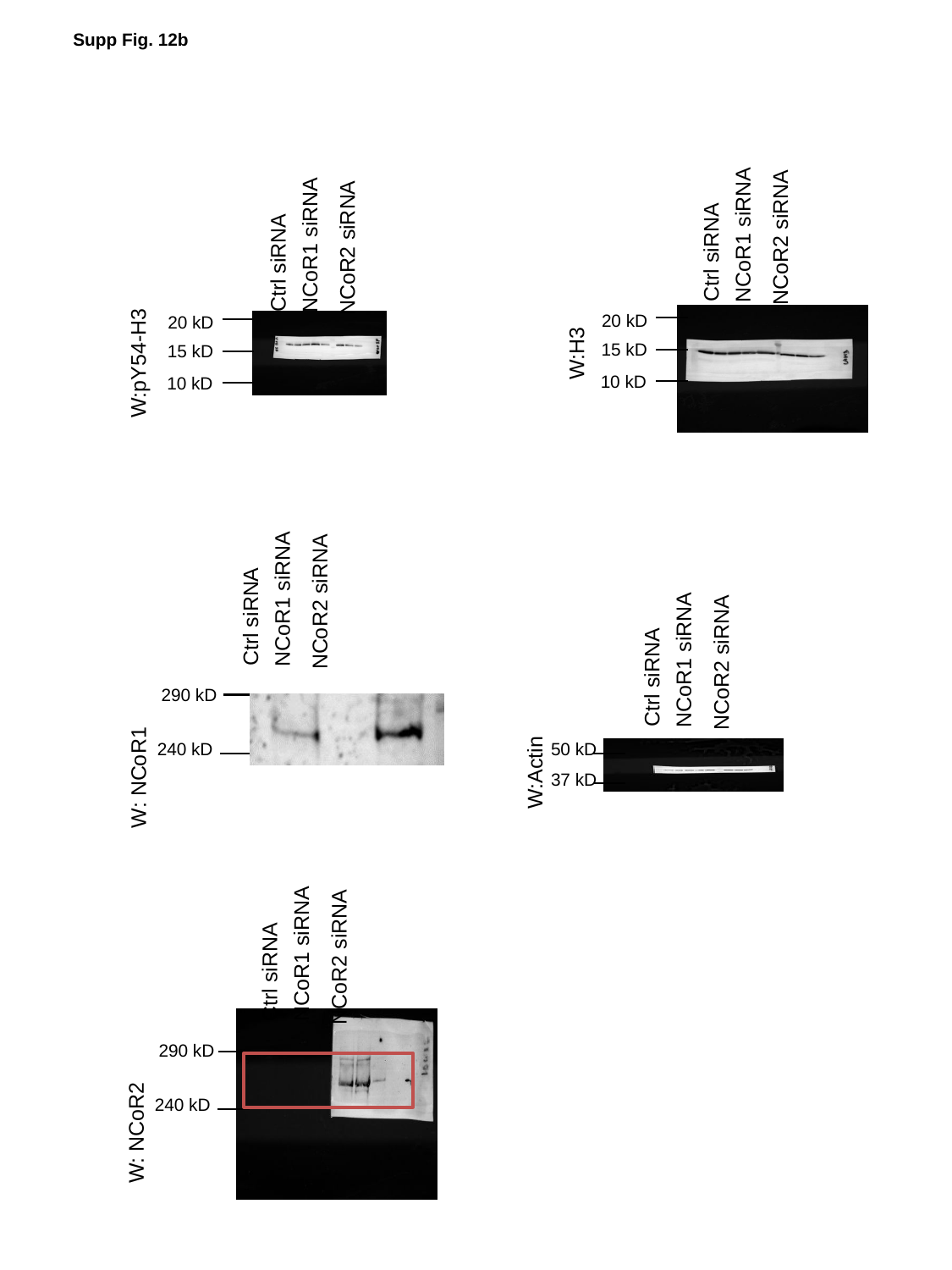

Supp Fig. 12b
NCoR2 siRNA
NCoR2 siRNA
NCoR1 siRNA
NCoR1 siRNA
Ctrl siRNA
Ctrl siRNA
W:H3
 20 kD
 20 kD
W:pY54-H3
 15 kD
 15 kD
 10 kD
 10 kD
NCoR2 siRNA
NCoR1 siRNA
Ctrl siRNA
NCoR2 siRNA
NCoR1 siRNA
Ctrl siRNA
290 kD
W: NCoR1
W:Actin
 50 kD
 240 kD
 37 kD
NCoR2 siRNA
NCoR1 siRNA
Ctrl siRNA
290 kD
W: NCoR2
 240 kD

## Slide 53
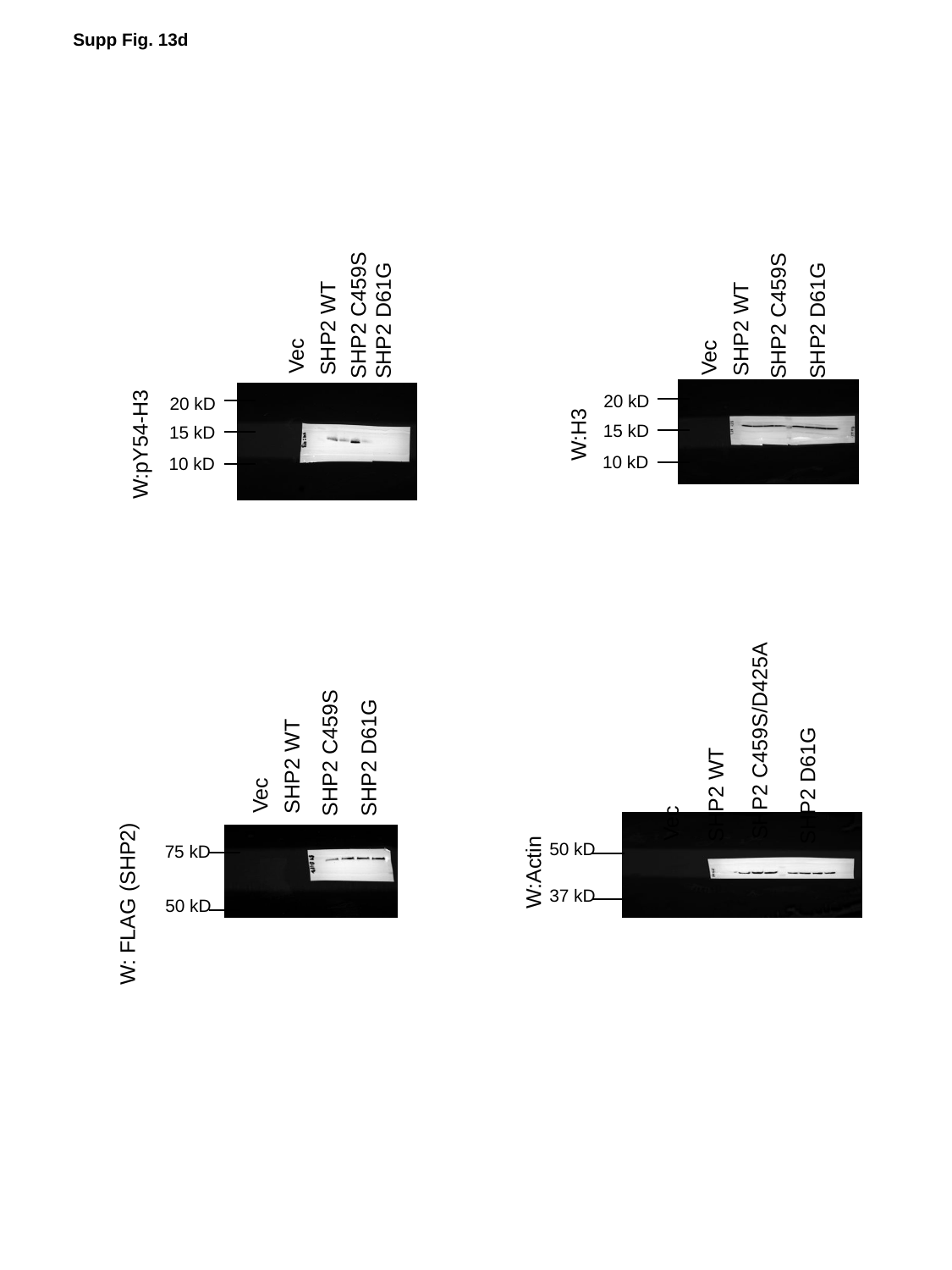

Supp Fig. 13d
SHP2 C459S
SHP2 C459S
Vec
SHP2 WT
SHP2 WT
Vec
SHP2 D61G
SHP2 D61G
W:H3
 20 kD
 20 kD
W:pY54-H3
 15 kD
 15 kD
 10 kD
 10 kD
SHP2 C459S
SHP2 C459S/D425A
SHP2 WT
Vec
SHP2 D61G
SHP2 WT
Vec
SHP2 D61G
W:Actin
 50 kD
 75 kD
W: FLAG (SHP2)
 37 kD
 50 kD

## Slide 54
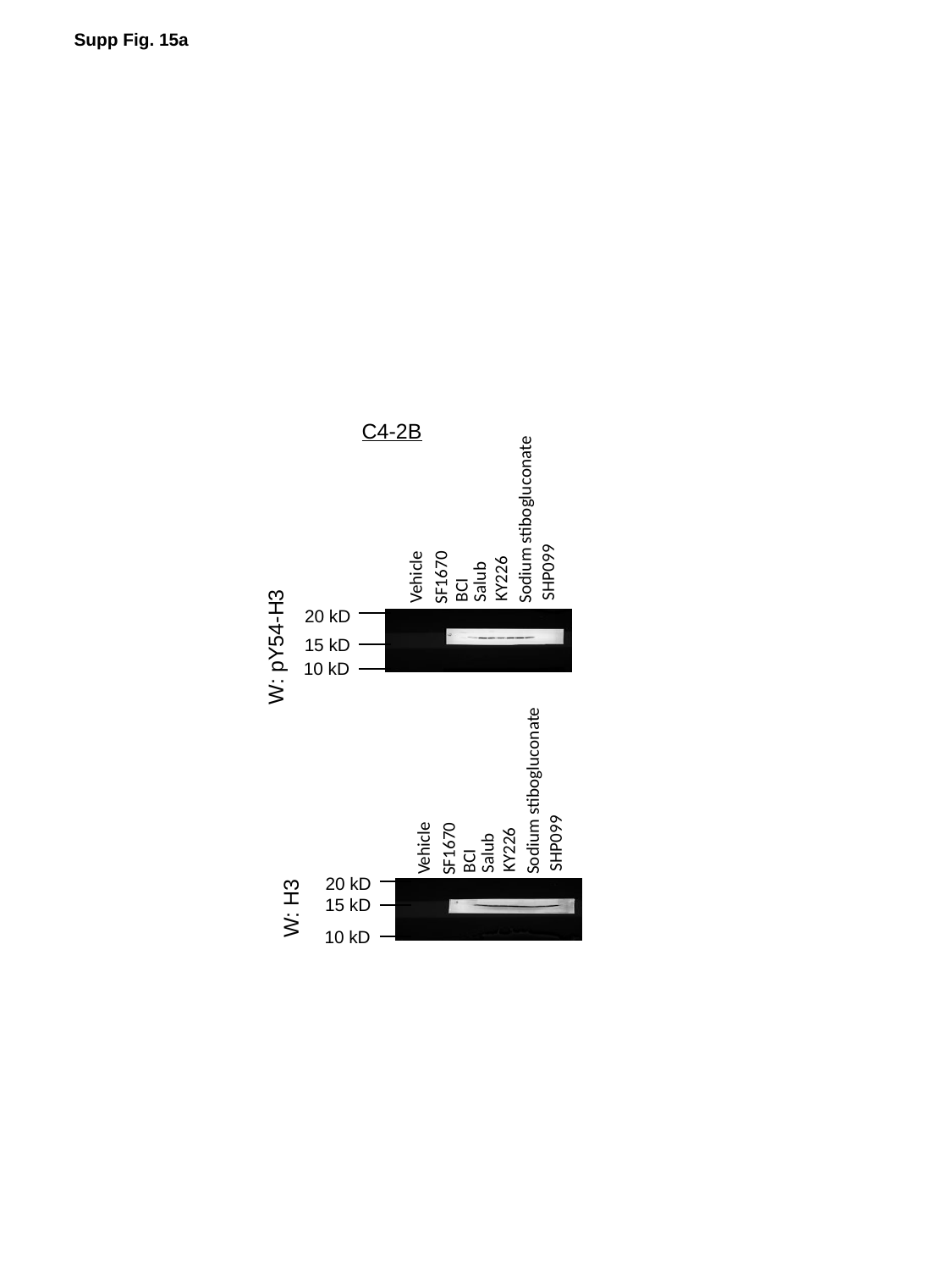

Supp Fig. 15a
C4-2B
Sodium stibogluconate
SHP099
KY226
BCI
Salub
Vehicle
SF1670
 20 kD
W: pY54-H3
 15 kD
 10 kD
Sodium stibogluconate
SHP099
KY226
BCI
Salub
Vehicle
SF1670
W: H3
 20 kD
 15 kD
 10 kD

## Slide 55
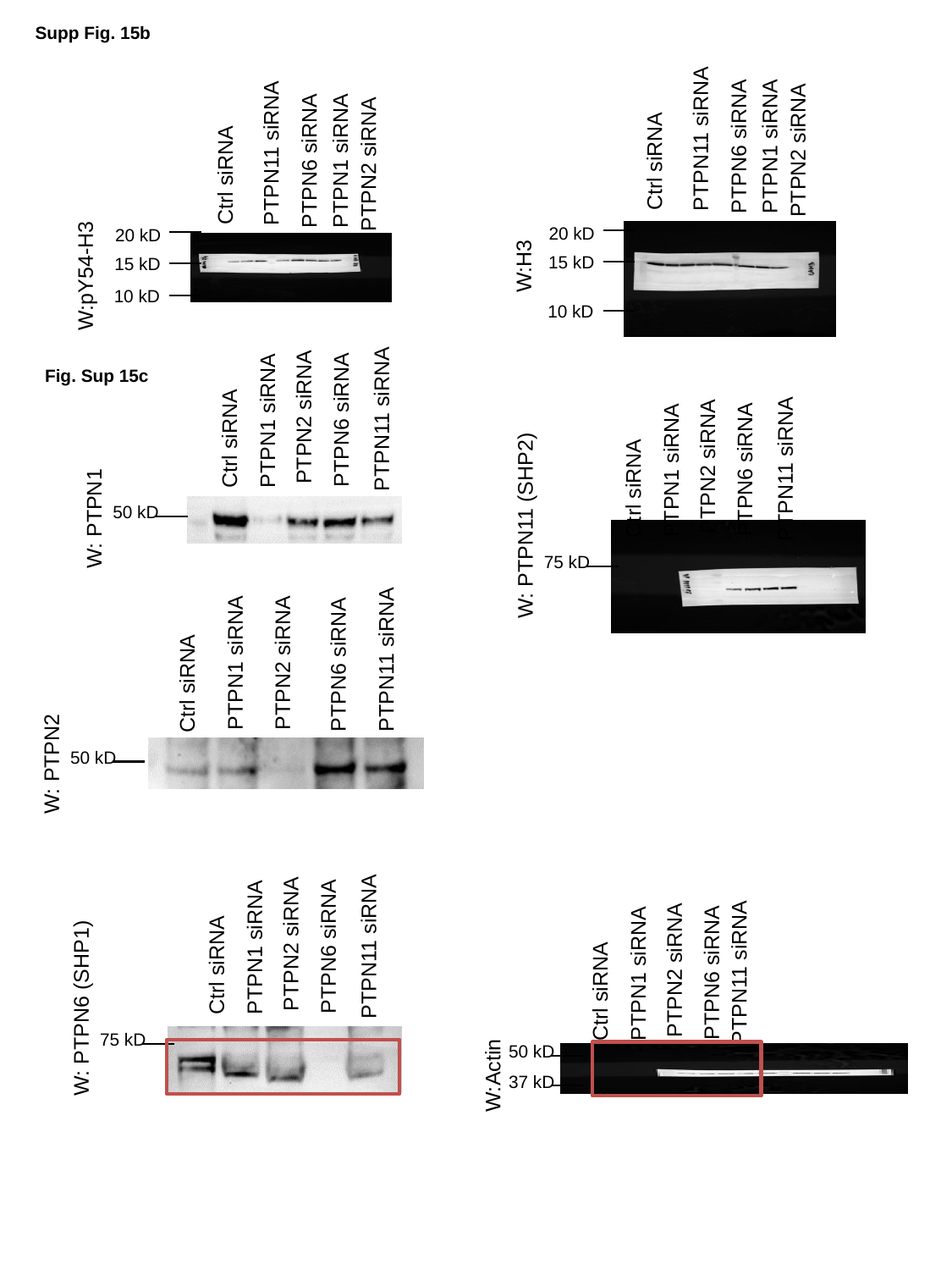

Supp Fig. 15b
PTPN6 siRNA
PTPN1 siRNA
PTPN2 siRNA
PTPN6 siRNA
PTPN1 siRNA
PTPN11 siRNA
PTPN2 siRNA
PTPN11 siRNA
Ctrl siRNA
Ctrl siRNA
W:H3
 20 kD
 20 kD
W:pY54-H3
 15 kD
 15 kD
 10 kD
 10 kD
PTPN2 siRNA
Fig. Sup 15c
PTPN6 siRNA
PTPN1 siRNA
PTPN11 siRNA
Ctrl siRNA
PTPN2 siRNA
PTPN6 siRNA
PTPN1 siRNA
PTPN11 siRNA
W: PTPN1
Ctrl siRNA
 50 kD
W: PTPN11 (SHP2)
 75 kD
PTPN1 siRNA
PTPN2 siRNA
PTPN6 siRNA
PTPN11 siRNA
Ctrl siRNA
W: PTPN2
 50 kD
PTPN2 siRNA
PTPN6 siRNA
PTPN1 siRNA
PTPN11 siRNA
PTPN2 siRNA
PTPN6 siRNA
PTPN1 siRNA
Ctrl siRNA
PTPN11 siRNA
Ctrl siRNA
W: PTPN6 (SHP1)
 75 kD
W:Actin
 50 kD
 37 kD
